# Identification of the core regulators of the HLA I-peptide binding process

**DOI:** 10.1038/srep42768

**Published:** 2017-02-17

**Authors:** Yu-Hang Zhang, Zhihao Xing, Chenglin Liu, ShaoPeng Wang, Tao Huang, Yu-Dong Cai, Xiangyin Kong

**Affiliations:** 1 Institute of Health Sciences, Shanghai Institutes for Biological Sciences, Chinese Academy of Sciences; University of Chinese Academy of Sciences, Shanghai 200031, People’s Republic of China; 2 School of Life Sciences and Biotechnology, Shanghai Jiaotong University, Shanghai 200240, People’s Republic of China; 3 School of Life Sciences, Shanghai University, Shanghai 200444, People’s Republic of China

## Abstract

During the display of peptide/human leukocyte antigen (HLA) -I complex for further immune recognition, the cleaved and transported antigenic peptides have to bind to HLA-I protein and the binding affinity between peptide epitopes and HLA proteins directly influences the immune recognition ability in human beings. Key factors affecting the binding affinity during the generation, selection and presentation processes of HLA-I complex have not yet been fully discovered. In this study, a new method describing the HLA class I-peptide interactions was proposed. Three hundred and forty features of HLA I proteins and peptide sequences were utilized for analysis by four candidate algorithms, screening the optimal classifier. Features derived from the optimal classifier were further selected and systematically analyzed, revealing the core regulators. The results validated the hypothesis that features of HLA I proteins and related peptides simultaneously affect the binding process, though with discrepant redundancy. Besides, the high relative ratio (16/20) of the amino acid composition features suggests the unique role of sequence signatures for the binding processes. Integrating biological, evolutionary and chemical features of both HLA I molecules and peptides, this study may provide a new perspective of the underlying mechanisms of HLA I-mediated immune reactions.

Human leukocyte antigen (HLA), which refers in particular to the major histocompatibility complex (MHC) in humans, is a group of cell surface proteins that is essential for the recognition of self-cells and non-self-cells and activation of the acquired immune system to eliminate the non-self-components^1^,^2^. By restricting cytotoxic immune responses, HLA molecules induce immune recognition of cytosol antigens by presenting endogenous or exogenous antigens^3^ during specific interactions between leukocytes (which are immune cells) and other leukocytes or somatic cells, which further determine the specificity, function and responsiveness of effector T cells. Based on the genetic traits, the HLA family can be divided into three main subgroups (HLA I, HLA II and HLA III), two of which contribute to the immune recognition: the HLA I molecules, which occur on all nucleated cells, and the HLA II molecules, which are mainly presented on antigen-presenting cells (APCs), contributing to the presentation of immune epitopes to cytotoxic T cells (CD8^+^ T cells)^4^,^5^.

Cytotoxic T cells which are activated by interacting with HLA I-peptide complex trigger programmed cell death and also mediate cellular immunity against intracellular pathogens and pathological cells. Therefore, comparing to HLA II molecules, the HLA I molecules which directly present the endogenous antigens may be a group of functional immune regulatory molecules in cellular immunity^6^. During optimal generation (cleavage of antigens), selection (interactions between cleaved peptides and HLA I proteins regulated by chaperone tapasin) and presentation (transport HLA-I complex from endoplasmic reticulum to membrane surface) processes, the cleaved and transported antigenic peptides have to persistently and stably bind to HLA-I protein for further immune recognition^6^,^7^,^8^. The binding affinity between peptide epitopes and HLA I molecules may be the priority among priorities for endogenous cytosol antigen recognition. Apart from the functional significance, the sequential characteristics also make it convenient and suitable for computational analysis: after complicated cleavage (by proteasome) and transport (by transporter associated with antigen processing) processes, the peptides that bind to HLA I molecules have a restricted length (8–10 amino acid), improving the efficacy and accuracy for further analyses and binding predictions^9^. Therefore, in this study, we chose to examine the binding processes between peptides and HLA I molecules, but not HLA II molecules, for further analyses and predictions.

HLA I molecules elicit immune responses to endogenous antigens by binding to cleaved and transported peptides, and the binding affinity is one of the essential regulatory factors for effective immune recognition, as we have analyzed above^10^. HLA I molecules with higher affinity to peptides can easily recognize the endogenous antigens and further activate antigen-specific cellular immunity^11^,^12^. Various endogenous and exogenous factors have been reported to contribute to the specific binding of peptides to the HLA I molecules^13^, such as the HLA I genotypes and peptide sequence features. HLA I genotypes, which further affect the isotypes of HLA I molecules, have been reported to be related to the different recognition activities for specific antigens among various populations^14^,^15^. For example, enteric infection has been reported to exist extensively among various populations^16^. However, it has also been confirmed that different HLA I genotypes may mediate different immune reactions against the same pathogens responsible for specific enteric infections, such as *S. e. typhimurium*^14^. In addition to the HLA I genotypes, it has been confirmed that different peptide sequences have different binding affinities to the HLA I molecules and even to the same HLA I molecule^17^. Non-synonymous mutation is a typical way to alter the given amino acid sequence of a specific protein/antigen and may also change the immunogenicity of a given antigen, affecting the associated immune reactions^17^. In ovarian cancer, various antigenic peptides containing tumor specific non-synonymous mutations (e.g. HSDL1 L25V) have been confirmed to be recognized by binding to HLA I molecules with high affinity in ovarian cancer cells, whereas the wild-type amino acid sequences appear to have relatively low binding affinity for the HLA I molecules^18^. Therefore, the features of both the HLA I molecules and the peptides may simultaneously contribute to the specific antigen presentation processes and further affect the HLA I-induced immune reactions.

Although various influential factors have been revealed to contribute to the specific peptide generation, binding selection and presentation processes of HLA-I/peptide complexes and related immune reactions against endogenous antigens, the principle factors determining the peptide binding specificity to different HLA I molecules during various biological stages have not been fully discovered. In this paper, we proposed a new computational method to discover the core regulators for HLA-I/peptide binding affinity during the integrated peptide generation, binding selection and presentation processes based on the Immune Epitope Database (IEDB)^19^. This data set has been validated to integrate various immune-associated databases, including more than 95% of the manually curated, relevant published literature, aggregating more than 15,000 journal articles and more than 704,000 experiments. This database is the most comprehensive HLA binding-associated database to date, although it may still be limited and require real time updates. Our method, for the first time, extracted all experimentally verified HLA I-associated peptides and related HLA I isotypes from the IEDB database, and separated the data as high-affinity/low-affinity interactions using an IC50 binding threshold of 50 nM^20^. The selection of such threshold mainly relied on recent publications and related database: (1) According guidelines for selecting thresholds of IEDB database, peptides with IC50 values <50 nM are considered high affinity, indicating 50 nM as a proper threshold^20^; (2) Pre-existing computational algorithms like *NetMHC, ANN* and *NetMHCpan*, that contribute to HLA binding predictions and analysis all select 50 nM as an appropriate cut-off to identify high binding peptides^21^,^22^; (3) Various experimental trials on HLA binding processes involving multiple study areas including tumor immunotherapy, autoimmune diseases, etc. also select 50 nM as a specific marginal value for high affinity interactions^17^,^18^. Further, after the separation, each interaction was encoded by 340 different features related to both the amino acid composition (AAC) and the pseudo-amino acid composition (Pse-AAC) characteristics of HLA I molecules and the bound peptides. The maximal-relevance-minimal-redundancy (mRMR) and incremental feature selection (IFS) methods were then performed, based on the protein features, and a set of core factors that may affect the affinity of a biogenic or artificial peptide to certain HLA I molecules were discovered. A comprehensive analysis of the results revealed the underlying mechanisms following which the peptides bound to HLA I molecules. This study may provide a new perspective for developing studies of HLA I-mediated immune reactions.

## Materials and Methods

### HLA I-peptide class I binding data collection

The HLA I-peptide binding data were downloaded from the IEDB Analysis Resource^19^, (http://tools.iedb.org/static/main/binding_data_2013.zip). The database contains the largest MHC I-peptide binding dataset to date and is based on manually curated literature references. Amino acid sequences of the HLA I alleles were extracted from the IPD-IMGT/HLA Database^23^ (release 3.24.0, ftp://ftp.ebi.ac.uk/pub/databases/ipd/imgt/hla/). The peptide sequences were annotated using BLAST+ (version 2.3.0) by comparing them against the Ensembl GRCh37 protein sequences (http://ftp.ensembl.org/pub/release75/fasta/homo_sapiens/pep/Homo_sapiens.GRCh37.75.pep.all.fa), and only the perfect matches that had 100% similarity with the current protein records were retained.

The dataset used for the analysis was finally constructed and included 344 proteins and 88 HLA I alleles, with an average of 38 affinity measurements and a total of 3,361 measurements. The data were divided into two groups according to the IC50 values, namely, the high-affinity (IC50< = 50 nM) and low-affinity (IC50 > 50 nM) groups. The description of each HLA I-peptide interaction pair and its binding affinity are listed in Supplementary Material S1.

### Feature composition

Every HLA I-peptide interaction pair was encoded based on both the HLA I protein features and peptide sequence features. Two types of protein features were adopted in this study: the amino acid composition (AAC) and pseudo-amino acid composition (Pse-AAC) features. Their brief descriptions are as follows.

### Amino acid composition (AAC)

AAC encodes a protein using a 20-dimentional vector, with each element representing the occurrence frequency of one of the 20 standard amino acids in the sequence. AAC is a widely used type of feature that is closely related to various protein attributes, including protein structures and functional properties. In this paper, each binding pair was encoded by the AAC features of both the HLA I protein and the peptide sequences.

### Pseudo-amino acid composition (Pse-AAC)

In addition to the AAC features used to obtain the occurrence frequency information, Pse-AAC features were also adopted, which represent the neighboring and ordering effects of HLA I and the peptide sequences. The concept of Pse-AAC was first introduced by Chou *et al*. to improve protein type prediction. Sequence order effects were described as a set of rank-different correlation factors based on physicochemical distance^24^,^25^,^26^,^27^,^28^, and the number of factors was set as *λ*. A weight *w* for the sequence order effect was included to balance its impact and the impact of the occurrence frequencies.

In this study, five types of physicochemical and biochemical properties of amino acid residues were adopted, including (1) codon diversity (CD), (2) electrostatic charge (EC), (3) molecular volume (MV), (4) secondary structure (2^nd^_stru) and (5) polarity^29^,^30^. The parameters *w* and *λ* were set to 0.15 and 50 for proteins, respectively, and 0.15 and 10 for peptides, respectively. Eventually, the binding pair was encoded by 250 (5 × 50) Pse-ACC features of the HLA I protein and an additional 50 (5 × 10) features of the peptide sequence.

Finally, after combining both the AAC and Pse-AAC features, the HLA I-peptide interaction pair was encoded as a 270-dimensional (20 + 250) vector for the HLA I protein termed *V*_pro_ and an additional 70-dimentional (20 + 50) vector for each peptide termed *V*_pep_. The 340-dimentional feature vector was constructed for each interaction pair and was represented as *V*, where *V* = *V*_pro_ + *V*_pep_. The feature compositions for the proteins and peptides were listed in Table 1.

### Feature selection

The extracted features for the HLA I-peptide interaction pair were ranked in descending order using the maximal-relevance-minimal-redundancy (mRMR) method. The optimal feature subset that primarily affected the binding affinities were selected using the incremental feature selection (IFS) method.

As a mutual-information based method, the goal of mRMR method is to rank the features such that the top features have maximal relevancy to the target class and minimal redundancy to the previously selected features. For two features *x* and *y*, their proximity in feature space can be measured by their mutual information which is formulated as follows:


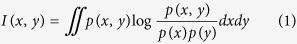


where *p*(*x, y*) denotes joint probabilistic density of *x* and *y, p*(*x*) and *p*(*y*) denote the marginal probabilistic density of two variables *x* and *y*, respectively

In the procedure of feature ranking, the relevancy of a given feature to the target class is determined as the mutual information between the feature and target class, and the redundancy of the same feature is defined as the average mutual information with features selected before it. When mRMR method was executed on each feature in feature vector, two feature lists were obtained based on two criteria: the MaxRel feature list based on the maximal relevancy criterion and the mRMR feature list based on the combination of maximal relevancy and minimal redundancy criteria. Usually, top *m* features in MaxRel feature list can be selected from the feature vector and utilized to construct the classifier based on an algorithm. However, according to the researches on feature ranking^31^,^32^,^33^, the classifier derived from the top m features in MaxRel feature list are not always leading to the optimal performance because the redundancies exist between them. Therefore, in the mRMR feature list, the redundancy between features is also considered to evaluate the importance of each feature. In this study, the ranking order of features in mRMR feature list was used to construct classifiers.

The obtained MaxRel and mRMR feature lists with 340 features in them have similar form, which can be formulated in the following Eq. 2:


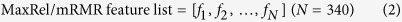


It is clear that the order of single feature in feature list is not enough to construct classifiers and select an optimal classifier from them. Thus, an incremental greedy strategy called IFS was used to build a series of feature sets. In give feature set, top *i* features in mRMR feature list were selected and these features would consist of a feature set *S*_*i*_, which can be formulated as follows:


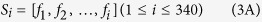


Finally, 340 feature sets, denoted as *S*_1_, *S*_2_,…, *S*_340_, were constructed and each set has one more feature compared to the former one. Based on these feature sets, a series of classifiers were built using one of the four algorithms described in the following section. For these classifiers derived from different algorithms with different number of features, the classifier obtained the best prediction performance would be selected as the optimal classifier and the corresponding features in the feature set are called optimal features. This process is called the IFS method and has been widely used in previous studies^34^,^35^.

### Classification algorithms

Four machine learning algorithms were adopted to construct the model and determine the proper approach for classification. The optimal algorithm was determined based on the best classification performances. The four algorithms, Dagging^36^, nearest neighbor algorithm (NNA), random forest (RF)^37^, and support vector machine (SVM)^38^, are described below.

### Dagging

This algorithm is applied to construct several meta classifiers derived from a single dataset, in which the predicted result for given sample is decided by majority voting. For a training dataset with *N* samples, *m* subsets (*m* should be an odd number) would be extracted, with each having *n* randomly selected samples using the sampling without replacement technique, where *mn* ≤ *N*. Therefore, *m* basic classifiers (*C*_1_, *C*_2_,…, *C*_m_) would be trained on the *m* subsets of the original training dataset. As introduced here, the classification result on a query sample is voted on using all of the basic classifiers’ results.

### NNA

This algorithm computes and obtains classification results for given query samples via a distance calculation of the samples’ feature spaces. Because there is no training process, the NNA algorithm has a relatively faster computation efficiency than the algorithms that execute the training step. For a query sample in same feature space that was defined by the training samples, the smallest distance is measured using distance matrices, such as Euclidean distance and Hamming distance, between the query sample and a given training sample. Then, the class of the nearest training sample is directly assigned to the query sample. The process is repeated until all the query samples obtain the predicted results.

### RF

As an ensemble learning algorithm, hundreds of decision trees can be constructed from a single training dataset. All the classification trees that comprise the so-called decision forest are used to generate predicted results for a given query sample, in which the final result is dependent on the results with the most votes. Two innovative techniques, namely bootstrap aggregating that selects samples from the original training dataset by replacement and random selection of sub-features, are used to construct decision trees, largely increasing the predictive accuracy of the classifiers obtained from the algorithm.

### SVM

Since it was first proposed in 1995, the SVM algorithm has been widely utilized to solve the problem of classification, regression and pattern recognition in many fields, which is particularly applicable to the statistical analyses of small scale samples. By mapping the samples into higher-dimensional feature space, an optimal splitting hyper-plane would be obtained to decide the class of the query sample by relying on the side of the hyper-plane to which it belongs. In this study, the sequential minimal optimization (SMO) technique was used as a way to construct the SVM classification model; the implementation details of which can be found in Platt’s study^39^.

As a widely used suit of software, the Weka software^40^ that collects several state-of-art machine learning algorithms was utilized in this study. All of the four algorithms were applied using with the classifiers named Dagging, IB1, Dagging and RandomRorest, and SMO in Weka. At the same time, the default parameters of corresponding classifiers were adopted to build classifiers based on feature sets introduced above.

### Performance evaluation

Four widely applied measurements were utilized for the performance evaluation, including sensitivity (*SN*), specificity (*SP*), accuracy (*ACC*) and Matthew’s correlation coefficient (*MCC*). The definitions of the four measurements are illustrated in Eqs 3,4,5,6:


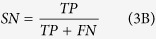



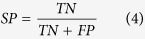



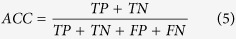



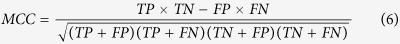


where *TP, TN, FP* and *FN* are the two-letter abbreviations for true positive, true negative, false positive and false negative, respectively.

Ten-fold cross validations were performed on the dataset. Because *MCC* is considered as the most comprehensive measurement to represent the performances of the classifiers constructed using an imbalanced dataset, it was selected to evaluate the classifiers’ predictive abilities and to select the optimal classifier based on a specific algorithm.

## Results

The ten-fold cross-validation^41^ is used for the evaluation process, and four types of evaluation measurements are calculated according as we have mentioned above to evaluate the objective classification ability of the given classifier. The 340-dimensional features are ranked according to the two criteria: the features that are evaluated by maximal relevance are listed in the MaxRel feature list, and the features that are ordered by the maximal relevance and minimal redundancy are listed in the mRMR feature list. The contents of the two lists are provided in Supplementary Material S2.

**Figure 1:**
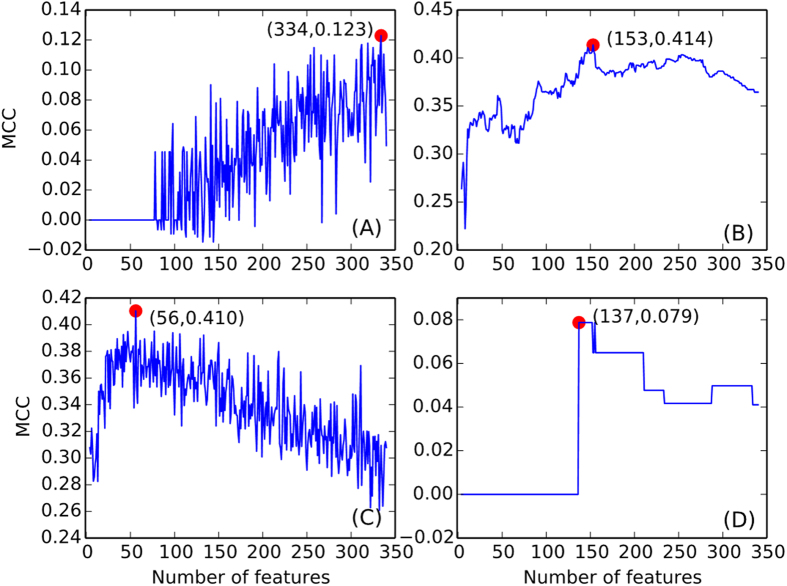
The IFS-curves for the (**A**) Dagging, (**B**) NNA, (**C**) RF and (**D**) SVM algorithms, with the best performance marked with red point. The X-axis indicates the number of features used to construct the classifiers, and the Y-axis indicates their corresponding *MCC* values.

Additionally, the IFS method is performed on the features of the mRMR feature list, and 340 IFS feature sets are obtained by individually adding the ranked features. Each feature set is used to construct one classifier, and the ten-fold cross-validation is performed to compare the classification performances. The IFS-curves for the classifiers based on the four algorithms are illustrated in Fig. 1. The X-axis of the IFS-curve represents the number of features in the IFS feature sets, whereas the Y-axis shows the *MCC* values of the corresponding classifiers. The best algorithm and optimal feature sets that can achieve the best *MCC* for the classification are determined. As shown in Fig. 1B, the best classifier is marked with a red point on its IFS curve which is derived from the NNA algorithm with the top 153 features. This classifier achieves a better performance, with *MCC* = 0.414, compared to all other algorithms (Dagging: 0.123, RF: 0.410, and SVM: 0.097). Therefore, in this study, the NNA algorithm is deemed to be the representative algorithm for predicting the binding affinity between HLA I molecules and peptides. In addition to the *MCC* values, three other evaluation measurements based on each optimal classifier of the four algorithms are listed in Table 2. Detailed evaluation results based on all IFS features sets, and the four algorithms are provided in Supplementary Material S3.

**Figure 2:**
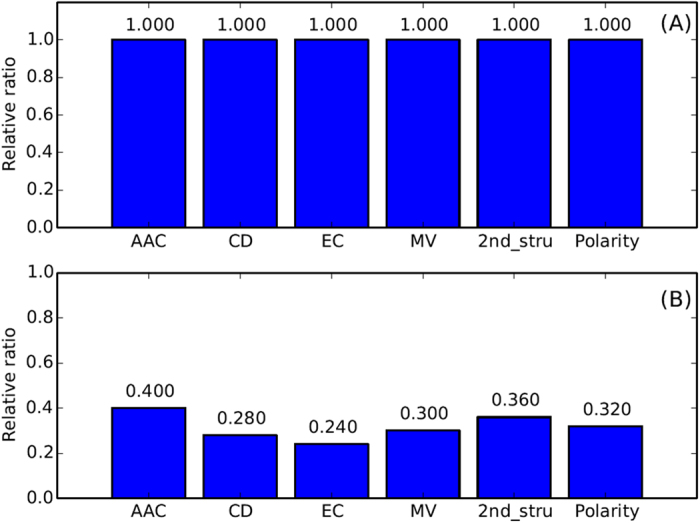
The distributions of the relative ratios for the top 153 features derived from the mRMR feature list for (**A**) the peptide features and (**B**) the HLA I protein features.

**Figure 3:**
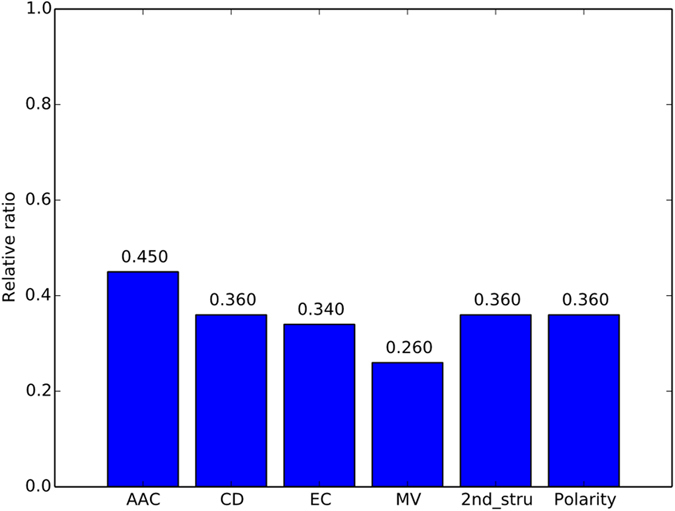
The distribution of the relative ratios for the top 93 features derived from the MaxRel feature list for the HLA I protein features.

Furthermore, the 153 IFS feature sets are divided according to the feature types, as illustrated in Fig. 2. As shown in Fig. 2A, all 70 peptide features are listed in the top 153 features, with all relative ratios equal to 1. Regarding the selected protein features, 8 features belong to AAC, and 14, 12, 15, 18 and 16 features belong to CD, EC, MV, 2^nd^_stru, and polarity, respectively. The relative ratios are shown in Fig. 2B. Similar approaches are performed on the MaxRel feature list, and the top 93 features are selected for further study. It was worth noting that all these top 93 features are protein features, and the relative ratio for each feature type is illustrated in Fig. 3. For the first time, we revealed the potential role of the evolutionary (codon diversity) and chemical (electrostatic charge) characteristics for the interactions between HLA I molecules and the interacting peptides. Further detailed biological analyses of the top 153 and 93 features derived from the two feature lists are discussed below.

## Discussion

Based on the presented computational methods, for the first time, we thoroughly analyzed six aspects of the biological, evolutionary and chemical features of both the MHC alleles and related peptides, not only the sequence features, as was performed in previous reports^22^,^42^. A group of features obtained from the comprehensive analysis is screened out, which may directly affect the binding affinity of the HLA I molecules for the related peptides during multiple biological stages including generation, selection and presentation.

The prediction results validate the hypothesis that the features of the HLA I molecules and related peptides simultaneously affect the binding processes. In detail, first, all of the top 93 features of the MaxRel feature list (sort each feature according to its contributions to classification) were associated with the HLA I proteins, implying that the HLA isotype is an important factor that affects HLA-peptide binding processes^43^. Furthermore, after the feature redundancy is removed during the ranking process (based on the mRMR computational method), the peptide features dominate the core regulatory factors, with 27/34 features in the top 10 percent of features. Amino acid characteristics (16/34) seem to be the most significant features for the interactions between HLA I molecules and the related peptides, with a quite high relative ratio of 80% (16/20). This result implies that the characteristics of the peptide, particularly the amino acid composition features of the peptides, may contribute more to the biological HLA binding processes^43^.

In summary, features that describe the HLA I antigen binding cleft substantially contributed to the interactions between HLA I molecules and the related peptides, although with considerable redundancy. Although a single peptide characteristic exhibits a relatively low correlation with the binding affinity, based on MaxRel feature list, the peptide features include less redundancy and have more features located in the top 10 percent of the mRMR feature list (removing the redundancy). Comprehensively, the amino acid composition features of the peptides are the principle factors that contribute to these biological processes. This conclusion is supported and validated by various recent publications, implying the accuracy and efficacy of our analysis.

### HLA protein features

Based on the MaxRel features (without removal of redundancy), HLA proteins seem to be more directly related to the HLA binding affinity (these proteins contribute more to the distinction of peptides with high and low binding affinity), although with more redundancy, which is supported by recent publications. The top 153 features are extracted from the prediction results (after redundancy removal), among which 83 features contribute to the HLA I isotypes, to identify the key regulatory factors of HLA I molecules that contribute to the HLA I-peptide binding affinity. Among these 83 features, 40% (relative ratio) of all HLA I allele amino acid composition (AAC) features, which mainly describe the otherness of different HLA I alleles, are present in the top 153 features, implying the significant role of HLA I isotypes for the interactions between HLA I molecules and related peptides.

### Amino Acid Composition (AAC) features of HLA proteins

Based on recent publications, it has been confirmed that specific HLA isotypes with characteristic (sequence feature of) antigen binding clefts may contribute to the differences in immune reaction against similar antigens (most autologous), inducing a different susceptibility to certain diseases^14^,^44^,^45^. Using a specific disease, ankylosing spondylitis (AS) as an example. It has been reported that a specific HLA I isotype, HLA-B27, varies remarkably between races and may contribute to the differences in susceptibility to various immune-associated diseases, including AS, in multiple races^45^,^46^. Despite the controversies about the pathogenic mechanisms, the *arthritogenic peptide hypothesis*, one of the acknowledged pathological mechanisms, attributes the initiation of the disease to the cross-reaction of HLA-B27 and self-peptide pairs, and the differences in incidence may be induced by the differences in the HLA I-peptide binding affinities of different HLA I isotypes, suggesting that specific characteristics of HLA I alleles contribute to the HLA-I mediated immune recognition^47^. Considering that the binding affinity between antigenic peptides and HLA-I molecules turn out to be the principle regulatory factor for such immune recognition, it’s quite reasonable to demonstrate that the optimal features of HLA-I molecules may definitely affect binding processes between HLA-I molecules and related peptides.

In addition, previous studies screened out tumor specific antigens and further designed functional vaccines that can specifically recognize tumor cells to create so-called precise treatment during personalized immune therapy for cancer^48^,^49^. It has been reported that both the tumor-specific antigen and the related reactive HLA alleles should be personalized to achieve the best therapeutic effects. For example, in a recent publication, the specific peptide RVFAJSFIK has been reported to bind to the HLA-A11 allele with high affinity, but bound other HLA I alleles (e.g., HLA-A1, HLA-A3 and HLA-B7) with low affinity, validating the hypothesis that the binding affinity of the HLA I allele and certain peptides are definitely related to the specific HLA I allele and its specific antigen binding cleft^50^.

### Pseudo-amino acid composition (Pse-AAC) features of HLA proteins

In addition to the amino acid composition features, various pseudo-amino acid composition features have also been screened out to contribute to the interactions between HLA I molecules and related peptides. The secondary structure features of HLA I molecules (ranked 21 and 29 in the top 10 percent of the optimal list) may also be significant for the specific HLA I allele-peptide binding processes. Considering the internal relationship between the HLA I amino acid composition features and certain secondary structures (the primary structures decide the secondary structures), the high correlations between the HLA I allele secondary structure and HLA I-peptide binding affinity may also be quite reasonable^51^,^52^. During the identification of certain foreign antigens, HLA I isotypes with different secondary structures have been validated to have different reactivity against similar antigen-derived peptides^53^.

Regarding polarity (ranked 11 in the top 10 percent of the optimal list), specific amino acid features may definitely affect the polarity of the HLA I alleles because the polarity of a certain protein may be associated with the functional R residue of a certain amino acid. The results imply that the key regulatory factor of HLAI alleles that contributes to interactions between the HLA I allele and its respective peptide may be the specific sequence characteristics, which appear to be different in HLA I isotypes, as validated by our analysis described above^54^.

### High redundancy load in optimal HLA I allele features

Apart from such detailed features we screened out, comprehensively, based on our algorithm, we further identified that the high correlation between HLA I allele features and high HLA binding affinity has specifically high redundancy. There are two main reasons to explain this characteristic phenomenon. First, in our reference database (IEDB database), the same HLA I allele may correspond to multiple peptides, whereas a single peptide may rarely be tested using various HLA I alleles^20^. During the division of the high HLA I binding affinity group and low HLA I binding affinity group, we generally refer to the binding affinity of specific HLA I allele and peptide pairs. Therefore, specific HLA I alleles that have high affinity for one peptide and low affinity for another peptide may be clustered into both groups, inducing considerable redundancy for the HLA I-associated features. In addition, considering that the otherness between different HLA alleles is mainly attributed to specific groups of SNPs and the custom sequences of different HLA alleles have high similarities, most of the screened out features (particularly for the amino acid associated features) are related to each other and may be regarded as redundant when the redundant features are removed because of the linkage inheritance of the HLA district and sequence similarity^55^. Although the two reasons mentioned above induce considerable redundancy in the HLA I allele-associated features, 7 features of the top 34 features that describe the HLA I allele-associated characteristics remain after the redundant features are removed, validating the irreplaceable role of HLA I isotypes in the interactions between the HLA I antigen binding cleft and related peptides.

### Peptide features

Considering that the HLA I antigen binding processes are specific interactions between HLA I molecules and peptides, the optimal features of the peptide may also contribute to these biological processes. Based on our algorithm, we considered 70 peptide-associated features. After screening and removing the redundant features, all 70 peptide-associated features are ranked in the top 153 features list. Based on the MaxRel criterion (sort each feature according to its contributions to the classification), each individual feature of the peptides contributes less to the interactions. However, 27 peptide features (20 features describing the amino acid composition) are included in the top 34 features after removing the redundant features, implying that the features of peptides, particularly the features describing the amino acid composition of peptides, may play a quite significant role in the interactions between HLA I molecules and the related peptides, with very little redundancy^43^.

Based on the classification method we mentioned above, we also classify the amino acid features into six sub-groups (AAC, CD, EC, MV, 2^nd^_structure, and polarity). In the top 10 percent of 340 optimal features (34 features) screened using the mRMR methods, 16 features describe the amino acid composition features of the peptides, implying the specific role of sequence features in contributing to the interactions between HLA I molecules and the related peptides.

### Amino Acid Composition (AAC) features of peptides

Among the six clusters, amino acid-associated features seem to be the most significant regulatory factor that contributes to peptide binding affinity during multiple interaction stages, as we have analyzed above. As we all know, HLA I molecules have a given antigen specificity^56^. Therefore, it is quite reasonable that the amino acid composition features of the peptide are the most significant features for the HLA I-peptide binding processes. For example, it has been reported that during mother-to-child transmission of HCV, 60–80% of infections persist after birth and escape T cell recognition through unique escape mutations on specific HCV antigens^57^. Considering that specific mutations of the HCV antigen have greatly altered the HLA binding affinity and further affect the immune reaction mediated by the HLA I molecule, the specific amino acid sequence described by the amino acid composition features definitely contributes to the interactions between the HLA I molecules and the related peptides, validating our analysis. In addition, according to recent publications of personalized tumor therapy, the mutant and wild-type antigens (peptides) have been confirmed to show enormous differences in HLA binding affinity^58^,^59^. For example, it has been confirmed that the famous mutant RAS p21 proteins bind to HLA I molecules more easily, and activate CD8^+^ restricted T cell responses better than wild-type RAS p21 proteins, validating that certain amino acid composition features of the peptide may definitely contribute to the interactions between HLA I molecules and the related peptides^58^.

### Pseudo-amino acid composition (Pse-AAC) features of peptides

In addition to the amino acid composition features, the other five clusters of features (pseudo-amino acid composition features) may also participate in these biological processes, according to the optimal feature list (top 10 percent of the 340 optimal features). Considering the functional charged residues in the HLA binding pocket, the electrostatic charge signatures of peptides, which are clustered into another group of features (ranked 4 and 16), may also be quite significant for the specific interactions between peptides and certain antigen binding clefts^60^. During the peptide generation, selection and presentation processes, it has been confirmed that the binding pockets of HLA I molecules show specific physico-chemical properties and side-chain selectivity^9^. Considering that the electrostatic charge signatures of peptides can be attributed to the specific side-chains (R residues), the electrostatic charge signatures may definitely contribute to the interactions between HLA I molecules and the related peptides^61^. Three specific codon diversity-associated features (ranked 10, 19, and 34) are also in the top 10 percent of the list of 340 features, implying that the codon diversity of certain antigen-derived peptides may also contribute to the interactions between HLA I molecules and peptides. Three specific secondary structure-associated features (ranked 13, 14, and 29) of the peptide have also been screened and are in the top 10 percent of the optimal features. As we mentioned above, HLA I molecules participate in cellular immunity by identifying exogenous antigens. Using mouse models, it has been confirmed that one of the homologous genes for HLA I molecules in mice, H-2D, shows specific selectivity for the secondary structure of its binding peptide, suggesting the specific role of the peptide secondary structure in MHC I molecule binding affinities^62^. In humans, which are our main object of study, a specific trial revealed the specific secondary structures of two identified peptides, Tax8 (LFGYPVYV) and Tax9 (LLFGYPVYV), which has one additional N-terminal residue, with different affinities for a specific HLA I molecule, HLA-A*02:01, implying that peptides with different secondary structures (similar amino acid sequences) show quite different binding affinities to HLA I molecules, validating the conclusions of our analysis^63^,^64^. Similar to the features of the HLA I molecules, we also obtained a few molecular weight-associated features (ranked 27) that were in the top 10 percent list. Considering that different molecular weights may indicate the different amino acids, it is quite reasonable for us to explain the contribution of molecular weight to the interactions between HLA I molecules and peptides. Polarity-associated features (ranked 18 and 25), which had the highest rank of 18 in our filtered list, may also contribute to the binding affinity of peptides to certain HLA I isotypes. As commonly reported, one of the cores of adaptive immunity is the interactions between the T cell receptor (TCR) on CD8-positive T cells and the peptides presented by HLA I molecules. Based on recent publications, the polarity-dependent recognition mechanism of HLA I molecules and the related peptides is quite essential for further activation of the downstream immune reactions, validating the irreplaceable role of peptide polarity in effective HLA I-peptide binding processes^65^.

Overall, based on our newly presented computational methods, we analyzed and identified a group of core regulatory factors of both the HLA I molecules and the related peptides that may contribute to HLA I-dependent peptide generation, selection and presentation processes According to the results of our analysis, the features of the HLA I molecules show a substantially higher correlation with the HLA I-peptide binding processes, although with more redundancy. After removing the redundant features from the analysis, features of the peptide, particularly the amino acid composition features of the peptides, account for the majority of the top 10 percent of our prediction list, confirming that the features of the peptides, particularly the amino acid composition features of the peptides, seem to be more significant for the processes, with less redundancy. All of our screened high-ranking features have been confirmed by recent publications, as we mentioned above. Unlike the traditional explorations that mainly focus on the amino acid composition of the corresponding peptides, wesimultaneously concentrated on the biological, evolutionary and chemical features of both the HLA I molecules and related peptides to explore the underlying regulatory factors that contribute to HLA I-peptide binding processes. Therefore, supported by these recent publications, our newly presented results may not only partially reveal the underlying influential factors (e.g., the amino acid composition features of the peptide) that contribute to the differences in the binding affinity of HLA I molecules and related peptides and also provide a new perspective for the underlying mechanisms of HLA I-mediated immune reactions.

## Conclusions

The binding affinity between peptide epitopes and HLA proteins have been confirmed to influence the immune recognition ability in human beings. However, the principle factors determining the peptide binding specificity to different HLA I molecules during various biological stages have not been fully discovered.

In this study, a new method for predicting the HLA class I-peptide interactions was proposed. Three hundred forty features of both HLA I proteins and peptide sequences were utilized, including amino acid composition and pseudo-amino acid composition features. The optimal classifier was selected from four powerful machine learning algorithms, and the optimal features were selected based on the maximal-relevance-minimal-redundancy and incremental feature selection methods. The results showed that the HLA I molecule features substantially contribute to the HLA I-peptide binding affinity, but with high redundancy. The peptide features, especially the subgroup features describing the amino acid composition included less feature redundancy and contributed more to the binding affinity after the redundant features were removed. The results further validated the hypothesis that the features of HLA I molecules and the related peptides simultaneously affect the binding process. Founded on systematically analysis, a set of core regulators that contribute to the differences in the binding affinity of the HLA I molecules and peptides were discovered. This study may provide a new perspective of the underlying mechanisms of HLA I-mediated immune reactions.

## Additional Information

**How to cite this article**: Zhang, Y.-H. *et al*. Identification of the core regulators of the HLA I-peptide binding process. *Sci. Rep.* **7**, 42768; doi: 10.1038/srep42768 (2017).

**Publisher's note:** Springer Nature remains neutral with regard to jurisdictional claims in published maps and institutional affiliations.

## Supplementary Material

Supplementary Materials

## Figures and Tables

**Table 1:** The composition of the protein and peptide features for each pair of HLA I-peptide interactions.

	AAC	CD	EC	MV	2^nd^_structure	Polarity	Total
Protein	20	50	50	50	50	50	270
Peptide	20	10	10	10	10	10	70

**Table 2:** The best classification performances based on the optimal classifiers derived from the four algorithms.

Algorithm	Optimal features	*SN*	*SP*	*ACC*	*MCC*
Dagging	334	0.040	0.995	0.875	0.123
NNA	153	0.461	0.936	0.876	0.414
RF	56	0.376	0.961	0.888	0.410
SVM	137	0.007	1.000	0.875	0.079

## References

[b1] Grimholt U. MHC and Evolution in Teleosts. Biology (Basel) 5, doi: 10.3390/biology5010006 (2016). PMC4810163 26797646

[b2] Hanna S. & Etzioni A. MHC class I and II deficiencies. J Allergy Clin Immunol 134, 269–275, doi: 10.1016/j.jaci.2014.06.001 (2014). 25001848

[b3] Garrido F., Romero I., Aptsiauri N. & Garcia-Lora A. M. Generation of MHC class I diversity in primary tumors and selection of the malignant phenotype. International journal of cancer. Journal international du cancer 138, 271–280, doi: 10.1002/ijc.29375 (2016). 25471439

[b4] Galea I. . CD8+T-cell cross-competition is governed by peptide-MHC class I stability. European Journal of Immunology 42, 256–263, doi: 10.1002/eji.201142010 (2012). 22002320 PMC3744744

[b5] Bjorkman P. J. . The Foreign Antigen-Binding Site and T-Cell Recognition Regions of Class-I Histocompatibility Antigens. Nature 329, 512–518, doi: 10.1038/329512a0 (1987). 2443855

[b6] Cresswell P., Ackerman A. L., Giodini A., Peaper D. R. & Wearsch P. A. Mechanisms of MHC class I-restricted antigen processing and cross-presentation. Immunol Rev 207, 145–157, doi: 10.1111/j.0105-2896.2005.00316.x (2005). 16181333

[b7] Watkins D. I., Letvin N. L., Hughes A. L. & Tedder T. F. Molecular-Cloning of Cdna That Encode Mhc Class-I Molecules from a New-World Primate (Saguinus-Oedipus)–Natural-Selection Acts at Positions That May Affect Peptide Presentation to T-Cells. Journal of immunology 144, 1136–1143 (1990). 2104912

[b8] Van Hateren A. . The cell biology of major histocompatibility complex class I assembly: towards a molecular understanding (vol. 76, pg 259, 2010). Tissue Antigens 76, 428–428, doi: 10.1111/j.1399-0039.2010.01577.x (2010). 21050182

[b9] Zhang C., Anderson A. & DeLisi C. Structural principles that govern the peptide-binding motifs of class I MHC molecules. Journal of Molecular Biology 281, 929–947, doi: 10.1006/jmbi.1998.1982 (1998). 9719645

[b10] Liu T. . TAP peptide transporter-independent presentation of heat-killed Sendai virus antigen on MHC class I molecules by splenic antigen-presenting cells. J Immunol 159, 5364–5371 (1997). 9548476

[b11] Andreatta M. . Accurate pan-specific prediction of peptide-MHC class II binding affinity with improved binding core identification. Immunogenetics 67, 641–650, doi: 10.1007/s00251-015-0873-y (2015). 26416257 PMC4637192

[b12] Fisette O., Wingbermuhle S., Tampe R. & Schafer L. V. Molecular mechanism of peptide editing in the tapasin-MHC I complex. Sci Rep 6, 19085, doi: 10.1038/srep19085 (2016). 26754481 PMC4709564

[b13] Sadegh-Nasseri S. & Kim A. Exogenous antigens bind MHC class II first, and are processed by cathepsins later. Mol Immunol 68, 81–84, doi: 10.1016/j.molimm.2015.07.018 (2015). 26254987 PMC4623955

[b14] Kubinak J. L. . MHC variation sculpts individualized microbial communities that control susceptibility to enteric infection. Nature Communications 6, doi: 10.1038/ncomms9642 (2015). PMC4621775 26494419

[b15] Bjorkman P. J. & Parham P. Structure, Function, and Diversity of Class-I Major Histocompatibility Complex-Molecules. Annual Review of Biochemistry 59, 253–288, doi: 10.1146/annurev.biochem.59.1.253 (1990). 2115762

[b16] Jarquin C. . Population Density, Poor Sanitation, and Enteric Infections in Nueva Santa Rosa, Guatemala. Am J Trop Med Hyg 94, 912–919, doi: 10.4269/ajtmh.15-0555 (2016). 26856919 PMC4824239

[b17] Birk M., Vahlne A., Sonnerborg A. & Sallberg M. Nonsynonymous mutations within the human immunodeficiency virus type 1 p17 gene are clustered to sequences binding to the host human leukocyte antigen class I molecules. Aids Res Hum Retrov 14, 241–248, doi: 10.1089/aid.1998.14.241 (1998). 9491914

[b18] Wick D. A. . Surveillance of the Tumor Mutanome by T Cells during Progression from Primary to Recurrent Ovarian Cancer. Clinical Cancer Research 20, 1125–1134, doi: 10.1158/1078-0432.Ccr-13-2147 (2014). 24323902

[b19] Kim Y. . Immune epitope database analysis resource. Nucleic Acids Res 40, W525–530, doi: 10.1093/nar/gks438 (2012). 22610854 PMC3394288

[b20] Vita R. . The immune epitope database (IEDB) 3.0. Nucleic Acids Res 43, D405–412, doi: 10.1093/nar/gku938 (2015). 25300482 PMC4384014

[b21] Lundegaard C. . NetMHC-3.0: accurate web accessible predictions of human, mouse and monkey MHC class I affinities for peptides of length 8-11. Nucleic Acids Res 36, W509–512, doi: 10.1093/nar/gkn202 (2008). 18463140 PMC2447772

[b22] Nielsen M. . Reliable prediction of T-cell epitopes using neural networks with novel sequence representations. Protein Science 12, 1007–1017 (2003). 12717023 10.1110/ps.0239403 PMC2323871

[b23] Robinson J.. The IPD and IMGT/HLA database: allele variant databases. Nucleic Acids Res 43, D423–431, doi: 10.1093/nar/gku1161 (2015). 25414341 PMC4383959

[b24] Chou K. C. Prediction of protein cellular attributes using pseudo-amino acid composition. Proteins 43, 246–255 (2001). 11288174 10.1002/prot.1035

[b25] Chou K. C. & Shen H. B. Cell-PLoc: a package of Web servers for predicting subcellular localization of proteins in various organisms. Nat Protoc 3, 153–162, doi: 10.1038/nprot.2007.494 (2008). 18274516

[b26] Ding Y. S., Zhang T. L. & Chou K. C. Prediction of protein structure classes with pseudo amino acid composition and fuzzy support vector machine network. Protein and peptide letters 14, 811–815 (2007). 17979824 10.2174/092986607781483778

[b27] Chou K. & Shen H. Recent progress in protein subcellular location prediction. Analytical Biochemistry 370, 1–16 (2007). 17698024 10.1016/j.ab.2007.07.006

[b28] Chen L., Chu C., Huang T., Kong X. & Cai Y. D. Prediction and analysis of cell-penetrating peptides using pseudo-amino acid composition and random forest models. Amino Acids 47, 1485–1493, doi: 10.1007/s00726-015-1974-5 (2015). 25894890

[b29] Atchley W. R., Zhao J., Fernandes A. D. & Druke T. Solving the protein sequence metric problem. Proc Natl Acad Sci USA 102, 6395–6400, doi: 10.1073/pnas.0408677102 (2005). 15851683 PMC1088356

[b30] Rubinstein N. D., Mayrose I. & Pupko T. A machine-learning approach for predicting B-cell epitopes. Molecular immunology 46, 840–847 (2009). 18947876 10.1016/j.molimm.2008.09.009

[b31] Andrew A. M. STATISTICAL PATTERN RECOGNITION, by Andrew Webb, Arnold, London (Cambridge University Press, New York, for USA), 1999, xviii+454 pp., ISBN 0-340-74164-3 (pbk, £29.99). Robotica 18, 219–223, doi: null (2000).

[b32] Jain A. K., Duin R. P. W. & Mao J. Statistical Pattern Recognition: A Review. IEEE Transactions on Pattern Analysis & Machine Intelligence 22, 4–37 (2000).

[b33] Cover T. M. The Best Two Independent Measurements Are Not the Two Best. Systems Man & Cybernetics IEEE Transactions on SMC–4, 116–117 (1974).

[b34] Li B. Q., Feng K. Y., Chen L., Huang T. & Cai Y. D. Prediction of protein-protein interaction sites by random forest algorithm with mRMR and IFS. PLoS One 7, e43927, doi: 10.1371/journal.pone.0043927 (2012). 22937126 PMC3429425

[b35] Chen L., Chu C. & Feng K. Predicting the types of metabolic pathway of compounds using molecular fragments and sequential minimal optimization. Combinatorial Chemistry & High Throughput Screening 19, 136–143 (2016). 26552441 10.2174/1386207319666151110122453

[b36] Ting K. M. & Witten I. H. In Proceedings of the Fourteenth International Conference on Machine Learning 367–375 (Morgan Kaufmann Publishers Inc., 1997).

[b37] Breiman L. Random forests. Machine learning 45, 5–32 (2001).

[b38] Corinna Cortes V. V. Support-vector networks. Machine Learning 20, 273–297 (1995).

[b39] Platt J. Sequential minimal optimization: A fast algorithm for training support vector machine (1999).

[b40] Hall M., Frank E., Holmes G., Pfahringer B., Reutemann P. & Witten I. H. The WEKA data mining software: An update. SIGKDD Explorations 10–18 (2009).

[b41] Kohavi R. A study of cross-validation and bootstrap for accuracy estimation and model selection. the Proceedings of the 14th international joint conference on Artificial intelligence 1137–1143 (1995).

[b42] Andreatta M. & Nielsen M. Gapped sequence alignment using artificial neural networks: application to the MHC class I system. Bioinformatics 32, 511–517, doi: 10.1093/bioinformatics/btv639 (2016). 26515819 PMC6402319

[b43] Peng H., Long F. & Ding C. Feature selection based on mutual information: criteria of max-dependency, max-relevance, and min-redundancy. IEEE Transactions on Pattern Analysis and Machine Intelligence 27, 1226–1238 (2005). 16119262 10.1109/TPAMI.2005.159

[b44] Yucesoy B. . Association of MHC region SNPs with irritant susceptibility in healthcare workers. J Immunotoxicol 1–7, doi: 10.3109/1547691X.2016.1173135 (2016). 27258892 PMC5289286

[b45] Colbert R. A., Tran T. M. & Layh-Schmitt G. HLA-B27 misfolding and ankylosing spondylitis. Mol Immunol 57, 44–51, doi: 10.1016/j.molimm.2013.07.013 (2014). 23993278 PMC3857088

[b46] Reveille J. D. An update on the contribution of the MHC to as susceptibility. Clinical rheumatology 33, 749–757, doi: 10.1007/s10067-014-2662-7 (2014). 24838411 PMC4488903

[b47] Sorrentino R., Bockmann R. A. & Fiorillo M. T. HLA-B27 and antigen presentation: At the crossroads between immune defense and autoimmunity. Molecular Immunology 57, 22–27, doi: 10.1016/j.molimm.2013.06.017 (2014). 23916069

[b48] Carreno B. M. . A dendritic cell vaccine increases the breadth and diversity of melanoma neoantigen-specific T cells. Science 348, 803–808, doi: 10.1126/science.aaa3828 (2015). 25837513 PMC4549796

[b49] Hirayama M. & Nishimura Y. The present status and future prospects of peptide-based cancer vaccines. International immunology, doi: 10.1093/intimm/dxw027 (2016). 27235694

[b50] Bakker A. H. . Conditional MHC class I ligands and peptide exchange technology for the human MHC gene products HLA-A1, -A3, -A11, and -B7. Proc Natl Acad Sci USA 105, 3825–3830, doi: 10.1073/pnas.0709717105 (2008). 18308940 PMC2268811

[b51] Yu Z. . Primary and secondary structure of novel ACE-inhibitory peptides from egg white protein. Food Chem 133, 315–322, doi: 10.1016/j.foodchem.2012.01.032 (2012). 25683401

[b52] Hess J. F., Casselman J. T., Kong A. P. & FitzGerald P. G. Primary sequence, secondary structure, gene structure, and assembly properties suggests that the lens-specific cytoskeletal protein filensin represents a novel class of intermediate filament protein. Exp Eye Res 66, 625–644, doi: 10.1006/exer.1998.0478 (1998). 9628810

[b53] Hao H. F., Li X. S., Gao F. S., Wu W. X. & Xia C. Secondary structure and 3D homology modeling of grass carp (Ctenopharyngodon idellus) major histocompatibility complex class I molecules. Protein Expr Purif 51, 120–125, doi: 10.1016/j.pep.2006.08.003 (2007). 17005417

[b54] Cano P. & Fan B. A geometric and algebraic view of MHC-peptide complexes and their binding properties. BMC Struct Biol 1, 2 (2001). 11472639 10.1186/1472-6807-1-2 PMC35285

[b55] Duke J. L. . Determining performance characteristics of an NGS-based HLA typing method for clinical applications. HLA 87, 141–152, doi: 10.1111/tan.12736 (2016). 26880737

[b56] Franzoni G. . Proteome-wide screening reveals immunodominance in the CD8 T cell response against classical swine fever virus with antigen-specificity dependent on MHC class I haplotype expression. PLoS One 8, e84246, doi: 10.1371/journal.pone.0084246 (2013). 24376799 PMC3871537

[b57] Honegger J. R. . Loss of immune escape mutations during persistent HCV infection in pregnancy enhances replication of vertically transmitted viruses. Nature medicine 19, 1529–1533, doi: 10.1038/nm.3351 (2013). PMC3823809 24162814

[b58] Abrams S. I., Stanziale S. F., Lunin S. D., Zaremba S. & Schlom J. Identification of overlapping epitopes in mutant ras oncogene peptides that activate CD4(+) and CD8(+)T cell responses. European Journal of Immunology 26, 435–443, doi: 10.1002/eji.1830260225 (1996). 8617315

[b59] Carreno B. M. . Cancer immunotherapy. A dendritic cell vaccine increases the breadth and diversity of melanoma neoantigen-specific T cells. Science 348, 803–808, doi: 10.1126/science.aaa3828 (2015). 25837513 PMC4549796

[b60] Wucherpfennig K. W. . Structural basis for major histocompatibility complex (MHC)-linked susceptibility to autoimmunity: charged residues of a single MHC binding pocket confer selective presentation of self-peptides in pemphigus vulgaris. Proc Natl Acad Sci USA 92, 11935–11939 (1995). 8524878 10.1073/pnas.92.25.11935 PMC40518

[b61] Xiao C. Y., Perez L. M. & Russell D. H. Effects of charge states, charge sites and side chain interactions on conformational preferences of a series of model peptide ions. Analyst 14, 6933–6944, doi: 10.1039/c5an00826c (2015). 26081298

[b62] Gairin J. E. & Oldstone M. B. A. Virus and Cytotoxic T-Lymphocytes - Crucial Role of Viral Peptide Secondary Structure in Major Histocompatibility Complex Class-I Interactions. J Virol 67, 2903–2907 (1993). 7682632 10.1128/jvi.67.5.2903-2907.1993 PMC237616

[b63] Khan A. R., Baker B. M., Ghosh P., Biddison W. E. & Wiley D. C. The structure and stability of an HLA-A*0201/octameric tax peptide complex with an empty conserved peptide-N-terminal binding site. Journal of immunology 164, 6398–6405 (2000). 10.4049/jimmunol.164.12.6398 10843695

[b64] Bjorkman P. J. . Structure of the Human Class-I Histocompatibility Antigen, Hla-A2. Nature 329, 506–512, doi: 10.1038/329506a0 (1987). 3309677

[b65] Beringer D. X. . T cell receptor reversed polarity recognition of a self-antigen major histocompatibility complex. Nat Immunol 16, 1153–1161, doi: 10.1038/ni.3271 (2015). 26437244

