# Supplementary material for: Identification of the core regulators of the HLA I-peptide binding process
Source: Sci Rep. 2017 Feb 17;7:42768. doi: 10.1038/srep42768 (PMC5314381; doi:10.1038/srep42768)
Supplement: Supplementary Materials [file srep42768-s1.pdf]

Identification of the core regulators of the HLA I-peptide binding process

Yu-Hang Zhang , Zhihao Xing , Chenglin Liu , ShaoPeng Wang , Tao Huang , Yu-Dong Cai , Xiangyin Kong

Supplementary Material S1 The description of 3360 HLA I-peptide interaction pairs with protein name, peptide and their binding affinity.

Protein name	peptide	Binding affinity
MHC_A_01_01	GTEYRLTLY	high
MHC_A_01_01	SVDSDHLGY	high
MHC_A_01_01	NTDDFPLTL	high
MHC_A_01_01	SAEVVTLWY	high
MHC_A_01_01	WTDLFDNKV	high
MHC_A_01_01	LVDENQSWY	high
MHC_A_01_01	HLDMLRHLY	high
MHC_A_01_01	SVDGFRASY	high
MHC_A_01_01	WSTIWRQLY	high
MHC_A_01_01	FTFWTFANY	high
MHC_A_01_01	YTAVVPLVY	high
MHC_A_01_01	DSDGSFFLY	high
MHC_A_01_01	EVDPIGHLY	high
MHC_A_01_01	GSEDRDLLY	high
MHC_A_01_01	CSEYVKDIY	high
MHC_A_01_01	IVDCLTEMYY	high
MHC_A_02_01	ALWIPDLFM	high
MHC_A_02_01	AMFIGHATA	high
MHC_A_02_01	FLSNGHVTI	high
MHC_A_02_01	FMDGKQACV	high
MHC_A_02_01	FQWHEAMFL	high
MHC_A_02_01	MQDVFTFYV	high
MHC_A_02_01	RTFHIFYYL	high
MHC_A_02_01	SVYDFFVWL	high
MHC_A_02_01	TLKDGDFIL	high
MHC_A_02_01	TLWVDPYEV	high
MHC_A_02_01	VLWKSYPLV	high
MHC_A_02_01	VVPSYIPLV	high
MHC_A_02_01	YTYSGLFCV	high
MHC_A_02_01	FVFDRPLPV	high
MHC_A_02_01	QLFNHTMFI	high
MHC_A_02_01	VLIRRCHYL	high
MHC_A_02_01	FLFDRLTNG	high
MHC_A_02_01	ALWGFFPVL	high

MHC_A_02_01	FLGMESCGI	high
MHC_A_02_01	ILAKFLHWL	high
MHC_A_02_01	GLEAYIQGI	high
MHC_A_02_01	NMVPFFPPV	high
MHC_A_02_01	LMQCWQLLA	high
MHC_A_02_01	KTWGQYWQV	high
MHC_A_02_01	YITDYSNDI	high
MHC_A_02_01	TIWAANAGV	high
MHC_A_02_01	YLYVDKNFI	high
MHC_A_02_01	WLMKNMDPL	high
MHC_A_02_01	KMVELVHFL	high
MHC_A_02_01	LMTGDTYTA	high
MHC_A_02_01	YQGSYGFRL	high
MHC_A_02_01	MLGTHTMEV	high
MHC_A_02_01	FVWLHYYSV	high
MHC_A_02_01	QLFEDNYAL	high
MHC_A_02_01	FLHSGTAKS	high
MHC_A_02_01	GLHCDFACL	high
MHC_A_02_01	GLIYTYSGL	high
MHC_A_02_01	FLLRWEQEI	high
MHC_A_02_01	SLDDYNHLV	high
MHC_A_02_01	RMYGVLPWI	high
MHC_A_02_01	FIDSYICQV	high
MHC_A_02_01	FLCWGPFFL	high
MHC_A_02_01	FMECNLNEL	high
MHC_A_02_01	ILHNGAYSL	high
MHC_A_02_01	QLFNHTMFIL	high
MHC_A_02_01	YTYSGLFCVV	high
MHC_A_02_01	MMQDREDQSI	high
MHC_A_02_01	RMFRWLVLRI	high
MHC_A_02_01	SLYASSPGGV	high
MHC_A_02_01	KLRRGDLPFV	high
MHC_A_02_01	NMDWRSLTQV	high
MHC_A_02_01	YLLSGAGEHL	high
MHC_A_02_01	SLSTFQQMWI	high
MHC_A_02_01	HLSWEWNLSI	high
MHC_A_02_01	KMDSFLDMQL	high
MHC_A_02_01	VLDQLRCNGV	high
MHC_A_02_01	ALRTDYNASV	high
MHC_A_02_01	VLYRYGSFSV	high
MHC_A_02_01	LLLDVTPLSL	high
MHC_A_02_01	TLRNTNPNFV	high
MHC_A_02_01	FMDGKQACVL	high

MHC_A_02_01	KMVELVHFLL	high
MHC_A_02_01	YLWWVNNQSL	high
MHC_A_02_01	LLFFWLDRSV	high
MHC_A_02_01	KLLEGEEERL	high
MHC_A_02_01	WLLGAAMVGA	high
MHC_A_02_01	KMNPPKFSKV	high
MHC_A_02_01	GIMIGVLVGV	high
MHC_A_02_01	GQYGEVYEGV	high
MHC_A_02_01	LVFGIELMEV	high
MHC_A_02_01	ALDGGNKHFL	high
MHC_A_02_01	LLMWITQCFL	high
MHC_A_02_01	YLAEADLSYT	high
MHC_A_02_01	YMIMVKCWMI	high
MHC_A_02_01	YLQLVFGIEV	high
MHC_A_02_01	KMVELVHFLLL	high
MHC_A_02_01	FLLPILSQIYT	high
MHC_A_02_01	QLVFGIELMEV	high
MHC_A_02_01	FMIAATYNFAV	high
MHC_A_02_01	SLLERGQQLGV	high
MHC_A_02_01	LLPENNVLSPL	high
MHC_A_02_01	IMPKAGLLIIV	high
MHC_A_02_02	FLSNGHVTI	high
MHC_A_02_02	FVFDRPLPV	high
MHC_A_02_02	ILAKFLHWL	high
MHC_A_02_02	QLFNHTMFI	high
MHC_A_02_02	WLMKNMDPL	high
MHC_A_02_02	YLYVDKNFI	high
MHC_A_02_02	FMECNLNEL	high
MHC_A_02_02	YTYSGLFCV	high
MHC_A_02_02	HLREYQDLL	high
MHC_A_02_02	RTFHIFYYL	high
MHC_A_02_02	FMDGKQACV	high
MHC_A_02_02	GLENGLNYI	high
MHC_A_02_02	GLIYTYSGL	high
MHC_A_02_02	GMGQKDSYV	high
MHC_A_02_02	SMHFYGWSL	high
MHC_A_02_02	KMFCQLAKT	high
MHC_A_02_02	CQLAKTCPV	high
MHC_A_02_02	QLFNHTMFIL	high
MHC_A_02_02	YTYSGLFCVV	high
MHC_A_02_02	NLKERYYSGL	high
MHC_A_02_02	RMFRWLVLRI	high
MHC_A_02_02	SLYASSPGGV	high

MHC_A_02_02	QLKDKADFCI	high
MHC_A_02_02	SLSTFQQMWI	high
MHC_A_02_02	ALRTDYNASV	high
MHC_A_02_02	VLKEGSEYRV	high
MHC_A_02_02	YLLSGAGEHL	high
MHC_A_02_02	FMECNLNELV	high
MHC_A_02_02	ILHVDNHIGI	high
MHC_A_02_02	HLSWEWNLSI	high
MHC_A_02_02	KLRRGDLPFV	high
MHC_A_02_02	VLQRNCAAYL	high
MHC_A_02_02	LVFGIELMEV	high
MHC_A_02_02	AMYVAIQAVL	high
MHC_A_02_02	WLVLRINKAL	high
MHC_A_02_02	MMQDREDQSI	high
MHC_A_02_02	FMDGKQACVL	high
MHC_A_02_02	HMSEFMECNL	high
MHC_A_02_02	KLLEGEEERL	high
MHC_A_02_02	LLPENNVLSPL	high
MHC_A_02_02	QMNSLRAEDTA	high
MHC_A_02_03	FVFDRPLPV	high
MHC_A_02_03	FMECNLNEL	high
MHC_A_02_03	ILAKFLHWL	high
MHC_A_02_03	FLHSGTAKS	high
MHC_A_02_03	YTYSGLFCV	high
MHC_A_02_03	QLFNHTMFI	high
MHC_A_02_03	FLSNGHVTI	high
MHC_A_02_03	YLYVDKNFI	high
MHC_A_02_03	GLIYTYSGL	high
MHC_A_02_03	FLGMESCGI	high
MHC_A_02_03	HLYQGCQVV	high
MHC_A_02_03	WLMKNMDPL	high
MHC_A_02_03	KMFCQLAKT	high
MHC_A_02_03	ALNKMFCQL	high
MHC_A_02_03	YLQMNSLRA	high
MHC_A_02_03	FMDGKQACV	high
MHC_A_02_03	YTYSGLFCVV	high
MHC_A_02_03	GLKSKTHAVL	high
MHC_A_02_03	SLYASSPGGV	high
MHC_A_02_03	RMFRWLVLRI	high
MHC_A_02_03	SLSTFQQMWI	high
MHC_A_02_03	LVFGIELMEV	high
MHC_A_02_03	QLFNHTMFIL	high
MHC_A_02_03	KLRRGDLPFV	high

MHC_A_02_03	ALRTDYNASV	high
MHC_A_02_03	ILHVDNHIGI	high
MHC_A_02_03	VMAGVGSPYV	high
MHC_A_02_03	SLPLPNFSSL	high
MHC_A_02_03	NMDWRSLTQV	high
MHC_A_02_03	YLWWVNNQSL	high
MHC_A_02_03	LLQSKNAGAV	high
MHC_A_02_03	TLRNTNPNFV	high
MHC_A_02_03	RLPGPSDTPI	high
MHC_A_02_03	KMNPPKFSKV	high
MHC_A_02_03	LLPENNVLSPL	high
MHC_A_02_03	IMPKAGLLIIV	high
MHC_A_02_06	YTYSGLFCV	high
MHC_A_02_06	RTFHIFYYL	high
MHC_A_02_06	FVFDRPLPV	high
MHC_A_02_06	WLMKNMDPL	high
MHC_A_02_06	FMDGKQACV	high
MHC_A_02_06	LQIRGRERF	high
MHC_A_02_06	FLSNGHVTI	high
MHC_A_02_06	QLFNHTMFI	high
MHC_A_02_06	KTCPVQLWV	high
MHC_A_02_06	FLGMESCGI	high
MHC_A_02_06	ILAKFLHWL	high
MHC_A_02_06	TIWAANAGV	high
MHC_A_02_06	YLYVDKNFI	high
MHC_A_02_06	NMDWRSLTQV	high
MHC_A_02_06	YTYSGLFCVV	high
MHC_A_02_06	KLRRGDLPFV	high
MHC_A_02_06	YLLSGAGEHL	high
MHC_A_02_06	LVFGIELMEV	high
MHC_A_02_06	QLFNHTMFIL	high
MHC_A_02_06	LLPENNVLSPL	high
MHC_A_02_11	AMFIGHATA	high
MHC_A_02_11	GLEAYIQGI	high
MHC_A_02_12	MQDVFTFYV	high
MHC_A_02_12	FQWHEAMFL	high
MHC_A_02_12	LMQCWQLLA	high
MHC_A_02_12	GLEAYIQGI	high
MHC_A_02_12	YITDYSNDI	high
MHC_A_02_12	VLWKSYPLV	high
MHC_A_02_12	TLKDGDFIL	high
MHC_A_02_12	VLIRRCHYL	high
MHC_A_02_12	AMFIGHATA	high

MHC_A_03_01	HLFGYSWYK	high
MHC_A_03_01	RVYSPYNHR	high
MHC_A_03_01	LIYRRRLMK	high
MHC_A_03_01	ALDGTFQRK	high
MHC_A_03_01	KIRKYTMRR	high
MHC_A_03_01	ILWKDIFHK	high
MHC_A_03_01	SIFEQWLRR	high
MHC_A_03_01	YMVPFIPLYR	high
MHC_A_03_01	KVLRENTSPK	high
MHC_A_03_01	IALNFPGSQK	high
MHC_A_03_01	SLIYRRRLMK	high
MHC_A_03_01	ITHTGEKPYK	high
MHC_A_03_01	AALDGTFQRK	high
MHC_A_11_01	HLFGYSWYK	high
MHC_A_11_01	AALDGTFQR	high
MHC_A_11_01	SIFEQWLRR	high
MHC_A_11_01	TTINYTLWR	high
MHC_A_11_01	TTINFTRQR	high
MHC_A_11_01	QTSAGHFPR	high
MHC_A_11_01	RVYNNTARY	high
MHC_A_11_01	ALDGTFQRK	high
MHC_A_11_01	GTQCALTRR	high
MHC_A_11_01	AALDGTFQRK	high
MHC_A_11_01	YMVPFIPLYR	high
MHC_A_11_01	SSSHWLRLPR	high
MHC_A_23_01	YYADSVKGRF	high
MHC_A_23_01	LSCAASGFTF	high
MHC_A_24_02	RWGLLLALL	high
MHC_A_24_02	NWQYFFPVI	high
MHC_A_24_02	HYNAFQWAI	high
MHC_A_24_02	AFLPWHRLF	high
MHC_A_24_02	LYSACFWWL	high
MHC_A_24_02	VYMIMVKCW	high
MHC_A_24_02	SYGVTVWEL	high
MHC_A_24_02	QYSWFVNGTF	high
MHC_A_24_02	YYADSVKGRF	high
MHC_A_24_02	SYMMDDLELI	high
MHC_A_24_02	YYRPGVNLSL	high
MHC_A_25_01	YTYPCIPEY	high
MHC_A_25_01	ETFNTPAMY	high
MHC_A_25_01	YTSDYFISY	high
MHC_A_26_01	ETFGFEIQSY	high
MHC_A_29_02	AFFAERLYY	high

MHC_A_29_02	FFGETSHNY	high
MHC_A_29_02	FTFWTFANY	high
MHC_A_29_02	GYLHDFLKY	high
MHC_A_29_02	MLYPRVWPY	high
MHC_A_29_02	SILPISWAY	high
MHC_A_29_02	SWNNHSYLY	high
MHC_A_29_02	TFMDHVLRY	high
MHC_A_29_02	YTSDYFISY	high
MHC_A_29_02	YTYPCIPEY	high
MHC_A_29_02	YYSNKAYQY	high
MHC_A_29_02	YYWPRPRRY	high
MHC_A_29_02	SVDGFRASY	high
MHC_A_29_02	YTAVVPLVY	high
MHC_A_29_02	ETFNTPAMY	high
MHC_A_29_02	IIYVGCGER	high
MHC_A_29_02	FAFVTDNTY	high
MHC_A_29_02	YFEYIEENKY	high
MHC_A_29_02	YYRYPTGESY	high
MHC_A_29_02	IVDCLTEMYY	high
MHC_A_29_02	YMIDPSGVSY	high
MHC_A_29_02	YYADSVKGRF	high
MHC_A_29_02	MVRVLTVIKEY	high
MHC_A_29_02	TFVPIAWAAAY	high
MHC_A_29_02	VALFSSCPVAY	high
MHC_A_30_01	RVYNNTARY	high
MHC_A_30_02	CAASGFTFSSY	high
MHC_A_31_01	YTKVVHYRK	high
MHC_A_31_01	HLFGYSWYK	high
MHC_A_31_01	ISYPPLHER	high
MHC_A_31_01	TTINFTRQR	high
MHC_A_31_01	AALDGTFQR	high
MHC_A_31_01	SVKGRFTISR	high
MHC_A_32_07	SILPISWAY	high
MHC_A_32_15	SILPISWAY	high
MHC_A_33_01	YTKVVHYRK	high
MHC_A_33_01	HLFGYSWYK	high
MHC_A_33_01	TTINFTRQR	high
MHC_A_68_01	AALDGTFQR	high
MHC_A_68_01	DTAVYYCAR	high
MHC_A_68_01	HLFGYSWYK	high
MHC_A_68_01	SSYGMHWVR	high
MHC_A_68_01	TTINYTLWR	high
MHC_A_68_01	YTKVVHYRK	high

MHC_A_68_01	TTINFTRQR	high
MHC_A_68_01	EVYEGVWKK	high
MHC_A_68_01	FTISRDNSK	high
MHC_A_68_01	AVIWYDGSNK	high
MHC_A_68_01	FSSYGMHWVR	high
MHC_A_68_01	SVKGRFTISR	high
MHC_A_68_01	ETFGFEIQSY	high
MHC_A_68_01	VAALDGTFQR	high
MHC_A_68_01	TLYLQMNSLR	high
MHC_A_68_02	FVFDRPLPV	high
MHC_A_68_02	EMPPHIYAI	high
MHC_A_68_02	WLMKNMDPL	high
MHC_A_68_02	YTYSGLFCV	high
MHC_A_68_02	QLFNHTMFI	high
MHC_A_68_02	TIWAANAGV	high
MHC_A_68_02	GLIYTYSGL	high
MHC_A_68_02	YTYSGLFCVV	high
MHC_A_68_02	SLPLPNFSSL	high
MHC_A_68_02	LVFGIELMEV	high
MHC_A_68_02	TMEAMRIMGI	high
MHC_A_80_01	SILPISWAY	high
MHC_A_80_01	YTYPCIPEY	high
MHC_A_80_01	AFFAERLYY	high
MHC_B_07_02	RPNRQLGSM	high
MHC_B_07_02	FAAPHRGVA	high
MHC_B_07_02	KVRDRNFQL	high
MHC_B_07_02	GVRQFSGWM	high
MHC_B_07_02	HPQKVTKFM	high
MHC_B_07_02	CPIGENSPL	high
MHC_B_07_02	GPGAGSLQPL	high
MHC_B_08_01	SMHFYGWSL	high
MHC_B_08_01	SNKRHRPIG	high
MHC_B_08_01	TLYLQMNSL	high
MHC_B_15_01	YLGEFSITY	high
MHC_B_15_01	VVAPITTGY	high
MHC_B_15_01	GQRKPATSY	high
MHC_B_15_01	ILGPPGSVY	high
MHC_B_15_01	YIITEFMTY	high
MHC_B_15_01	YGFSDPLTF	high
MHC_B_15_01	SIKSDVWAF	high
MHC_B_15_01	LQIRGRERF	high
MHC_B_15_01	SQFGGGSQY	high
MHC_B_15_01	IQPGRGFVLY	high

MHC_B_15_01	VLKPGMVVTF	high
MHC_B_15_01	YMIDPSGVSY	high
MHC_B_15_01	GQRKGAGSVF	high
MHC_B_15_02	FAFVTDNTY	high
MHC_B_15_02	FIKDGSSTY	high
MHC_B_15_02	SIRDGVRAY	high
MHC_B_15_03	IKADHVSTY	high
MHC_B_15_03	SQFGGGSQY	high
MHC_B_15_03	GQRKGAGSVF	high
MHC_B_15_17	RVYNNTARY	high
MHC_B_18_01	TEAEKWPFF	high
MHC_B_27_05	RRYNIIPVL	high
MHC_B_27_05	FRYNGLIHR	high
MHC_B_27_05	LRFPGQLNA	high
MHC_B_27_05	SRTPYHVNL	high
MHC_B_27_05	SRHHAFCFR	high
MHC_B_27_05	LRLSCAASGF	high
MHC_B_27_05	NRFAGFGIGL	high
MHC_B_27_05	SRLWPKIQGL	high
MHC_B_27_20	FRMLAWHVL	high
MHC_B_27_20	RRHWGGNVL	high
MHC_B_27_20	FEYGGFPPA	high
MHC_B_35_01	FPHTELANL	high
MHC_B_35_01	MPIEPGDIGY	high
MHC_B_35_01	IVDCLTEMYY	high
MHC_B_35_01	VALFSSCPVAY	high
MHC_B_35_01	MVRVLTVIKEY	high
MHC_B_35_01	LPSCPTNFCIF	high
MHC_B_40_02	TEAEKWPFF	high
MHC_B_44_02	EEGNLLDSYF	high
MHC_B_44_02	AELLNIPFLY	high
MHC_B_44_03	AELLNIPFLY	high
MHC_B_44_03	EEGNLLDSYF	high
MHC_B_46_01	FAFVTDNTY	high
MHC_B_46_01	YTFCGTIEY	high
MHC_B_46_01	YTAVVPLVY	high
MHC_B_46_01	YMIDPSGVSY	high
MHC_B_51_01	MPREDAHFI	high
MHC_B_51_01	LPKEACMEI	high
MHC_B_51_01	CPLERFAEL	high
MHC_B_51_01	LPFRNCPRF	high
MHC_B_51_01	FPPEGVSIW	high
MHC_B_51_01	TPMFNDINI	high

MHC_B_51_01	LPSIPVHPI	high
MHC_B_52_01	YGFSDPLTF	high
MHC_B_53_01	MPREDAHFI	high
MHC_B_53_01	FPPEGVSIW	high
MHC_B_54_01	DPIFLLHHA	high
MHC_B_54_01	LPSIPVHPI	high
MHC_B_57_01	KAGQVVTIW	high
MHC_B_57_01	ITSQDVLHSW	high
MHC_B_57_01	ISDSNPFLTQW	high
MHC_B_58_01	KAGQVVTIW	high
MHC_B_58_01	RVYNNTARY	high
MHC_B_58_01	YTAVVPLVY	high
MHC_B_58_01	GTVLKTSSW	high
MHC_B_58_01	ITSQDVLHSW	high
MHC_B_58_01	ASYSGKAADVW	high
MHC_B_58_01	ISDSNPFLTQW	high
MHC_B_73_01	FEYGGFPPA	high
MHC_B_73_01	YEHYFVFAA	high
MHC_C_03_03	VALFSSCPV	high
MHC_C_04_01	YFDPANGKF	high
MHC_C_04_01	WYDALCVLL	high
MHC_C_06_02	FRMLAWHVL	high
MHC_C_06_02	NYIDKVRFL	high
MHC_C_06_02	YYFSYPLFV	high
MHC_C_06_02	YYWPRPRRY	high
MHC_C_07_01	NYIDKVRFL	high
MHC_C_07_01	FRMLAWHVL	high
MHC_C_07_01	YYFSYPLFV	high
MHC_C_07_01	YYWPRPRRY	high
MHC_C_07_02	FRHSVVVPY	high
MHC_C_07_02	YYWPRPRRY	high
MHC_C_07_02	HYNAFQWAI	high
MHC_C_07_02	FRMLAWHVL	high
MHC_C_12_03	FAAPHRGVA	high
MHC_C_12_03	FIFPASKVY	high
MHC_C_12_03	NTFKFGVIY	high
MHC_C_12_03	YQFTGIKKY	high
MHC_C_12_03	YTFCGTIEY	high
MHC_C_12_03	YTYPCIPEY	high
MHC_C_14_02	RYFSVTRPL	high
MHC_C_14_02	TFMDHVLRY	high
MHC_C_14_02	YYFSYPLFV	high
MHC_C_14_02	YYRYPTGESY	high

MHC_C_15_02	YSLEYFQFV	high
MHC_C_15_02	KTWGQYWQV	high
MHC_A_01_01	RGPDAFRF	low
MHC_A_01_01	YTSDYFISY	low
MHC_A_01_01	KSDLQPPNY	low
MHC_A_01_01	RTDNGGWAH	low
MHC_A_01_01	YLDDPDLKY	low
MHC_A_01_01	IVDCLTEMY	low
MHC_A_01_01	EADPTGHSY	low
MHC_A_01_01	GSDCTTIHY	low
MHC_A_01_01	YVDHYYRDY	low
MHC_A_01_01	IADMGHLKY	low
MHC_A_01_01	ATDFKFAMY	low
MHC_A_01_01	STDHIPILY	low
MHC_A_01_01	GTQLFEDNY	low
MHC_A_01_01	MIEPRTLQY	low
MHC_A_01_01	YTYPCIPEY	low
MHC_A_01_01	CSDDGFWSK	low
MHC_A_01_01	RAEDTAVYY	low
MHC_A_01_01	NTFKFGVIY	low
MHC_A_01_01	KCDICTDEY	low
MHC_A_01_01	IYDFYYLDY	low
MHC_A_01_01	LQDDFDFNY	low
MHC_A_01_01	MLYPRVWPY	low
MHC_A_01_01	ETFNTPAMY	low
MHC_A_01_01	TFMDHVLRY	low
MHC_A_01_01	YLDFGGPEG	low
MHC_A_01_01	MCFHQHLMY	low
MHC_A_01_01	YFDPANGKF	low
MHC_A_01_01	SWNNHSYLY	low
MHC_A_01_01	ILGPPGSVY	low
MHC_A_01_01	DTAVYYCAR	low
MHC_A_01_01	SQFGGGSQY	low
MHC_A_01_01	TLYLQMNSL	low
MHC_A_01_01	VPDSDPARY	low
MHC_A_01_01	LRAEDTAVY	low
MHC_A_01_01	WVDSTPPPG	low
MHC_A_01_01	FTFSSYGMH	low
MHC_A_01_01	AFFAERLYY	low
MHC_A_01_01	CHKGWGVSV	low
MHC_A_01_01	DFDGTPRLY	low
MHC_A_01_01	DPSGAYFAW	low
MHC_A_01_01	EPEPHILLF	low

MHC_A_01_01	FFGETSHNY	low
MHC_A_01_01	FHMDPSGTF	low
MHC_A_01_01	FPEHIFPAL	low
MHC_A_01_01	FPHTELANL	low
MHC_A_01_01	FQILHDRFF	low
MHC_A_01_01	GHLENNPAL	low
MHC_A_01_01	GLENGLNYI	low
MHC_A_01_01	GRNSFEVRV	low
MHC_A_01_01	GRPNCFQIV	low
MHC_A_01_01	GYLHDFLKY	low
MHC_A_01_01	IPYHIVNIV	low
MHC_A_01_01	LPRWPPPQL	low
MHC_A_01_01	LVYNHCEHG	low
MHC_A_01_01	NPNSPSITY	low
MHC_A_01_01	NQRETTVVW	low
MHC_A_01_01	NRRFVNVVP	low
MHC_A_01_01	NYIDKVRFL	low
MHC_A_01_01	RPRHQGVMV	low
MHC_A_01_01	RRFFPYYVY	low
MHC_A_01_01	RRHWGGNVL	low
MHC_A_01_01	RVYNNTARY	low
MHC_A_01_01	RYFSVTRPL	low
MHC_A_01_01	SMIYFFHHY	low
MHC_A_01_01	SPRYIFTML	low
MHC_A_01_01	SRDKTIIMW	low
MHC_A_01_01	SSKMFNYFK	low
MHC_A_01_01	TEAEKWPFF	low
MHC_A_01_01	VGYVDDTQF	low
MHC_A_01_01	WVGRASDPD	low
MHC_A_01_01	YGDTEAICR	low
MHC_A_01_01	YHEDIHTYL	low
MHC_A_01_01	YLGSWATGK	low
MHC_A_01_01	YPAEITLTW	low
MHC_A_01_01	YYSNKAYQY	low
MHC_A_01_01	YYWPRPRRY	low
MHC_A_01_01	YGFSDPLTF	low
MHC_A_01_01	FRYNGLIHR	low
MHC_A_01_01	AGVWSQDKW	low
MHC_A_01_01	FTISRDNSK	low
MHC_A_01_01	SSYGMHWVR	low
MHC_A_01_01	VRQAPGKGL	low
MHC_A_01_01	RGVEKPPHL	low
MHC_A_01_01	SGKLKVPEW	low

MHC_A_01_01	ETFGFEIQSY	low
MHC_A_01_01	LRAEDTAVYY	low
MHC_A_01_01	SLRAEDTAVY	low
MHC_A_01_01	LRLSCAASGF	low
MHC_A_01_01	YFEYIEENKY	low
MHC_A_01_01	GLEWVAVIWY	low
MHC_A_01_01	TLYLQMNSLR	low
MHC_A_01_01	ELFARSSDPR	low
MHC_A_01_01	SRDSRGKPGY	low
MHC_A_01_01	SYMMDDLELI	low
MHC_A_01_01	YYRYPTGESY	low
MHC_A_01_01	LSCAASGFTF	low
MHC_A_01_01	KGFTDADNTW	low
MHC_A_01_01	IWYDGSNKYY	low
MHC_A_01_01	KGFSEEHNTW	low
MHC_A_01_01	AVIWYDGSNK	low
MHC_A_01_01	FSSYGMHWVR	low
MHC_A_01_01	GQRKGAGSVF	low
MHC_A_01_01	ISRDNSKNTL	low
MHC_A_01_01	RLPGPSDTPI	low
MHC_A_01_01	SGGPKYEYRW	low
MHC_A_01_01	SVKGRFTISR	low
MHC_A_01_01	WVRQAPGKGL	low
MHC_A_01_01	YYADSVKGRF	low
MHC_A_01_01	SLRAEDTAVYY	low
MHC_A_01_01	NSLRAEDTAVY	low
MHC_A_01_01	CAASGFTFSSY	low
MHC_A_01_01	SLRLSCAASGF	low
MHC_A_01_01	FLLPILSQIYT	low
MHC_A_01_01	GSSDFQVHFLK	low
MHC_A_01_01	LEYFQFVKKLL	low
MHC_A_01_01	LPSCPTNFCIF	low
MHC_A_01_01	MIKYCLLKILK	low
MHC_A_01_01	MVRVLTVIKEY	low
MHC_A_01_01	RMILPMSRAFR	low
MHC_A_01_01	SLLERGQQLGV	low
MHC_A_01_01	TFVPIAWAAAY	low
MHC_A_01_01	VALFSSCPVAY	low
MHC_A_01_01	YFYYNAFHWAI	low
MHC_A_01_01	YSLEYFQFVKK	low
MHC_A_01_01	EVQLVESGGGL	low
MHC_A_01_01	RLPGPSDTPIL	low
MHC_A_01_01	RLSCAASGFTF	low

MHC_A_02_01	HLYQGCQV	low
MHC_A_02_01	HLREYQDLL	low
MHC_A_02_01	YSLEYFQFV	low
MHC_A_02_01	YMNGTMSQV	low
MHC_A_02_01	LMWITQCFL	low
MHC_A_02_01	ALCRWGLLL	low
MHC_A_02_01	RIWSWLLGA	low
MHC_A_02_01	YVITTQHWL	low
MHC_A_02_01	FLPWHRLFL	low
MHC_A_02_01	RTFSPTYGL	low
MHC_A_02_01	YYFSYPLFV	low
MHC_A_02_01	HLYQGCQVV	low
MHC_A_02_01	CLLQSLQQI	low
MHC_A_02_01	VTWHRYHLL	low
MHC_A_02_01	WITQCFLPV	low
MHC_A_02_01	RLMTGDTYT	low
MHC_A_02_01	LLTFWNPPT	low
MHC_A_02_01	YIFATCLGL	low
MHC_A_02_01	GMGQKDSYV	low
MHC_A_02_01	SLFNWLWYE	low
MHC_A_02_01	CQLAKTCPV	low
MHC_A_02_01	QLMPYGCLL	low
MHC_A_02_01	YMIMVKCWM	low
MHC_A_02_01	SMHFYGWSL	low
MHC_A_02_01	ALIHHNTHL	low
MHC_A_02_01	FLWGPRALI	low
MHC_A_02_01	SLLMWITQC	low
MHC_A_02_01	EMPPHIYAI	low
MHC_A_02_01	KMFCQLAKT	low
MHC_A_02_01	GLACHQLCA	low
MHC_A_02_01	YLLPAIVHI	low
MHC_A_02_01	GLENGLNYI	low
MHC_A_02_01	NIHSEMYSL	low
MHC_A_02_01	FRYNGLIHR	low
MHC_A_02_01	KTCPVQLWV	low
MHC_A_02_01	ALNKMFCQL	low
MHC_A_02_01	GMFRTVGQL	low
MHC_A_02_01	ALWGPDPAA	low
MHC_A_02_01	YLQMNSLRA	low
MHC_A_02_01	FVTWHRYHL	low
MHC_A_02_01	CIRHEMTPV	low
MHC_A_02_01	ALPYWNFAT	low
MHC_A_02_01	SLHVGTQCA	low

MHC_A_02_01	YLDFGGPEG	low
MHC_A_02_01	NMDPLNDNI	low
MHC_A_02_01	DLTDYLMKI	low
MHC_A_02_01	YLDDPDLKY	low
MHC_A_02_01	LLSCLGCKI	low
MHC_A_02_01	AAAFVNQHL	low
MHC_A_02_01	MTGDTYTAH	low
MHC_A_02_01	SLYQLENYC	low
MHC_A_02_01	KLGGGQYGE	low
MHC_A_02_01	WMKRFNPPA	low
MHC_A_02_01	TIKCSNLCT	low
MHC_A_02_01	HASPTLFNA	low
MHC_A_02_01	QHLCGSHLV	low
MHC_A_02_01	ATGRNECDV	low
MHC_A_02_01	CLERDLQRL	low
MHC_A_02_01	GAQGQFPRV	low
MHC_A_02_01	ITTQHWLGL	low
MHC_A_02_01	NCERKKPPV	low
MHC_A_02_01	NCSVYDFFV	low
MHC_A_02_01	QGRGQCTEV	low
MHC_A_02_01	QMGRNSMKL	low
MHC_A_02_01	TLKDIRDCL	low
MHC_A_02_01	TTQHWLGLL	low
MHC_A_02_01	YTPLMETHL	low
MHC_A_02_01	FHMDPSGTF	low
MHC_A_02_01	DLQPCIDLI	low
MHC_A_02_01	YTAVVPLVY	low
MHC_A_02_01	YLGSWATGK	low
MHC_A_02_01	KVPRNQDWL	low
MHC_A_02_01	ATDFKFAMY	low
MHC_A_02_01	CHKGWGVSV	low
MHC_A_02_01	CSDDGFWSK	low
MHC_A_02_01	CSEYVKDIY	low
MHC_A_02_01	DFDGTPRLY	low
MHC_A_02_01	DIKPDNILL	low
MHC_A_02_01	DPSGAYFAW	low
MHC_A_02_01	DSDGSFFLY	low
MHC_A_02_01	EADPTGHSY	low
MHC_A_02_01	EPEPHILLF	low
MHC_A_02_01	FAAPHRGVA	low
MHC_A_02_01	FPEHIFPAL	low
MHC_A_02_01	FPHTELANL	low
MHC_A_02_01	FQILHDRFF	low

MHC_A_02_01	FWAWSVLRV	low
MHC_A_02_01	GHLENNPAL	low
MHC_A_02_01	GRNSFEVRV	low
MHC_A_02_01	GRPNCFQIV	low
MHC_A_02_01	GSEDRDLLY	low
MHC_A_02_01	GTEYRLTLY	low
MHC_A_02_01	GVRQFSGWM	low
MHC_A_02_01	IADMGHLKY	low
MHC_A_02_01	IIYVGCGER	low
MHC_A_02_01	ILGPPGSVY	low
MHC_A_02_01	IPYHIVNIV	low
MHC_A_02_01	IVDCLTEMY	low
MHC_A_02_01	IYDFYYLDY	low
MHC_A_02_01	KCDICTDEY	low
MHC_A_02_01	KSDLQPPNY	low
MHC_A_02_01	KVRDRNFQL	low
MHC_A_02_01	LPRWPPPQL	low
MHC_A_02_01	LQDDFDFNY	low
MHC_A_02_01	LVDENQSWY	low
MHC_A_02_01	LVYNHCEHG	low
MHC_A_02_01	MCFHQHLMY	low
MHC_A_02_01	MIEPRTLQY	low
MHC_A_02_01	NPNSPSITY	low
MHC_A_02_01	NQRETTVVW	low
MHC_A_02_01	NRRFVNVVP	low
MHC_A_02_01	RPNRQLGSM	low
MHC_A_02_01	RPRHQGVMV	low
MHC_A_02_01	RRFFPYYVY	low
MHC_A_02_01	RRHWGGNVL	low
MHC_A_02_01	RTDNGGWAH	low
MHC_A_02_01	RVYNNTARY	low
MHC_A_02_01	RYFSVTRPL	low
MHC_A_02_01	SAEVVTLWY	low
MHC_A_02_01	SILPISWAY	low
MHC_A_02_01	SPRYIFTML	low
MHC_A_02_01	SQFGGGSQY	low
MHC_A_02_01	SRDKTIIMW	low
MHC_A_02_01	SSKMFNYFK	low
MHC_A_02_01	STDHIPILY	low
MHC_A_02_01	SVDSDHLGY	low
MHC_A_02_01	TEAEKWPFF	low
MHC_A_02_01	VGYVDDTQF	low
MHC_A_02_01	WSTIWRQLY	low

MHC_A_02_01	WTDLFDNKV	low
MHC_A_02_01	WVGRASDPD	low
MHC_A_02_01	YGDTEAICR	low
MHC_A_02_01	YHEDIHTYL	low
MHC_A_02_01	YPAEITLTW	low
MHC_A_02_01	YSDNEMLTH	low
MHC_A_02_01	YTFCGTIEY	low
MHC_A_02_01	YTSDYFISY	low
MHC_A_02_01	YTYPCIPEY	low
MHC_A_02_01	YVDHYYRDY	low
MHC_A_02_01	YFDPANGKF	low
MHC_A_02_01	DLFGIWSKV	low
MHC_A_02_01	ETFNTPAMY	low
MHC_A_02_01	VVHFFKNIV	low
MHC_A_02_01	WLGHPDKFV	low
MHC_A_02_01	EVDPIGHLY	low
MHC_A_02_01	FAFVTDNTY	low
MHC_A_02_01	FIKDGSSTY	low
MHC_A_02_01	PIGIGVQGL	low
MHC_A_02_01	SIRDGVRAY	low
MHC_A_02_01	ALYLVCGER	low
MHC_A_02_01	AMLGTHTMEV	low
MHC_A_02_01	RLFTKVKPLL	low
MHC_A_02_01	GMPEGDLVYV	low
MHC_A_02_01	KVAELVHFLL	low
MHC_A_02_01	KLRNWQWWRL	low
MHC_A_02_01	FMECNLNELV	low
MHC_A_02_01	SLPLPNFSSL	low
MHC_A_02_01	VLQRNCAAYL	low
MHC_A_02_01	LLGICLTSTV	low
MHC_A_02_01	YLDSGIHSGA	low
MHC_A_02_01	TMEAMRIMGI	low
MHC_A_02_01	SYMMDDLELI	low
MHC_A_02_01	RLMTGDTYTA	low
MHC_A_02_01	ALCRWGLLLA	low
MHC_A_02_01	FLRNQPLTFA	low
MHC_A_02_01	RQASIELPSM	low
MHC_A_02_01	ILHVDNHIGI	low
MHC_A_02_01	FLPWHRLFLL	low
MHC_A_02_01	VLYGPDAPTI	low
MHC_A_02_01	VMAGVGSPYV	low
MHC_A_02_01	RLDCWRGGQV	low
MHC_A_02_01	NMKEEMARHL	low

MHC_A_02_01	SLHNLVHSFL	low
MHC_A_02_01	AQASIELPSM	low
MHC_A_02_01	RILHNGAYSL	low
MHC_A_02_01	RTYSGPMNKV	low
MHC_A_02_01	NLYRIGQSKV	low
MHC_A_02_01	AMYVAIQAVL	low
MHC_A_02_01	QLFEDNYALA	low
MHC_A_02_01	ILDLISESPI	low
MHC_A_02_01	TLKYPIEHGI	low
MHC_A_02_01	VLKEGSEYRV	low
MHC_A_02_01	YMATQISSAM	low
MHC_A_02_01	ALWGPDPAAA	low
MHC_A_02_01	VLPSPACQLV	low
MHC_A_02_01	KVADFGLSRL	low
MHC_A_02_01	HMSEFMECNL	low
MHC_A_02_01	YMIDPSGVSY	low
MHC_A_02_01	GLDLQPCIDL	low
MHC_A_02_01	VLGEAWRDQV	low
MHC_A_02_01	FACSAVPVYI	low
MHC_A_02_01	PMLNPFIYSL	low
MHC_A_02_01	QLKDKADFCI	low
MHC_A_02_01	ELPDGQVITI	low
MHC_A_02_01	WLVLRINKAL	low
MHC_A_02_01	KTWGQYWQVL	low
MHC_A_02_01	GLSQRHEEKV	low
MHC_A_02_01	DLTDYLMKIL	low
MHC_A_02_01	ELNDRLAVYI	low
MHC_A_02_01	WLLVFACSAV	low
MHC_A_02_01	SHLVEALYLV	low
MHC_A_02_01	RLPGPSDTPI	low
MHC_A_02_01	ELRLLIHQSL	low
MHC_A_02_01	LLAFLQYRRL	low
MHC_A_02_01	HLCGSHLVEA	low
MHC_A_02_01	KLGGGQYGEV	low
MHC_A_02_01	LLALWGPDPA	low
MHC_A_02_01	TITDQVPFSV	low
MHC_A_02_01	EAAGIGILTV	low
MHC_A_02_01	QLGYSYAIDL	low
MHC_A_02_01	LAPPQHLIRV	low
MHC_A_02_01	NLPIYSEEIV	low
MHC_A_02_01	GLHCDFACLM	low
MHC_A_02_01	MALWMRLLPL	low
MHC_A_02_01	ELRENTQTTI	low

MHC_A_02_01	PLPNFSSLNL	low
MHC_A_02_01	KLKNKHEAMI	low
MHC_A_02_01	WGPDPAAAFV	low
MHC_A_02_01	ELTCLNEASV	low
MHC_A_02_01	LLNVKMALDI	low
MHC_A_02_01	DLNSFEQLCI	low
MHC_A_02_01	PLSPTRLSRL	low
MHC_A_02_01	FLPRHRDTGI	low
MHC_A_02_01	LLRDAGLVKM	low
MHC_A_02_01	LLQSKNAGAV	low
MHC_A_02_01	AAGIGILTVI	low
MHC_A_02_01	NLKERYYSGL	low
MHC_A_02_01	RLSCAASGFT	low
MHC_A_02_01	AMKAQFERDL	low
MHC_A_02_01	EMREQHDAQV	low
MHC_A_02_01	ELWKDVDRII	low
MHC_A_02_01	NLQKESRACL	low
MHC_A_02_01	ILRNQDDREL	low
MHC_A_02_01	LLSCLGCKIL	low
MHC_A_02_01	TACDWLFSHV	low
MHC_A_02_01	HLVEALYLVC	low
MHC_A_02_01	GLKSKTHAVL	low
MHC_A_02_01	LVEALYLVCG	low
MHC_A_02_01	YLVCGERGFF	low
MHC_A_02_01	NLNELVKHGL	low
MHC_A_02_01	PLNDNIATLL	low
MHC_A_02_01	ELFARSSDPR	low
MHC_A_02_01	IVDCLTEMYY	low
MHC_A_02_01	SRDSRGKPGY	low
MHC_A_02_01	GIVEQCCTSI	low
MHC_A_02_01	LTDAVKVMDL	low
MHC_A_02_01	TLPSWATEDT	low
MHC_A_02_01	EMRQKHSQAV	low
MHC_A_02_01	QAPGKGLEWV	low
MHC_A_02_01	ELEQTYHAKL	low
MHC_A_02_01	ETFGFEIQSY	low
MHC_A_02_01	MPIEPGDIGY	low
MHC_A_02_01	ELVKHGLRAL	low
MHC_A_02_01	SANMDWRSLT	low
MHC_A_02_01	DMEISAYRKL	low
MHC_A_02_01	PLESDAVECL	low
MHC_A_02_01	ELNGKNIEDV	low
MHC_A_02_01	ETMPCVKKKA	low

MHC_A_02_01	PLPVSRLVSL	low
MHC_A_02_01	QMNSLRAEDT	low
MHC_A_02_01	YADSVKGRFT	low
MHC_A_02_01	PLEGSEDRII	low
MHC_A_02_01	CTSICSLYQL	low
MHC_A_02_01	ALYLVCGERG	low
MHC_A_02_01	GLLECCARCLV	low
MHC_A_02_01	HLVEALYLVCG	low
MHC_A_02_01	SLCADARMYGV	low
MHC_A_02_01	RQAPGKGLEWV	low
MHC_A_02_01	HLCGSHLVEAL	low
MHC_A_02_01	LLVFACSAVPV	low
MHC_A_02_01	ITLEDSSGNLL	low
MHC_A_02_01	ALYLVCGERGF	low
MHC_A_02_01	ALFCGCGHEAL	low
MHC_A_02_01	VQLVESGGGLV	low
MHC_A_02_01	YFYYNAFHWAI	low
MHC_A_02_01	QMNSLRAEDTA	low
MHC_A_02_01	GSHLVEALYLV	low
MHC_A_02_01	CLVGAPFASLC	low
MHC_A_02_01	YLVCGERGFFY	low
MHC_A_02_01	YADSVKGRFTI	low
MHC_A_02_01	RLPGPSDTPIL	low
MHC_A_02_01	GSSDFQVHFLK	low
MHC_A_02_01	LEYFQFVKKLL	low
MHC_A_02_01	LPSCPTNFCIF	low
MHC_A_02_01	MIKYCLLKILK	low
MHC_A_02_01	MVRVLTVIKEY	low
MHC_A_02_01	RMILPMSRAFR	low
MHC_A_02_01	TFVPIAWAAAY	low
MHC_A_02_01	VALFSSCPVAY	low
MHC_A_02_01	YSLEYFQFVKK	low
MHC_A_02_01	RAEDTAVYYCA	low
MHC_A_02_01	WLGHPDKFVGI	low
MHC_A_02_01	DLSSCITQGKA	low
MHC_A_02_01	FLPRHRDTGIL	low
MHC_A_02_01	SLLMWITQCFL	low
MHC_A_02_01	LQMNSLRAEDT	low
MHC_A_02_01	EVQLVESGGGL	low
MHC_A_02_01	SLLMWITQCFLPVF	low
MHC_A_02_02	EMPPHIYAI	low
MHC_A_02_02	FLGMESCGI	low
MHC_A_02_02	GMFRTVGQL	low

MHC_A_02_02	NMDPLNDNI	low
MHC_A_02_02	DLTDYLMKI	low
MHC_A_02_02	TIWAANAGV	low
MHC_A_02_02	HLYQGCQVV	low
MHC_A_02_02	ILHNGAYSL	low
MHC_A_02_02	ALNKMFCQL	low
MHC_A_02_02	DLQPCIDLI	low
MHC_A_02_02	KTCPVQLWV	low
MHC_A_02_02	FRYNGLIHR	low
MHC_A_02_02	YLQMNSLRA	low
MHC_A_02_02	LLTFWNPPT	low
MHC_A_02_02	ALCRWGLLL	low
MHC_A_02_02	EADPTGHSY	low
MHC_A_02_02	YTAVVPLVY	low
MHC_A_02_02	EVDPIGHLY	low
MHC_A_02_02	KLRNWQWWRL	low
MHC_A_02_02	ILDLISESPI	low
MHC_A_02_02	RLFTKVKPLL	low
MHC_A_02_02	NLYRIGQSKV	low
MHC_A_02_02	NMDWRSLTQV	low
MHC_A_02_02	NLNELVKHGL	low
MHC_A_02_02	TLRNTNPNFV	low
MHC_A_02_02	YLWWVNNQSL	low
MHC_A_02_02	ELNDRLAVYI	low
MHC_A_02_02	ELRLLIHQSL	low
MHC_A_02_02	TMEAMRIMGI	low
MHC_A_02_02	ELPDGQVITI	low
MHC_A_02_02	KLKNKHEAMI	low
MHC_A_02_02	PLNDNIATLL	low
MHC_A_02_02	YLQLVFGIEV	low
MHC_A_02_02	PLPNFSSLNL	low
MHC_A_02_02	VLDQLRCNGV	low
MHC_A_02_02	GLKSKTHAVL	low
MHC_A_02_02	SLPLPNFSSL	low
MHC_A_02_02	TLKYPIEHGI	low
MHC_A_02_02	LLNVKMALDI	low
MHC_A_02_02	PLSPTRLSRL	low
MHC_A_02_02	RLPGPSDTPI	low
MHC_A_02_02	YMIMVKCWMI	low
MHC_A_02_02	RLSCAASGFT	low
MHC_A_02_02	VMAGVGSPYV	low
MHC_A_02_02	GLSQRHEEKV	low
MHC_A_02_02	NMKEEMARHL	low

MHC_A_02_02	EMREQHDAQV	low
MHC_A_02_02	LLQSKNAGAV	low
MHC_A_02_02	VLYGPDAPTI	low
MHC_A_02_02	NLPIYSEEIV	low
MHC_A_02_02	ELTCLNEASV	low
MHC_A_02_02	AMKAQFERDL	low
MHC_A_02_02	NLQKESRACL	low
MHC_A_02_02	GLDLQPCIDL	low
MHC_A_02_02	DLNSFEQLCI	low
MHC_A_02_02	LAPPQHLIRV	low
MHC_A_02_02	SANMDWRSLT	low
MHC_A_02_02	KMNPPKFSKV	low
MHC_A_02_02	ELRENTQTTI	low
MHC_A_02_02	ELEQTYHAKL	low
MHC_A_02_02	PLESDAVECL	low
MHC_A_02_02	ELNGKNIEDV	low
MHC_A_02_02	ELWKDVDRII	low
MHC_A_02_02	DLTDYLMKIL	low
MHC_A_02_02	EMRQKHSQAV	low
MHC_A_02_02	DMEISAYRKL	low
MHC_A_02_02	ELVKHGLRAL	low
MHC_A_02_02	QMNSLRAEDT	low
MHC_A_02_02	ETFGFEIQSY	low
MHC_A_02_02	QAPGKGLEWV	low
MHC_A_02_02	YADSVKGRFT	low
MHC_A_02_02	PLPVSRLVSL	low
MHC_A_02_02	PLEGSEDRII	low
MHC_A_02_02	QLVFGIELMEV	low
MHC_A_02_02	IMPKAGLLIIV	low
MHC_A_02_02	RLPGPSDTPIL	low
MHC_A_02_02	RQAPGKGLEWV	low
MHC_A_02_02	ITLEDSSGNLL	low
MHC_A_02_02	RAEDTAVYYCA	low
MHC_A_02_02	VQLVESGGGLV	low
MHC_A_02_02	YADSVKGRFTI	low
MHC_A_02_02	EVQLVESGGGL	low
MHC_A_02_02	LQMNSLRAEDT	low
MHC_A_02_03	LLTFWNPPT	low
MHC_A_02_03	GMFRTVGQL	low
MHC_A_02_03	ILHNGAYSL	low
MHC_A_02_03	EMPPHIYAI	low
MHC_A_02_03	GLENGLNYI	low
MHC_A_02_03	CQLAKTCPV	low

MHC_A_02_03	GMGQKDSYV	low
MHC_A_02_03	HLREYQDLL	low
MHC_A_02_03	KTCPVQLWV	low
MHC_A_02_03	RTFHIFYYL	low
MHC_A_02_03	ALCRWGLLL	low
MHC_A_02_03	TIWAANAGV	low
MHC_A_02_03	FRYNGLIHR	low
MHC_A_02_03	SMHFYGWSL	low
MHC_A_02_03	DLTDYLMKI	low
MHC_A_02_03	YQGSYGFRL	low
MHC_A_02_03	NMDPLNDNI	low
MHC_A_02_03	RRCPHHERC	low
MHC_A_02_03	DLQPCIDLI	low
MHC_A_02_03	YTAVVPLVY	low
MHC_A_02_03	FRHSVVVPY	low
MHC_A_02_03	FWAWSVLRV	low
MHC_A_02_03	GLEAYIQGI	low
MHC_A_02_03	GTEYRLTLY	low
MHC_A_02_03	GVRQFSGWM	low
MHC_A_02_03	IIYVGCGER	low
MHC_A_02_03	IYDFYYLDY	low
MHC_A_02_03	KVRDRNFQL	low
MHC_A_02_03	LQDDFDFNY	low
MHC_A_02_03	LVDENQSWY	low
MHC_A_02_03	MQDVFTFYV	low
MHC_A_02_03	NTDDFPLTL	low
MHC_A_02_03	NTFKFGVIY	low
MHC_A_02_03	RPNRQLGSM	low
MHC_A_02_03	RTDNGGWAH	low
MHC_A_02_03	RVYNNTARY	low
MHC_A_02_03	SAEVVTLWY	low
MHC_A_02_03	SILPISWAY	low
MHC_A_02_03	SVDSDHLGY	low
MHC_A_02_03	VRVCACPGR	low
MHC_A_02_03	WSTIWRQLY	low
MHC_A_02_03	WTDLFDNKV	low
MHC_A_02_03	YITDYSNDI	low
MHC_A_02_03	YSDNEMLTH	low
MHC_A_02_03	YTFCGTIEY	low
MHC_A_02_03	YTSDYFISY	low
MHC_A_02_03	EVDPIGHLY	low
MHC_A_02_03	HMSEFMECNL	low
MHC_A_02_03	VLDQLRCNGV	low

MHC_A_02_03	HLSWEWNLSI	low
MHC_A_02_03	FMECNLNELV	low
MHC_A_02_03	NLYRIGQSKV	low
MHC_A_02_03	YMIMVKCWMI	low
MHC_A_02_03	LLNVKMALDI	low
MHC_A_02_03	VLKEGSEYRV	low
MHC_A_02_03	VLQRNCAAYL	low
MHC_A_02_03	RLFTKVKPLL	low
MHC_A_02_03	TMEAMRIMGI	low
MHC_A_02_03	TLKYPIEHGI	low
MHC_A_02_03	NLNELVKHGL	low
MHC_A_02_03	FMDGKQACVL	low
MHC_A_02_03	YLLSGAGEHL	low
MHC_A_02_03	NLKERYYSGL	low
MHC_A_02_03	WLVLRINKAL	low
MHC_A_02_03	VLYGPDAPTI	low
MHC_A_02_03	YLQLVFGIEV	low
MHC_A_02_03	RLSCAASGFT	low
MHC_A_02_03	ELVKHGLRAL	low
MHC_A_02_03	MMQDREDQSI	low
MHC_A_02_03	KLKNKHEAMI	low
MHC_A_02_03	AMKAQFERDL	low
MHC_A_02_03	NMKEEMARHL	low
MHC_A_02_03	ELNGKNIEDV	low
MHC_A_02_03	KLRNWQWWRL	low
MHC_A_02_03	ELNDRLAVYI	low
MHC_A_02_03	QLKDKADFCI	low
MHC_A_02_03	ILDLISESPI	low
MHC_A_02_03	PLNDNIATLL	low
MHC_A_02_03	ELRLLIHQSL	low
MHC_A_02_03	GLSQRHEEKV	low
MHC_A_02_03	ELRENTQTTI	low
MHC_A_02_03	ELPDGQVITI	low
MHC_A_02_03	PLPNFSSLNL	low
MHC_A_02_03	PLSPTRLSRL	low
MHC_A_02_03	KLLEGEEERL	low
MHC_A_02_03	ELTCLNEASV	low
MHC_A_02_03	EMREQHDAQV	low
MHC_A_02_03	LAPPQHLIRV	low
MHC_A_02_03	NLQKESRACL	low
MHC_A_02_03	EMRQKHSQAV	low
MHC_A_02_03	QMNSLRAEDT	low
MHC_A_02_03	DLTDYLMKIL	low

MHC_A_02_03	SANMDWRSLT	low
MHC_A_02_03	PLESDAVECL	low
MHC_A_02_03	AMYVAIQAVL	low
MHC_A_02_03	QAPGKGLEWV	low
MHC_A_02_03	DLNSFEQLCI	low
MHC_A_02_03	GLDLQPCIDL	low
MHC_A_02_03	PLPVSRLVSL	low
MHC_A_02_03	ELWKDVDRII	low
MHC_A_02_03	DMEISAYRKL	low
MHC_A_02_03	YADSVKGRFT	low
MHC_A_02_03	NLPIYSEEIV	low
MHC_A_02_03	ETFGFEIQSY	low
MHC_A_02_03	PLEGSEDRII	low
MHC_A_02_03	ELEQTYHAKL	low
MHC_A_02_03	QMNSLRAEDTA	low
MHC_A_02_03	VQLVESGGGLV	low
MHC_A_02_03	QLVFGIELMEV	low
MHC_A_02_03	RQAPGKGLEWV	low
MHC_A_02_03	ITLEDSSGNLL	low
MHC_A_02_03	RLPGPSDTPIL	low
MHC_A_02_03	YADSVKGRFTI	low
MHC_A_02_03	RAEDTAVYYCA	low
MHC_A_02_03	EVQLVESGGGL	low
MHC_A_02_03	LQMNSLRAEDT	low
MHC_A_02_05	EADPTGHSY	low
MHC_A_02_06	ALNKMFCQL	low
MHC_A_02_06	EMPPHIYAI	low
MHC_A_02_06	CQLAKTCPV	low
MHC_A_02_06	FMECNLNEL	low
MHC_A_02_06	KMFCQLAKT	low
MHC_A_02_06	GLIYTYSGL	low
MHC_A_02_06	HLYQGCQVV	low
MHC_A_02_06	ILHNGAYSL	low
MHC_A_02_06	LLTFWNPPT	low
MHC_A_02_06	GLENGLNYI	low
MHC_A_02_06	SMHFYGWSL	low
MHC_A_02_06	RRCPHHERC	low
MHC_A_02_06	DLTDYLMKI	low
MHC_A_02_06	VRVCACPGR	low
MHC_A_02_06	FRYNGLIHR	low
MHC_A_02_06	HLREYQDLL	low
MHC_A_02_06	ALCRWGLLL	low
MHC_A_02_06	GMGQKDSYV	low

MHC_A_02_06	GMFRTVGQL	low
MHC_A_02_06	FRHSVVVPY	low
MHC_A_02_06	RVYNNTARY	low
MHC_A_02_06	DLQPCIDLI	low
MHC_A_02_06	YLQMNSLRA	low
MHC_A_02_06	NMDPLNDNI	low
MHC_A_02_06	YTAVVPLVY	low
MHC_A_02_06	EVDPIGHLY	low
MHC_A_02_06	RMFRWLVLRI	low
MHC_A_02_06	HLSWEWNLSI	low
MHC_A_02_06	FMECNLNELV	low
MHC_A_02_06	YMIMVKCWMI	low
MHC_A_02_06	KMNPPKFSKV	low
MHC_A_02_06	ALRTDYNASV	low
MHC_A_02_06	AMYVAIQAVL	low
MHC_A_02_06	YLQLVFGIEV	low
MHC_A_02_06	SLYASSPGGV	low
MHC_A_02_06	YLWWVNNQSL	low
MHC_A_02_06	WLVLRINKAL	low
MHC_A_02_06	LLNVKMALDI	low
MHC_A_02_06	TMEAMRIMGI	low
MHC_A_02_06	SLPLPNFSSL	low
MHC_A_02_06	FMDGKQACVL	low
MHC_A_02_06	SLSTFQQMWI	low
MHC_A_02_06	VLYGPDAPTI	low
MHC_A_02_06	VLDQLRCNGV	low
MHC_A_02_06	ELRLLIHQSL	low
MHC_A_02_06	MMQDREDQSI	low
MHC_A_02_06	LAPPQHLIRV	low
MHC_A_02_06	KLLEGEEERL	low
MHC_A_02_06	DLTDYLMKIL	low
MHC_A_02_06	VLKEGSEYRV	low
MHC_A_02_06	VLQRNCAAYL	low
MHC_A_02_06	ILDLISESPI	low
MHC_A_02_06	RLPGPSDTPI	low
MHC_A_02_06	VMAGVGSPYV	low
MHC_A_02_06	ELPDGQVITI	low
MHC_A_02_06	ELTCLNEASV	low
MHC_A_02_06	RLFTKVKPLL	low
MHC_A_02_06	ILHVDNHIGI	low
MHC_A_02_06	QAPGKGLEWV	low
MHC_A_02_06	KLRNWQWWRL	low
MHC_A_02_06	NLPIYSEEIV	low

MHC_A_02_06	AMKAQFERDL	low
MHC_A_02_06	PLNDNIATLL	low
MHC_A_02_06	NMKEEMARHL	low
MHC_A_02_06	LLQSKNAGAV	low
MHC_A_02_06	HMSEFMECNL	low
MHC_A_02_06	NLYRIGQSKV	low
MHC_A_02_06	GLDLQPCIDL	low
MHC_A_02_06	TLKYPIEHGI	low
MHC_A_02_06	ELNDRLAVYI	low
MHC_A_02_06	QLKDKADFCI	low
MHC_A_02_06	ELVKHGLRAL	low
MHC_A_02_06	SANMDWRSLT	low
MHC_A_02_06	KLKNKHEAMI	low
MHC_A_02_06	GLSQRHEEKV	low
MHC_A_02_06	PLPNFSSLNL	low
MHC_A_02_06	TLRNTNPNFV	low
MHC_A_02_06	NLKERYYSGL	low
MHC_A_02_06	ELRENTQTTI	low
MHC_A_02_06	EMREQHDAQV	low
MHC_A_02_06	PLEGSEDRII	low
MHC_A_02_06	PLESDAVECL	low
MHC_A_02_06	ELNGKNIEDV	low
MHC_A_02_06	ETFGFEIQSY	low
MHC_A_02_06	GLKSKTHAVL	low
MHC_A_02_06	NLNELVKHGL	low
MHC_A_02_06	PLPVSRLVSL	low
MHC_A_02_06	QMNSLRAEDT	low
MHC_A_02_06	RLSCAASGFT	low
MHC_A_02_06	YADSVKGRFT	low
MHC_A_02_06	DMEISAYRKL	low
MHC_A_02_06	ELEQTYHAKL	low
MHC_A_02_06	NLQKESRACL	low
MHC_A_02_06	PLSPTRLSRL	low
MHC_A_02_06	EMRQKHSQAV	low
MHC_A_02_06	ELWKDVDRII	low
MHC_A_02_06	DLNSFEQLCI	low
MHC_A_02_06	VQLVESGGGLV	low
MHC_A_02_06	IMPKAGLLIIV	low
MHC_A_02_06	QLVFGIELMEV	low
MHC_A_02_06	RQAPGKGLEWV	low
MHC_A_02_06	ITLEDSSGNLL	low
MHC_A_02_06	LQMNSLRAEDT	low
MHC_A_02_06	YADSVKGRFTI	low

MHC_A_02_06	RAEDTAVYYCA	low
MHC_A_02_06	RLPGPSDTPIL	low
MHC_A_02_06	EVQLVESGGGL	low
MHC_A_02_06	QMNSLRAEDTA	low
MHC_A_02_07	SIRDGVRAY	low
MHC_A_02_07	FIKDGSSTY	low
MHC_A_02_07	FAFVTDNTY	low
MHC_A_02_07	YLQLVFGIEV	low
MHC_A_02_07	GMPEGDLVYV	low
MHC_A_02_07	YMIDPSGVSY	low
MHC_A_02_07	LLFFWLDRSV	low
MHC_A_02_07	LTDAVKVMDL	low
MHC_A_02_07	TLPSWATEDT	low
MHC_A_02_11	ETFNTPAMY	low
MHC_A_02_11	FAAPHRGVA	low
MHC_A_02_11	FWAWSVLRV	low
MHC_A_02_11	GTEYRLTLY	low
MHC_A_02_11	IIYVGCGER	low
MHC_A_02_11	IYDFYYLDY	low
MHC_A_02_11	KVRDRNFQL	low
MHC_A_02_11	LQDDFDFNY	low
MHC_A_02_11	LVDENQSWY	low
MHC_A_02_11	RPNRQLGSM	low
MHC_A_02_11	RTDNGGWAH	low
MHC_A_02_11	SAEVVTLWY	low
MHC_A_02_11	SVDSDHLGY	low
MHC_A_02_11	TLKDGDFIL	low
MHC_A_02_11	WSTIWRQLY	low
MHC_A_02_11	WTDLFDNKV	low
MHC_A_02_11	YITDYSNDI	low
MHC_A_02_11	YSDNEMLTH	low
MHC_A_02_11	YTFCGTIEY	low
MHC_A_02_12	ETFNTPAMY	low
MHC_A_02_12	FAAPHRGVA	low
MHC_A_02_12	FWAWSVLRV	low
MHC_A_02_12	GTEYRLTLY	low
MHC_A_02_12	GVRQFSGWM	low
MHC_A_02_12	IIYVGCGER	low
MHC_A_02_12	IYDFYYLDY	low
MHC_A_02_12	KVRDRNFQL	low
MHC_A_02_12	LQDDFDFNY	low
MHC_A_02_12	LVDENQSWY	low
MHC_A_02_12	NTFKFGVIY	low

MHC_A_02_12	RPNRQLGSM	low
MHC_A_02_12	RTDNGGWAH	low
MHC_A_02_12	SAEVVTLWY	low
MHC_A_02_12	SILPISWAY	low
MHC_A_02_12	SLFNWLWYE	low
MHC_A_02_12	SVDSDHLGY	low
MHC_A_02_12	WSTIWRQLY	low
MHC_A_02_12	WTDLFDNKV	low
MHC_A_02_12	YSDNEMLTH	low
MHC_A_02_12	YTFCGTIEY	low
MHC_A_02_12	YTSDYFISY	low
MHC_A_02_16	YTYPCIPEY	low
MHC_A_02_16	FAAPHRGVA	low
MHC_A_02_16	FWAWSVLRV	low
MHC_A_02_16	GLEAYIQGI	low
MHC_A_02_16	GTEYRLTLY	low
MHC_A_02_16	GVRQFSGWM	low
MHC_A_02_16	IIYVGCGER	low
MHC_A_02_16	IYDFYYLDY	low
MHC_A_02_16	KVRDRNFQL	low
MHC_A_02_16	LQDDFDFNY	low
MHC_A_02_16	LVDENQSWY	low
MHC_A_02_16	RPNRQLGSM	low
MHC_A_02_16	RTDNGGWAH	low
MHC_A_02_16	SAEVVTLWY	low
MHC_A_02_16	SILPISWAY	low
MHC_A_02_16	SVDSDHLGY	low
MHC_A_02_16	TLKDGDFIL	low
MHC_A_02_16	WSTIWRQLY	low
MHC_A_02_16	YITDYSNDI	low
MHC_A_02_16	YSDNEMLTH	low
MHC_A_02_16	YTFCGTIEY	low
MHC_A_02_19	AMFIGHATA	low
MHC_A_02_19	ETFNTPAMY	low
MHC_A_02_19	FAAPHRGVA	low
MHC_A_02_19	FWAWSVLRV	low
MHC_A_02_19	GLEAYIQGI	low
MHC_A_02_19	GTEYRLTLY	low
MHC_A_02_19	GVRQFSGWM	low
MHC_A_02_19	IIYVGCGER	low
MHC_A_02_19	IYDFYYLDY	low
MHC_A_02_19	KVRDRNFQL	low
MHC_A_02_19	LQDDFDFNY	low

MHC_A_02_19	LVDENQSWY	low
MHC_A_02_19	NTFKFGVIY	low
MHC_A_02_19	RPNRQLGSM	low
MHC_A_02_19	RTDNGGWAH	low
MHC_A_02_19	SAEVVTLWY	low
MHC_A_02_19	SILPISWAY	low
MHC_A_02_19	SLFNWLWYE	low
MHC_A_02_19	SVDSDHLGY	low
MHC_A_02_19	TLKDGDFIL	low
MHC_A_02_19	WSTIWRQLY	low
MHC_A_02_19	WTDLFDNKV	low
MHC_A_02_19	YITDYSNDI	low
MHC_A_02_19	YSDNEMLTH	low
MHC_A_02_19	YTFCGTIEY	low
MHC_A_02_19	YTSDYFISY	low
MHC_A_02_19	YTYPCIPEY	low
MHC_A_02_50	ETFNTPAMY	low
MHC_A_02_50	YTSDYFISY	low
MHC_A_03_01	FYYNAFHW	low
MHC_A_03_01	YYNAFHWA	low
MHC_A_03_01	RVYNNTARY	low
MHC_A_03_01	RINGIPQQH	low
MHC_A_03_01	IIYVGCGER	low
MHC_A_03_01	TTINYTLWR	low
MHC_A_03_01	LVHFLLLKY	low
MHC_A_03_01	YLGSWATGK	low
MHC_A_03_01	YTSDYFISY	low
MHC_A_03_01	ISYPPLHER	low
MHC_A_03_01	TTINFTRQR	low
MHC_A_03_01	EVYEGVWKK	low
MHC_A_03_01	ILGPPGSVY	low
MHC_A_03_01	SSKMFNYFK	low
MHC_A_03_01	SQFGGGSQY	low
MHC_A_03_01	YTYPCIPEY	low
MHC_A_03_01	EVDPIGHLY	low
MHC_A_03_01	MIEPRTLQY	low
MHC_A_03_01	YTAVVPLVY	low
MHC_A_03_01	KLGGGQYGE	low
MHC_A_03_01	IADMGHLKY	low
MHC_A_03_01	STDHIPILY	low
MHC_A_03_01	AALDGTFQR	low
MHC_A_03_01	ATDFKFAMY	low
MHC_A_03_01	FRYNGLIHR	low

MHC_A_03_01	ETFNTPAMY	low
MHC_A_03_01	CSDDGFWSK	low
MHC_A_03_01	MCFHQHLMY	low
MHC_A_03_01	YTKVVHYRK	low
MHC_A_03_01	KSDLQPPNY	low
MHC_A_03_01	AMFIGHATA	low
MHC_A_03_01	CHKGWGVSV	low
MHC_A_03_01	CSEYVKDIY	low
MHC_A_03_01	DFDGTPRLY	low
MHC_A_03_01	DPSGAYFAW	low
MHC_A_03_01	DSDGSFFLY	low
MHC_A_03_01	EADPTGHSY	low
MHC_A_03_01	EPEPHILLF	low
MHC_A_03_01	FAAPHRGVA	low
MHC_A_03_01	FHMDPSGTF	low
MHC_A_03_01	FLFDRLTNG	low
MHC_A_03_01	FPEHIFPAL	low
MHC_A_03_01	FPHTELANL	low
MHC_A_03_01	FQILHDRFF	low
MHC_A_03_01	FQWHEAMFL	low
MHC_A_03_01	FWAWSVLRV	low
MHC_A_03_01	FYYNAFHWA	low
MHC_A_03_01	GHLENNPAL	low
MHC_A_03_01	GLEAYIQGI	low
MHC_A_03_01	GRNSFEVRV	low
MHC_A_03_01	GRPNCFQIV	low
MHC_A_03_01	GSEDRDLLY	low
MHC_A_03_01	GTEYRLTLY	low
MHC_A_03_01	GVRQFSGWM	low
MHC_A_03_01	IPYHIVNIV	low
MHC_A_03_01	IVDCLTEMY	low
MHC_A_03_01	IYDFYYLDY	low
MHC_A_03_01	KCDICTDEY	low
MHC_A_03_01	KVRDRNFQL	low
MHC_A_03_01	LMQCWQLLA	low
MHC_A_03_01	LPRWPPPQL	low
MHC_A_03_01	LQDDFDFNY	low
MHC_A_03_01	LVDENQSWY	low
MHC_A_03_01	LVYNHCEHG	low
MHC_A_03_01	MQDVFTFYV	low
MHC_A_03_01	NPNSPSITY	low
MHC_A_03_01	NQRETTVVW	low
MHC_A_03_01	NRRFVNVVP	low

MHC_A_03_01	NTDDFPLTL	low
MHC_A_03_01	NTFKFGVIY	low
MHC_A_03_01	RPNRQLGSM	low
MHC_A_03_01	RPRHQGVMV	low
MHC_A_03_01	RRFFPYYVY	low
MHC_A_03_01	RRHWGGNVL	low
MHC_A_03_01	RYFSVTRPL	low
MHC_A_03_01	SAEVVTLWY	low
MHC_A_03_01	SLFNWLWYE	low
MHC_A_03_01	SPRYIFTML	low
MHC_A_03_01	SRDKTIIMW	low
MHC_A_03_01	SVDSDHLGY	low
MHC_A_03_01	TEAEKWPFF	low
MHC_A_03_01	TLKDGDFIL	low
MHC_A_03_01	VGYVDDTQF	low
MHC_A_03_01	VLIRRCHYL	low
MHC_A_03_01	VLWKSYPLV	low
MHC_A_03_01	VVPSYIPLV	low
MHC_A_03_01	WSTIWRQLY	low
MHC_A_03_01	WTDLFDNKV	low
MHC_A_03_01	WVGRASDPD	low
MHC_A_03_01	YFDPANGKF	low
MHC_A_03_01	YFYYNAFHW	low
MHC_A_03_01	YGDTEAICR	low
MHC_A_03_01	YHEDIHTYL	low
MHC_A_03_01	YITDYSNDI	low
MHC_A_03_01	YLDDPDLKY	low
MHC_A_03_01	YLDFGGPEG	low
MHC_A_03_01	YPAEITLTW	low
MHC_A_03_01	YSDNEMLTH	low
MHC_A_03_01	YVDHYYRDY	low
MHC_A_03_01	YYFSYPLFV	low
MHC_A_03_01	YYNAFHWAI	low
MHC_A_03_01	GLENGLNYI	low
MHC_A_03_01	VVLASLIYRR	low
MHC_A_03_01	HTMEVTVYHR	low
MHC_A_03_01	LIGCWYCRRR	low
MHC_A_03_01	LLGDNQIMPK	low
MHC_A_03_01	YLGSANMDWR	low
MHC_A_03_01	IVDCLTEMYY	low
MHC_A_03_01	SVKGRFTISR	low
MHC_A_03_01	ELFARSSDPR	low
MHC_A_03_01	FYYNAFHWAI	low

MHC_A_03_01	SYMMDDLELI	low
MHC_A_03_01	PLALEGSLQK	low
MHC_A_03_01	VAALDGTFQR	low
MHC_A_03_01	GPGAGSLQPL	low
MHC_A_03_01	ETFGFEIQSY	low
MHC_A_03_01	RMILPMSRAFR	low
MHC_A_03_01	GSSDFQVHFLK	low
MHC_A_03_01	DSVKGRFTISR	low
MHC_A_03_01	FLLPILSQIYT	low
MHC_A_03_01	LEYFQFVKKLL	low
MHC_A_03_01	LPSCPTNFCIF	low
MHC_A_03_01	MIKYCLLKILK	low
MHC_A_03_01	MVRVLTVIKEY	low
MHC_A_03_01	SLLERGQQLGV	low
MHC_A_03_01	TFVPIAWAAAY	low
MHC_A_03_01	VALFSSCPVAY	low
MHC_A_03_01	YFYYNAFHWAI	low
MHC_A_03_01	YSLEYFQFVKK	low
MHC_A_03_01	GPGAGSLQPLA	low
MHC_A_03_01	GPGAGSLQPLAL	low
MHC_A_11_01	SSKMFNYFK	low
MHC_A_11_01	EVYEGVWKK	low
MHC_A_11_01	YTKVVHYRK	low
MHC_A_11_01	CSDDGFWSK	low
MHC_A_11_01	YTSDYFISY	low
MHC_A_11_01	ISYPPLHER	low
MHC_A_11_01	RTDITMKHK	low
MHC_A_11_01	STDHIPILY	low
MHC_A_11_01	LVHFLLLKY	low
MHC_A_11_01	SQFGGGSQY	low
MHC_A_11_01	YTYPCIPEY	low
MHC_A_11_01	YLGSWATGK	low
MHC_A_11_01	ILGPPGSVY	low
MHC_A_11_01	IVDCLTEMY	low
MHC_A_11_01	YTAVVPLVY	low
MHC_A_11_01	KLGGGQYGE	low
MHC_A_11_01	ATDFKFAMY	low
MHC_A_11_01	MIEPRTLQY	low
MHC_A_11_01	IADMGHLKY	low
MHC_A_11_01	EVDPIGHLY	low
MHC_A_11_01	GSEDRDLLY	low
MHC_A_11_01	MCFHQHLMY	low
MHC_A_11_01	RRFFPYYVY	low

MHC_A_11_01	CHKGWGVSV	low
MHC_A_11_01	CSEYVKDIY	low
MHC_A_11_01	DFDGTPRLY	low
MHC_A_11_01	DPSGAYFAW	low
MHC_A_11_01	EADPTGHSY	low
MHC_A_11_01	EPEPHILLF	low
MHC_A_11_01	FHMDPSGTF	low
MHC_A_11_01	FPEHIFPAL	low
MHC_A_11_01	FPHTELANL	low
MHC_A_11_01	FQILHDRFF	low
MHC_A_11_01	GHLENNPAL	low
MHC_A_11_01	GRPNCFQIV	low
MHC_A_11_01	IPYHIVNIV	low
MHC_A_11_01	KCDICTDEY	low
MHC_A_11_01	KSDLQPPNY	low
MHC_A_11_01	LPRWPPPQL	low
MHC_A_11_01	LVYNHCEHG	low
MHC_A_11_01	NPNSPSITY	low
MHC_A_11_01	NQRETTVVW	low
MHC_A_11_01	NRRFVNVVP	low
MHC_A_11_01	RPRHQGVMV	low
MHC_A_11_01	RRHWGGNVL	low
MHC_A_11_01	RYFSVTRPL	low
MHC_A_11_01	SPRYIFTML	low
MHC_A_11_01	SRDKTIIMW	low
MHC_A_11_01	TEAEKWPFF	low
MHC_A_11_01	VGYVDDTQF	low
MHC_A_11_01	WVGRASDPD	low
MHC_A_11_01	YGDTEAICR	low
MHC_A_11_01	YHEDIHTYL	low
MHC_A_11_01	YLDDRNTFR	low
MHC_A_11_01	YLDFGGPEG	low
MHC_A_11_01	YPAEITLTW	low
MHC_A_11_01	YVDHYYRDY	low
MHC_A_11_01	DSDGSFFLY	low
MHC_A_11_01	GLENGLNYI	low
MHC_A_11_01	FRYNGLIHR	low
MHC_A_11_01	YLDDPDLKY	low
MHC_A_11_01	GRNSFEVRV	low
MHC_A_11_01	ETFNTPAMY	low
MHC_A_11_01	YFDPANGKF	low
MHC_A_11_01	SVKGRFTISR	low
MHC_A_11_01	VVLASLIYRR	low

MHC_A_11_01	IVDCLTEMYY	low
MHC_A_11_01	IALNFPGSQK	low
MHC_A_11_01	LLGDNQIMPK	low
MHC_A_11_01	VAALDGTFQR	low
MHC_A_11_01	YLGSANMDWR	low
MHC_A_11_01	GPGAGSLQPL	low
MHC_A_11_01	ELFARSSDPR	low
MHC_A_11_01	SYMMDDLELI	low
MHC_A_11_01	PLALEGSLQK	low
MHC_A_11_01	ETFGFEIQSY	low
MHC_A_11_01	GSSDFQVHFLK	low
MHC_A_11_01	MIKYCLLKILK	low
MHC_A_11_01	MVRVLTVIKEY	low
MHC_A_11_01	RMILPMSRAFR	low
MHC_A_11_01	YSLEYFQFVKK	low
MHC_A_11_01	DSVKGRFTISR	low
MHC_A_11_01	FLLPILSQIYT	low
MHC_A_11_01	LEYFQFVKKLL	low
MHC_A_11_01	LPSCPTNFCIF	low
MHC_A_11_01	SLLERGQQLGV	low
MHC_A_11_01	TFVPIAWAAAY	low
MHC_A_11_01	VALFSSCPVAY	low
MHC_A_11_01	YFYYNAFHWAI	low
MHC_A_11_01	GPGAGSLQPLA	low
MHC_A_11_01	GPGAGSLQPLAL	low
MHC_A_23_01	FRYNGLIHR	low
MHC_A_23_01	RAEDTAVYY	low
MHC_A_23_01	AMFIGHATA	low
MHC_A_23_01	FAAPHRGVA	low
MHC_A_23_01	GLEAYIQGI	low
MHC_A_23_01	GTEYRLTLY	low
MHC_A_23_01	GVRQFSGWM	low
MHC_A_23_01	IIYVGCGER	low
MHC_A_23_01	LMQCWQLLA	low
MHC_A_23_01	LQDDFDFNY	low
MHC_A_23_01	MQDVFTFYV	low
MHC_A_23_01	RPNRQLGSM	low
MHC_A_23_01	RTDNGGWAH	low
MHC_A_23_01	SAEVVTLWY	low
MHC_A_23_01	SVDSDHLGY	low
MHC_A_23_01	TLKDGDFIL	low
MHC_A_23_01	WTDLFDNKV	low
MHC_A_23_01	YITDYSNDI	low

MHC_A_23_01	YSDNEMLTH	low
MHC_A_23_01	YTFCGTIEY	low
MHC_A_23_01	YTAVVPLVY	low
MHC_A_23_01	LRAEDTAVY	low
MHC_A_23_01	GQRKGAGSVF	low
MHC_A_23_01	LRAEDTAVYY	low
MHC_A_23_01	RLPGPSDTPI	low
MHC_A_23_01	ETFGFEIQSY	low
MHC_A_23_01	SLRAEDTAVY	low
MHC_A_23_01	RLSCAASGFTF	low
MHC_A_23_01	SLRLSCAASGF	low
MHC_A_23_01	CAASGFTFSSY	low
MHC_A_23_01	NSLRAEDTAVY	low
MHC_A_23_01	SLRAEDTAVYY	low
MHC_A_23_01	RLPGPSDTPIL	low
MHC_A_23_01	EVQLVESGGGL	low
MHC_A_24_02	YYNAFHWA	low
MHC_A_24_02	FYYNAFHW	low
MHC_A_24_02	SYLDSGIHF	low
MHC_A_24_02	NYARTEDFF	low
MHC_A_24_02	YYNAFHWAI	low
MHC_A_24_02	NYPLWSQSY	low
MHC_A_24_02	LYSDPADYF	low
MHC_A_24_02	TEAEKWPFF	low
MHC_A_24_02	FYYNAFHWA	low
MHC_A_24_02	MFPDLESEF	low
MHC_A_24_02	YFYYNAFHW	low
MHC_A_24_02	MILPMSRAF	low
MHC_A_24_02	FRYNGLIHR	low
MHC_A_24_02	FPHTELANL	low
MHC_A_24_02	FQILHDRFF	low
MHC_A_24_02	SSKMFNYFK	low
MHC_A_24_02	RAEDTAVYY	low
MHC_A_24_02	YTAVVPLVY	low
MHC_A_24_02	LRAEDTAVY	low
MHC_A_24_02	SYVKVLHHTL	low
MHC_A_24_02	LSCAASGFTF	low
MHC_A_24_02	FYDPMFKYHL	low
MHC_A_24_02	VMPKTGLLII	low
MHC_A_24_02	FYYNAFHWAI	low
MHC_A_24_02	SFSTTINYTL	low
MHC_A_24_02	KYAGESFPGI	low
MHC_A_24_02	LYILVTCLGL	low

MHC_A_24_02	LYNFTQIPHL	low
MHC_A_24_02	GQRKGAGSVF	low
MHC_A_24_02	RLPGPSDTPI	low
MHC_A_24_02	ELFARSSDPR	low
MHC_A_24_02	IVDCLTEMYY	low
MHC_A_24_02	SLRAEDTAVY	low
MHC_A_24_02	LRAEDTAVYY	low
MHC_A_24_02	ETFGFEIQSY	low
MHC_A_24_02	TFVPIAWAAAY	low
MHC_A_24_02	RLSCAASGFTF	low
MHC_A_24_02	LPSCPTNFCIF	low
MHC_A_24_02	YFYYNAFHWAI	low
MHC_A_24_02	RMILPMSRAFR	low
MHC_A_24_02	SLRLSCAASGF	low
MHC_A_24_02	CAASGFTFSSY	low
MHC_A_24_02	SLLERGQQLGV	low
MHC_A_24_02	RLPGPSDTPIL	low
MHC_A_24_02	FLLPILSQIYT	low
MHC_A_24_02	GSSDFQVHFLK	low
MHC_A_24_02	LEYFQFVKKLL	low
MHC_A_24_02	MIKYCLLKILK	low
MHC_A_24_02	MVRVLTVIKEY	low
MHC_A_24_02	VALFSSCPVAY	low
MHC_A_24_02	YSLEYFQFVKK	low
MHC_A_24_02	SLRAEDTAVYY	low
MHC_A_24_02	EVQLVESGGGL	low
MHC_A_24_02	NSLRAEDTAVY	low
MHC_A_24_03	AMFIGHATA	low
MHC_A_24_03	FAAPHRGVA	low
MHC_A_24_03	FQWHEAMFL	low
MHC_A_24_03	FWAWSVLRV	low
MHC_A_24_03	GLEAYIQGI	low
MHC_A_24_03	GTEYRLTLY	low
MHC_A_24_03	GVRQFSGWM	low
MHC_A_24_03	IIYVGCGER	low
MHC_A_24_03	IYDFYYLDY	low
MHC_A_24_03	KVRDRNFQL	low
MHC_A_24_03	LMQCWQLLA	low
MHC_A_24_03	LQDDFDFNY	low
MHC_A_24_03	MQDVFTFYV	low
MHC_A_24_03	NTDDFPLTL	low
MHC_A_24_03	NTFKFGVIY	low
MHC_A_24_03	RPNRQLGSM	low

MHC_A_24_03	RTDNGGWAH	low
MHC_A_24_03	SAEVVTLWY	low
MHC_A_24_03	SVDSDHLGY	low
MHC_A_24_03	TLKDGDFIL	low
MHC_A_24_03	VLIRRCHYL	low
MHC_A_24_03	VLWKSYPLV	low
MHC_A_24_03	WTDLFDNKV	low
MHC_A_24_03	YITDYSNDI	low
MHC_A_24_03	YSDNEMLTH	low
MHC_A_24_03	YTFCGTIEY	low
MHC_A_25_01	AMFIGHATA	low
MHC_A_25_01	FAAPHRGVA	low
MHC_A_25_01	FLFDRLTNG	low
MHC_A_25_01	FPHTELANL	low
MHC_A_25_01	FQILHDRFF	low
MHC_A_25_01	FQWHEAMFL	low
MHC_A_25_01	FWAWSVLRV	low
MHC_A_25_01	GLEAYIQGI	low
MHC_A_25_01	GTEYRLTLY	low
MHC_A_25_01	GVRQFSGWM	low
MHC_A_25_01	IIYVGCGER	low
MHC_A_25_01	IYDFYYLDY	low
MHC_A_25_01	KVRDRNFQL	low
MHC_A_25_01	LMQCWQLLA	low
MHC_A_25_01	LQDDFDFNY	low
MHC_A_25_01	LVDENQSWY	low
MHC_A_25_01	MQDVFTFYV	low
MHC_A_25_01	NTDDFPLTL	low
MHC_A_25_01	NTFKFGVIY	low
MHC_A_25_01	RPNRQLGSM	low
MHC_A_25_01	RTDNGGWAH	low
MHC_A_25_01	SAEVVTLWY	low
MHC_A_25_01	SILPISWAY	low
MHC_A_25_01	SLFNWLWYE	low
MHC_A_25_01	SSKMFNYFK	low
MHC_A_25_01	TEAEKWPFF	low
MHC_A_25_01	TLKDGDFIL	low
MHC_A_25_01	VLIRRCHYL	low
MHC_A_25_01	VLWKSYPLV	low
MHC_A_25_01	VVPSYIPLV	low
MHC_A_25_01	WSTIWRQLY	low
MHC_A_25_01	WTDLFDNKV	low
MHC_A_25_01	YITDYSNDI	low

MHC_A_25_01	YSDNEMLTH	low
MHC_A_25_01	YYFSYPLFV	low
MHC_A_25_01	ELFARSSDPR	low
MHC_A_25_01	IVDCLTEMYY	low
MHC_A_25_01	SYMMDDLELI	low
MHC_A_25_01	MVRVLTVIKEY	low
MHC_A_25_01	FLLPILSQIYT	low
MHC_A_25_01	GSSDFQVHFLK	low
MHC_A_25_01	LEYFQFVKKLL	low
MHC_A_25_01	LPSCPTNFCIF	low
MHC_A_25_01	MIKYCLLKILK	low
MHC_A_25_01	RMILPMSRAFR	low
MHC_A_25_01	SLLERGQQLGV	low
MHC_A_25_01	TFVPIAWAAAY	low
MHC_A_25_01	VALFSSCPVAY	low
MHC_A_25_01	YFYYNAFHWAI	low
MHC_A_25_01	YSLEYFQFVKK	low
MHC_A_26_01	RVYNNTARY	low
MHC_A_26_01	YTAVVPLVY	low
MHC_A_26_01	FTWPWMKLF	low
MHC_A_26_01	RAEDTAVYY	low
MHC_A_26_01	AMFIGHATA	low
MHC_A_26_01	FAAPHRGVA	low
MHC_A_26_01	FLFDRLTNG	low
MHC_A_26_01	FQWHEAMFL	low
MHC_A_26_01	FRHSVVVPY	low
MHC_A_26_01	FWAWSVLRV	low
MHC_A_26_01	GLEAYIQGI	low
MHC_A_26_01	GTEYRLTLY	low
MHC_A_26_01	IYDFYYLDY	low
MHC_A_26_01	KVRDRNFQL	low
MHC_A_26_01	LMQCWQLLA	low
MHC_A_26_01	LQDDFDFNY	low
MHC_A_26_01	LQIRGRERF	low
MHC_A_26_01	LVDENQSWY	low
MHC_A_26_01	MQDVFTFYV	low
MHC_A_26_01	NTDDFPLTL	low
MHC_A_26_01	RPNRQLGSM	low
MHC_A_26_01	RRCPHHERC	low
MHC_A_26_01	RTDNGGWAH	low
MHC_A_26_01	SAEVVTLWY	low
MHC_A_26_01	SLFNWLWYE	low
MHC_A_26_01	TLKDGDFIL	low

MHC_A_26_01	VLIRRCHYL	low
MHC_A_26_01	VLWKSYPLV	low
MHC_A_26_01	VRVCACPGR	low
MHC_A_26_01	WSTIWRQLY	low
MHC_A_26_01	WTDLFDNKV	low
MHC_A_26_01	YITDYSNDI	low
MHC_A_26_01	YSDNEMLTH	low
MHC_A_26_01	YYFSYPLFV	low
MHC_A_26_01	LRAEDTAVY	low
MHC_A_26_01	FRYNGLIHR	low
MHC_A_26_01	LSCAASGFTF	low
MHC_A_26_01	SLRAEDTAVY	low
MHC_A_26_01	GQRKGAGSVF	low
MHC_A_26_01	YYADSVKGRF	low
MHC_A_26_01	LRAEDTAVYY	low
MHC_A_26_01	RLPGPSDTPI	low
MHC_A_26_01	CAASGFTFSSY	low
MHC_A_26_01	RLSCAASGFTF	low
MHC_A_26_01	SLRAEDTAVYY	low
MHC_A_26_01	NSLRAEDTAVY	low
MHC_A_26_01	SLRLSCAASGF	low
MHC_A_26_01	EVQLVESGGGL	low
MHC_A_26_01	RLPGPSDTPIL	low
MHC_A_26_02	FTWPWMKLF	low
MHC_A_26_02	RVYNNTARY	low
MHC_A_26_02	RRCPHHERC	low
MHC_A_26_02	FRHSVVVPY	low
MHC_A_26_02	LQIRGRERF	low
MHC_A_26_02	VRVCACPGR	low
MHC_A_26_03	FRHSVVVPY	low
MHC_A_26_03	LQIRGRERF	low
MHC_A_26_03	RRCPHHERC	low
MHC_A_26_03	RVYNNTARY	low
MHC_A_29_02	NYIDKVRFL	low
MHC_A_29_02	FIKDGSSTY	low
MHC_A_29_02	RAEDTAVYY	low
MHC_A_29_02	SIRDGVRAY	low
MHC_A_29_02	SSKMFNYFK	low
MHC_A_29_02	LRAEDTAVY	low
MHC_A_29_02	FQILHDRFF	low
MHC_A_29_02	AMFIGHATA	low
MHC_A_29_02	FAAPHRGVA	low
MHC_A_29_02	FPHTELANL	low

MHC_A_29_02	GLEAYIQGI	low
MHC_A_29_02	KVRDRNFQL	low
MHC_A_29_02	LQDDFDFNY	low
MHC_A_29_02	MQDVFTFYV	low
MHC_A_29_02	RPNRQLGSM	low
MHC_A_29_02	RTDNGGWAH	low
MHC_A_29_02	SAEVVTLWY	low
MHC_A_29_02	TEAEKWPFF	low
MHC_A_29_02	TLKDGDFIL	low
MHC_A_29_02	VLIRRCHYL	low
MHC_A_29_02	VVPSYIPLV	low
MHC_A_29_02	YITDYSNDI	low
MHC_A_29_02	YSDNEMLTH	low
MHC_A_29_02	KAGQVVTIW	low
MHC_A_29_02	FRYNGLIHR	low
MHC_A_29_02	AELLNIPFLY	low
MHC_A_29_02	SYMMDDLELI	low
MHC_A_29_02	LSCAASGFTF	low
MHC_A_29_02	LRAEDTAVYY	low
MHC_A_29_02	SLRAEDTAVY	low
MHC_A_29_02	ETFGFEIQSY	low
MHC_A_29_02	ELFARSSDPR	low
MHC_A_29_02	GQRKGAGSVF	low
MHC_A_29_02	RLPGPSDTPI	low
MHC_A_29_02	ITSQDVLHSW	low
MHC_A_29_02	CAASGFTFSSY	low
MHC_A_29_02	NSLRAEDTAVY	low
MHC_A_29_02	FLLPILSQIYT	low
MHC_A_29_02	SLRAEDTAVYY	low
MHC_A_29_02	YFYYNAFHWAI	low
MHC_A_29_02	GSSDFQVHFLK	low
MHC_A_29_02	SLRLSCAASGF	low
MHC_A_29_02	RLSCAASGFTF	low
MHC_A_29_02	SLLERGQQLGV	low
MHC_A_29_02	LEYFQFVKKLL	low
MHC_A_29_02	LPSCPTNFCIF	low
MHC_A_29_02	MIKYCLLKILK	low
MHC_A_29_02	RMILPMSRAFR	low
MHC_A_29_02	YSLEYFQFVKK	low
MHC_A_29_02	RLPGPSDTPIL	low
MHC_A_29_02	EVQLVESGGGL	low
MHC_A_29_02	ISDSNPFLTQW	low
MHC_A_30_01	HASPTLFNA	low

MHC_A_30_01	SYGFRLGFL	low
MHC_A_30_01	VRVCACPGR	low
MHC_A_30_01	FRHSVVVPY	low
MHC_A_30_01	FRYNGLIHR	low
MHC_A_30_01	KLGGGQYGE	low
MHC_A_30_01	ETFNTPAMY	low
MHC_A_30_01	EYLDDRNTF	low
MHC_A_30_01	LQIRGRERF	low
MHC_A_30_01	YTSDYFISY	low
MHC_A_30_01	YTYPCIPEY	low
MHC_A_30_01	GLENGLNYI	low
MHC_A_30_01	YTAVVPLVY	low
MHC_A_30_01	EVDPIGHLY	low
MHC_A_30_01	EVYEGVWKK	low
MHC_A_30_01	ETFGFEIQSY	low
MHC_A_30_01	PLALEGSLQK	low
MHC_A_30_01	GPGAGSLQPLAL	low
MHC_A_30_02	YTAVVPLVY	low
MHC_A_30_02	LRAEDTAVY	low
MHC_A_30_02	FRYNGLIHR	low
MHC_A_30_02	RAEDTAVYY	low
MHC_A_30_02	ETFGFEIQSY	low
MHC_A_30_02	SLRAEDTAVY	low
MHC_A_30_02	YYADSVKGRF	low
MHC_A_30_02	LRAEDTAVYY	low
MHC_A_30_02	GQRKGAGSVF	low
MHC_A_30_02	LSCAASGFTF	low
MHC_A_30_02	RLPGPSDTPI	low
MHC_A_30_02	NSLRAEDTAVY	low
MHC_A_30_02	SLRAEDTAVYY	low
MHC_A_30_02	RLPGPSDTPIL	low
MHC_A_30_02	SLRLSCAASGF	low
MHC_A_30_02	RLSCAASGFTF	low
MHC_A_30_02	EVQLVESGGGL	low
MHC_A_31_01	TTINYTLWR	low
MHC_A_31_01	SSKMFNYFK	low
MHC_A_31_01	FRYNGLIHR	low
MHC_A_31_01	RVYNNTARY	low
MHC_A_31_01	RYFSVTRPL	low
MHC_A_31_01	YLGSWATGK	low
MHC_A_31_01	KLGGGQYGE	low
MHC_A_31_01	ATDFKFAMY	low
MHC_A_31_01	CHKGWGVSV	low

MHC_A_31_01	CSDDGFWSK	low
MHC_A_31_01	CSEYVKDIY	low
MHC_A_31_01	DFDGTPRLY	low
MHC_A_31_01	DPSGAYFAW	low
MHC_A_31_01	DSDGSFFLY	low
MHC_A_31_01	EADPTGHSY	low
MHC_A_31_01	EPEPHILLF	low
MHC_A_31_01	ETFNTPAMY	low
MHC_A_31_01	FHMDPSGTF	low
MHC_A_31_01	FPEHIFPAL	low
MHC_A_31_01	FPHTELANL	low
MHC_A_31_01	FQILHDRFF	low
MHC_A_31_01	GHLENNPAL	low
MHC_A_31_01	GRNSFEVRV	low
MHC_A_31_01	GRPNCFQIV	low
MHC_A_31_01	GSEDRDLLY	low
MHC_A_31_01	IADMGHLKY	low
MHC_A_31_01	ILGPPGSVY	low
MHC_A_31_01	IPYHIVNIV	low
MHC_A_31_01	IVDCLTEMY	low
MHC_A_31_01	KCDICTDEY	low
MHC_A_31_01	KSDLQPPNY	low
MHC_A_31_01	LPRWPPPQL	low
MHC_A_31_01	LVYNHCEHG	low
MHC_A_31_01	MCFHQHLMY	low
MHC_A_31_01	MIEPRTLQY	low
MHC_A_31_01	NPNSPSITY	low
MHC_A_31_01	NQRETTVVW	low
MHC_A_31_01	NRRFVNVVP	low
MHC_A_31_01	RPRHQGVMV	low
MHC_A_31_01	RRFFPYYVY	low
MHC_A_31_01	RRHWGGNVL	low
MHC_A_31_01	SPRYIFTML	low
MHC_A_31_01	SQFGGGSQY	low
MHC_A_31_01	SRDKTIIMW	low
MHC_A_31_01	STDHIPILY	low
MHC_A_31_01	TEAEKWPFF	low
MHC_A_31_01	VGYVDDTQF	low
MHC_A_31_01	WVGRASDPD	low
MHC_A_31_01	YFDPANGKF	low
MHC_A_31_01	YGDTEAICR	low
MHC_A_31_01	YHEDIHTYL	low
MHC_A_31_01	YLDDPDLKY	low

MHC_A_31_01	YLDFGGPEG	low
MHC_A_31_01	YPAEITLTW	low
MHC_A_31_01	YTSDYFISY	low
MHC_A_31_01	YTYPCIPEY	low
MHC_A_31_01	YVDHYYRDY	low
MHC_A_31_01	ALDGTFQRK	low
MHC_A_31_01	YTAVVPLVY	low
MHC_A_31_01	GLENGLNYI	low
MHC_A_31_01	EVDPIGHLY	low
MHC_A_31_01	EVYEGVWKK	low
MHC_A_31_01	VAALDGTFQR	low
MHC_A_31_01	YLGSANMDWR	low
MHC_A_31_01	AALDGTFQRK	low
MHC_A_31_01	ETFGFEIQSY	low
MHC_A_31_01	IALNFPGSQK	low
MHC_A_31_01	ELFARSSDPR	low
MHC_A_31_01	IVDCLTEMYY	low
MHC_A_31_01	SYMMDDLELI	low
MHC_A_31_01	PLALEGSLQK	low
MHC_A_31_01	DSVKGRFTISR	low
MHC_A_31_01	RMILPMSRAFR	low
MHC_A_31_01	GSSDFQVHFLK	low
MHC_A_31_01	MIKYCLLKILK	low
MHC_A_31_01	FLLPILSQIYT	low
MHC_A_31_01	LEYFQFVKKLL	low
MHC_A_31_01	LPSCPTNFCIF	low
MHC_A_31_01	MVRVLTVIKEY	low
MHC_A_31_01	SLLERGQQLGV	low
MHC_A_31_01	TFVPIAWAAAY	low
MHC_A_31_01	VALFSSCPVAY	low
MHC_A_31_01	YFYYNAFHWAI	low
MHC_A_31_01	YSLEYFQFVKK	low
MHC_A_31_01	GPGAGSLQPLAL	low
MHC_A_32_01	ILGPPGSVY	low
MHC_A_32_01	YTAVVPLVY	low
MHC_A_32_01	FRYNGLIHR	low
MHC_A_32_01	VLKPGMVVTF	low
MHC_A_32_01	ETFGFEIQSY	low
MHC_A_32_01	IQPGRGFVLY	low
MHC_A_32_15	MPMKGRFPI	low
MHC_A_33_01	EVYEGVWKK	low
MHC_A_33_01	AALDGTFQR	low
MHC_A_33_01	TTINYTLWR	low

MHC_A_33_01	ISYPPLHER	low
MHC_A_33_01	FRYNGLIHR	low
MHC_A_33_01	YTAVVPLVY	low
MHC_A_33_01	KLGGGQYGE	low
MHC_A_33_01	ALDGTFQRK	low
MHC_A_33_01	EVDPIGHLY	low
MHC_A_33_01	GLENGLNYI	low
MHC_A_33_01	SVKGRFTISR	low
MHC_A_33_01	VAALDGTFQR	low
MHC_A_33_01	ETFGFEIQSY	low
MHC_A_33_01	YLGSANMDWR	low
MHC_A_33_01	IALNFPGSQK	low
MHC_A_33_01	VQKIFHINPR	low
MHC_A_33_01	AALDGTFQRK	low
MHC_A_33_01	PLALEGSLQK	low
MHC_A_33_01	DSVKGRFTISR	low
MHC_A_33_01	GPGAGSLQPLAL	low
MHC_A_66_01	PLALEGSLQK	low
MHC_A_66_01	GPGAGSLQPLAL	low
MHC_A_68_01	FTFSSYGMH	low
MHC_A_68_01	ISYPPLHER	low
MHC_A_68_01	YTAVVPLVY	low
MHC_A_68_01	FRYNGLIHR	low
MHC_A_68_01	KLGGGQYGE	low
MHC_A_68_01	EVDPIGHLY	low
MHC_A_68_01	TLYLQMNSL	low
MHC_A_68_01	RAEDTAVYY	low
MHC_A_68_01	GLENGLNYI	low
MHC_A_68_01	ALDGTFQRK	low
MHC_A_68_01	LRAEDTAVY	low
MHC_A_68_01	VRQAPGKGL	low
MHC_A_68_01	ISYLGRDRLR	low
MHC_A_68_01	YLGSANMDWR	low
MHC_A_68_01	GLEWVAVIWY	low
MHC_A_68_01	LRLSCAASGF	low
MHC_A_68_01	IALNFPGSQK	low
MHC_A_68_01	AALDGTFQRK	low
MHC_A_68_01	IWYDGSNKYY	low
MHC_A_68_01	LISACKDGKR	low
MHC_A_68_01	LRAEDTAVYY	low
MHC_A_68_01	PLALEGSLQK	low
MHC_A_68_01	WVRQAPGKGL	low
MHC_A_68_01	ISRDNSKNTL	low

MHC_A_68_01	DSVKGRFTISR	low
MHC_A_68_01	GPGAGSLQPLAL	low
MHC_A_68_02	YSLEYFQFV	low
MHC_A_68_02	RTFHIFYYL	low
MHC_A_68_02	YLYVDKNFI	low
MHC_A_68_02	FPHTELANL	low
MHC_A_68_02	YTAVVPLVY	low
MHC_A_68_02	SMHFYGWSL	low
MHC_A_68_02	HLYQGCQVV	low
MHC_A_68_02	DLQPCIDLI	low
MHC_A_68_02	HLREYQDLL	low
MHC_A_68_02	FLSNGHVTI	low
MHC_A_68_02	ILAKFLHWL	low
MHC_A_68_02	FLGMESCGI	low
MHC_A_68_02	DLTDYLMKI	low
MHC_A_68_02	FMDGKQACV	low
MHC_A_68_02	FMECNLNEL	low
MHC_A_68_02	ALCRWGLLL	low
MHC_A_68_02	ALNKMFCQL	low
MHC_A_68_02	CQLAKTCPV	low
MHC_A_68_02	ILHNGAYSL	low
MHC_A_68_02	KMFCQLAKT	low
MHC_A_68_02	KTCPVQLWV	low
MHC_A_68_02	LLTFWNPPT	low
MHC_A_68_02	GMGQKDSYV	low
MHC_A_68_02	FLFDRLTNG	low
MHC_A_68_02	FQILHDRFF	low
MHC_A_68_02	FQWHEAMFL	low
MHC_A_68_02	FWAWSVLRV	low
MHC_A_68_02	GLEAYIQGI	low
MHC_A_68_02	GTEYRLTLY	low
MHC_A_68_02	GVRQFSGWM	low
MHC_A_68_02	IIYVGCGER	low
MHC_A_68_02	IYDFYYLDY	low
MHC_A_68_02	KVRDRNFQL	low
MHC_A_68_02	LMQCWQLLA	low
MHC_A_68_02	LQDDFDFNY	low
MHC_A_68_02	LVDENQSWY	low
MHC_A_68_02	RPNRQLGSM	low
MHC_A_68_02	RTDNGGWAH	low
MHC_A_68_02	RVYNNTARY	low
MHC_A_68_02	SAEVVTLWY	low
MHC_A_68_02	SILPISWAY	low

MHC_A_68_02	SLFNWLWYE	low
MHC_A_68_02	SSKMFNYFK	low
MHC_A_68_02	SVDSDHLGY	low
MHC_A_68_02	TEAEKWPFF	low
MHC_A_68_02	TLKDGDFIL	low
MHC_A_68_02	VLIRRCHYL	low
MHC_A_68_02	VLWKSYPLV	low
MHC_A_68_02	WSTIWRQLY	low
MHC_A_68_02	YITDYSNDI	low
MHC_A_68_02	YSDNEMLTH	low
MHC_A_68_02	GMFRTVGQL	low
MHC_A_68_02	YLQMNSLRA	low
MHC_A_68_02	EVDPIGHLY	low
MHC_A_68_02	FRYNGLIHR	low
MHC_A_68_02	GLENGLNYI	low
MHC_A_68_02	NMDPLNDNI	low
MHC_A_68_02	ELNDRLAVYI	low
MHC_A_68_02	YLWWVNNQSL	low
MHC_A_68_02	QLFNHTMFIL	low
MHC_A_68_02	ELTCLNEASV	low
MHC_A_68_02	ELPDGQVITI	low
MHC_A_68_02	ETFGFEIQSY	low
MHC_A_68_02	AMYVAIQAVL	low
MHC_A_68_02	SLSTFQQMWI	low
MHC_A_68_02	YMIMVKCWMI	low
MHC_A_68_02	TLKYPIEHGI	low
MHC_A_68_02	FMDGKQACVL	low
MHC_A_68_02	SLYASSPGGV	low
MHC_A_68_02	EMREQHDAQV	low
MHC_A_68_02	TLRNTNPNFV	low
MHC_A_68_02	EMRQKHSQAV	low
MHC_A_68_02	RMFRWLVLRI	low
MHC_A_68_02	SYMMDDLELI	low
MHC_A_68_02	NMDWRSLTQV	low
MHC_A_68_02	NLYRIGQSKV	low
MHC_A_68_02	HLSWEWNLSI	low
MHC_A_68_02	NLPIYSEEIV	low
MHC_A_68_02	LLNVKMALDI	low
MHC_A_68_02	ELRLLIHQSL	low
MHC_A_68_02	VLKEGSEYRV	low
MHC_A_68_02	ALRTDYNASV	low
MHC_A_68_02	ELRENTQTTI	low
MHC_A_68_02	DLNSFEQLCI	low

MHC_A_68_02	ILDLISESPI	low
MHC_A_68_02	MMQDREDQSI	low
MHC_A_68_02	ILHVDNHIGI	low
MHC_A_68_02	WLVLRINKAL	low
MHC_A_68_02	NLKERYYSGL	low
MHC_A_68_02	ELFARSSDPR	low
MHC_A_68_02	PLNDNIATLL	low
MHC_A_68_02	NMKEEMARHL	low
MHC_A_68_02	DLTDYLMKIL	low
MHC_A_68_02	YLQLVFGIEV	low
MHC_A_68_02	ELEQTYHAKL	low
MHC_A_68_02	LAPPQHLIRV	low
MHC_A_68_02	VLYGPDAPTI	low
MHC_A_68_02	VMAGVGSPYV	low
MHC_A_68_02	KLKNKHEAMI	low
MHC_A_68_02	ELWKDVDRII	low
MHC_A_68_02	GLSQRHEEKV	low
MHC_A_68_02	QLKDKADFCI	low
MHC_A_68_02	RLFTKVKPLL	low
MHC_A_68_02	NLNELVKHGL	low
MHC_A_68_02	HMSEFMECNL	low
MHC_A_68_02	SANMDWRSLT	low
MHC_A_68_02	KMNPPKFSKV	low
MHC_A_68_02	ELVKHGLRAL	low
MHC_A_68_02	PLPNFSSLNL	low
MHC_A_68_02	NLQKESRACL	low
MHC_A_68_02	ELNGKNIEDV	low
MHC_A_68_02	IVDCLTEMYY	low
MHC_A_68_02	KLLEGEEERL	low
MHC_A_68_02	PLSPTRLSRL	low
MHC_A_68_02	KLRRGDLPFV	low
MHC_A_68_02	FMECNLNELV	low
MHC_A_68_02	RLPGPSDTPI	low
MHC_A_68_02	AMKAQFERDL	low
MHC_A_68_02	DMEISAYRKL	low
MHC_A_68_02	GLDLQPCIDL	low
MHC_A_68_02	KLRNWQWWRL	low
MHC_A_68_02	VLDQLRCNGV	low
MHC_A_68_02	VLQRNCAAYL	low
MHC_A_68_02	YADSVKGRFT	low
MHC_A_68_02	LLQSKNAGAV	low
MHC_A_68_02	PLEGSEDRII	low
MHC_A_68_02	PLESDAVECL	low

MHC_A_68_02	GLKSKTHAVL	low
MHC_A_68_02	PLPVSRLVSL	low
MHC_A_68_02	QAPGKGLEWV	low
MHC_A_68_02	QMNSLRAEDT	low
MHC_A_68_02	RLSCAASGFT	low
MHC_A_68_02	YLLSGAGEHL	low
MHC_A_68_02	EVQLVESGGGL	low
MHC_A_68_02	QLVFGIELMEV	low
MHC_A_68_02	SLLERGQQLGV	low
MHC_A_68_02	LLPENNVLSPL	low
MHC_A_68_02	YFYYNAFHWAI	low
MHC_A_68_02	RAEDTAVYYCA	low
MHC_A_68_02	ITLEDSSGNLL	low
MHC_A_68_02	IMPKAGLLIIV	low
MHC_A_68_02	FLLPILSQIYT	low
MHC_A_68_02	GSSDFQVHFLK	low
MHC_A_68_02	LEYFQFVKKLL	low
MHC_A_68_02	LPSCPTNFCIF	low
MHC_A_68_02	MIKYCLLKILK	low
MHC_A_68_02	MVRVLTVIKEY	low
MHC_A_68_02	RMILPMSRAFR	low
MHC_A_68_02	TFVPIAWAAAY	low
MHC_A_68_02	VALFSSCPVAY	low
MHC_A_68_02	YSLEYFQFVKK	low
MHC_A_68_02	YADSVKGRFTI	low
MHC_A_68_02	LQMNSLRAEDT	low
MHC_A_68_02	QMNSLRAEDTA	low
MHC_A_68_02	RLPGPSDTPIL	low
MHC_A_68_02	RQAPGKGLEWV	low
MHC_A_68_02	VQLVESGGGLV	low
MHC_A_68_23	MPMKGRFPI	low
MHC_A_69_01	ETFNTPAMY	low
MHC_A_69_01	YTSDYFISY	low
MHC_A_69_01	YTYPCIPEY	low
MHC_A_69_01	YTAVVPLVY	low
MHC_A_69_01	KSDLQPPNY	low
MHC_A_69_01	IPYHIVNIV	low
MHC_A_69_01	YVDHYYRDY	low
MHC_A_69_01	FPEHIFPAL	low
MHC_A_69_01	GSEDRDLLY	low
MHC_A_69_01	EVDPIGHLY	low
MHC_A_69_01	LPRWPPPQL	low
MHC_A_69_01	EPEPHILLF	low

MHC_A_69_01	STDHIPILY	low
MHC_A_69_01	SPRYIFTML	low
MHC_A_69_01	MIEPRTLQY	low
MHC_A_69_01	YLDDPDLKY	low
MHC_A_69_01	AMFIGHATA	low
MHC_A_69_01	ATDFKFAMY	low
MHC_A_69_01	CHKGWGVSV	low
MHC_A_69_01	CSDDGFWSK	low
MHC_A_69_01	CSEYVKDIY	low
MHC_A_69_01	DFDGTPRLY	low
MHC_A_69_01	DPSGAYFAW	low
MHC_A_69_01	EADPTGHSY	low
MHC_A_69_01	FAAPHRGVA	low
MHC_A_69_01	FHMDPSGTF	low
MHC_A_69_01	FLFDRLTNG	low
MHC_A_69_01	FLHSGTAKS	low
MHC_A_69_01	FQWHEAMFL	low
MHC_A_69_01	FWAWSVLRV	low
MHC_A_69_01	GHLENNPAL	low
MHC_A_69_01	GLEAYIQGI	low
MHC_A_69_01	GLENGLNYI	low
MHC_A_69_01	GRNSFEVRV	low
MHC_A_69_01	GRPNCFQIV	low
MHC_A_69_01	GTEYRLTLY	low
MHC_A_69_01	GVRQFSGWM	low
MHC_A_69_01	IADMGHLKY	low
MHC_A_69_01	IIYVGCGER	low
MHC_A_69_01	ILGPPGSVY	low
MHC_A_69_01	IVDCLTEMY	low
MHC_A_69_01	IYDFYYLDY	low
MHC_A_69_01	KCDICTDEY	low
MHC_A_69_01	KVRDRNFQL	low
MHC_A_69_01	LMQCWQLLA	low
MHC_A_69_01	LQDDFDFNY	low
MHC_A_69_01	LVDENQSWY	low
MHC_A_69_01	LVYNHCEHG	low
MHC_A_69_01	MCFHQHLMY	low
MHC_A_69_01	NPNSPSITY	low
MHC_A_69_01	NQRETTVVW	low
MHC_A_69_01	NRRFVNVVP	low
MHC_A_69_01	RPNRQLGSM	low
MHC_A_69_01	RPRHQGVMV	low
MHC_A_69_01	RRFFPYYVY	low

MHC_A_69_01	RRHWGGNVL	low
MHC_A_69_01	RTDNGGWAH	low
MHC_A_69_01	RVYNNTARY	low
MHC_A_69_01	RYFSVTRPL	low
MHC_A_69_01	SAEVVTLWY	low
MHC_A_69_01	SLFNWLWYE	low
MHC_A_69_01	SQFGGGSQY	low
MHC_A_69_01	SRDKTIIMW	low
MHC_A_69_01	SVDSDHLGY	low
MHC_A_69_01	TLKDGDFIL	low
MHC_A_69_01	VGYVDDTQF	low
MHC_A_69_01	VLIRRCHYL	low
MHC_A_69_01	VLWKSYPLV	low
MHC_A_69_01	WSTIWRQLY	low
MHC_A_69_01	WVGRASDPD	low
MHC_A_69_01	YGDTEAICR	low
MHC_A_69_01	YHEDIHTYL	low
MHC_A_69_01	YITDYSNDI	low
MHC_A_69_01	YLDFGGPEG	low
MHC_A_69_01	YLGSWATGK	low
MHC_A_69_01	YPAEITLTW	low
MHC_A_69_01	YQGSYGFRL	low
MHC_A_69_01	YSDNEMLTH	low
MHC_A_69_01	YTFCGTIEY	low
MHC_A_69_01	DSDGSFFLY	low
MHC_A_69_01	YFDPANGKF	low
MHC_A_80_01	TFMDHVLRY	low
MHC_A_80_01	YYSNKAYQY	low
MHC_A_80_01	YTSDYFISY	low
MHC_A_80_01	ETFNTPAMY	low
MHC_A_80_01	NYIDKVRFL	low
MHC_A_80_01	YYWPRPRRY	low
MHC_A_80_01	AMFIGHATA	low
MHC_A_80_01	FAAPHRGVA	low
MHC_A_80_01	FFGETSHNY	low
MHC_A_80_01	FLFDRLTNG	low
MHC_A_80_01	FWAWSVLRV	low
MHC_A_80_01	GLEAYIQGI	low
MHC_A_80_01	GSDCTTIHY	low
MHC_A_80_01	GTEYRLTLY	low
MHC_A_80_01	GVRQFSGWM	low
MHC_A_80_01	GYLHDFLKY	low
MHC_A_80_01	KVRDRNFQL	low

MHC_A_80_01	LMQCWQLLA	low
MHC_A_80_01	LQDDFDFNY	low
MHC_A_80_01	MQDVFTFYV	low
MHC_A_80_01	NTDDFPLTL	low
MHC_A_80_01	RPNRQLGSM	low
MHC_A_80_01	RTDNGGWAH	low
MHC_A_80_01	SAEVVTLWY	low
MHC_A_80_01	SLFNWLWYE	low
MHC_A_80_01	TLKDGDFIL	low
MHC_A_80_01	VLIRRCHYL	low
MHC_A_80_01	VVPSYIPLV	low
MHC_A_80_01	WTDLFDNKV	low
MHC_A_80_01	WVDSTPPPG	low
MHC_A_80_01	YITDYSNDI	low
MHC_A_80_01	YSDNEMLTH	low
MHC_A_80_01	YYFSYPLFV	low
MHC_A_80_01	YYRYPTGESY	low
MHC_A_80_01	YFEYIEENKY	low
MHC_B_07_02	FYYNAFHW	low
MHC_B_07_02	YYNAFHWA	low
MHC_B_07_02	IPIGTYGQM	low
MHC_B_07_02	RPRHQGVMV	low
MHC_B_07_02	IPLYRNGDF	low
MHC_B_07_02	CPLERFAEL	low
MHC_B_07_02	LPSIPVHPI	low
MHC_B_07_02	FPHTELANL	low
MHC_B_07_02	YPAEITLTW	low
MHC_B_07_02	LPRWPPPQL	low
MHC_B_07_02	MPREDAHFI	low
MHC_B_07_02	VPIAWAAAY	low
MHC_B_07_02	SPRYIFTML	low
MHC_B_07_02	IPGFKFDNL	low
MHC_B_07_02	AHAGAKFPI	low
MHC_B_07_02	SPSCPLERF	low
MHC_B_07_02	FPEHIFPAL	low
MHC_B_07_02	QPRYPVNSV	low
MHC_B_07_02	LPKEACMEI	low
MHC_B_07_02	LPFRNCPRF	low
MHC_B_07_02	APGKGLEWV	low
MHC_B_07_02	IPYHIVNIV	low
MHC_B_07_02	TPMFNDINI	low
MHC_B_07_02	EPEPHILLF	low
MHC_B_07_02	VGYVDDTQF	low

MHC_B_07_02	NPNSPSITY	low
MHC_B_07_02	HPEDTGQVF	low
MHC_B_07_02	GPQFPFTGV	low
MHC_B_07_02	DPSGAYFAW	low
MHC_B_07_02	ATDFKFAMY	low
MHC_B_07_02	CHKGWGVSV	low
MHC_B_07_02	CSDDGFWSK	low
MHC_B_07_02	CSEYVKDIY	low
MHC_B_07_02	DFDGTPRLY	low
MHC_B_07_02	DSDGSFFLY	low
MHC_B_07_02	EADPTGHSY	low
MHC_B_07_02	ETFNTPAMY	low
MHC_B_07_02	EVDPIGHLY	low
MHC_B_07_02	FHMDPSGTF	low
MHC_B_07_02	FQILHDRFF	low
MHC_B_07_02	FYYNAFHWA	low
MHC_B_07_02	GHLENNPAL	low
MHC_B_07_02	GLENGLNYI	low
MHC_B_07_02	GRNSFEVRV	low
MHC_B_07_02	GRPNCFQIV	low
MHC_B_07_02	GSEDRDLLY	low
MHC_B_07_02	IADMGHLKY	low
MHC_B_07_02	IVDCLTEMY	low
MHC_B_07_02	KCDICTDEY	low
MHC_B_07_02	KSDLQPPNY	low
MHC_B_07_02	LVYNHCEHG	low
MHC_B_07_02	MCFHQHLMY	low
MHC_B_07_02	MIEPRTLQY	low
MHC_B_07_02	NQRETTVVW	low
MHC_B_07_02	NRRFVNVVP	low
MHC_B_07_02	RRFFPYYVY	low
MHC_B_07_02	RRHWGGNVL	low
MHC_B_07_02	RVYNNTARY	low
MHC_B_07_02	SQFGGGSQY	low
MHC_B_07_02	SRDKTIIMW	low
MHC_B_07_02	SSKMFNYFK	low
MHC_B_07_02	STDHIPILY	low
MHC_B_07_02	TEAEKWPFF	low
MHC_B_07_02	WVGRASDPD	low
MHC_B_07_02	YFDPANGKF	low
MHC_B_07_02	YFYYNAFHW	low
MHC_B_07_02	YGDTEAICR	low
MHC_B_07_02	YHEDIHTYL	low

MHC_B_07_02	YLDDPDLKY	low
MHC_B_07_02	YLDFGGPEG	low
MHC_B_07_02	YLGSWATGK	low
MHC_B_07_02	YTSDYFISY	low
MHC_B_07_02	YTYPCIPEY	low
MHC_B_07_02	YVDHYYRDY	low
MHC_B_07_02	YYNAFHWAI	low
MHC_B_07_02	DPIFLLHHA	low
MHC_B_07_02	FPPEGVSIW	low
MHC_B_07_02	RYFSVTRPL	low
MHC_B_07_02	ILGPPGSVY	low
MHC_B_07_02	YTAVVPLVY	low
MHC_B_07_02	FRYNGLIHR	low
MHC_B_07_02	MPREDAHFIY	low
MHC_B_07_02	GPRTAALGLL	low
MHC_B_07_02	MPACTLELPT	low
MHC_B_07_02	RLIPIKYKAM	low
MHC_B_07_02	RPHGDLPIYV	low
MHC_B_07_02	NPNQNKNVAL	low
MHC_B_07_02	SPGAHQKRLI	low
MHC_B_07_02	LPGPSDTPIL	low
MHC_B_07_02	ETFGFEIQSY	low
MHC_B_07_02	ELFARSSDPR	low
MHC_B_07_02	FYYNAFHWAI	low
MHC_B_07_02	IVDCLTEMYY	low
MHC_B_07_02	SRDSRGKPGY	low
MHC_B_07_02	SYMMDDLELI	low
MHC_B_07_02	PLALEGSLQK	low
MHC_B_07_02	STTAICATGL	low
MHC_B_07_02	GPGAGSLQPLA	low
MHC_B_07_02	LPSCPTNFCIF	low
MHC_B_07_02	SPNQARAQAAL	low
MHC_B_07_02	QPGGSLRLSCA	low
MHC_B_07_02	FLLPILSQIYT	low
MHC_B_07_02	GSSDFQVHFLK	low
MHC_B_07_02	LEYFQFVKKLL	low
MHC_B_07_02	MIKYCLLKILK	low
MHC_B_07_02	MVRVLTVIKEY	low
MHC_B_07_02	RMILPMSRAFR	low
MHC_B_07_02	SLLERGQQLGV	low
MHC_B_07_02	TFVPIAWAAAY	low
MHC_B_07_02	VALFSSCPVAY	low
MHC_B_07_02	YFYYNAFHWAI	low

MHC_B_07_02	YSLEYFQFVKK	low
MHC_B_07_02	GPGAGSLQPLAL	low
MHC_B_08_01	FPHTELANL	low
MHC_B_08_01	YTAVVPLVY	low
MHC_B_08_01	FQILHDRFF	low
MHC_B_08_01	FRHSVVVPY	low
MHC_B_08_01	LQIRGRERF	low
MHC_B_08_01	RRCPHHERC	low
MHC_B_08_01	RVYNNTARY	low
MHC_B_08_01	SSKMFNYFK	low
MHC_B_08_01	TEAEKWPFF	low
MHC_B_08_01	VRVCACPGR	low
MHC_B_08_01	DTAVYYCAR	low
MHC_B_08_01	FTISRDNSK	low
MHC_B_08_01	FTFSSYGMH	low
MHC_B_08_01	LRAEDTAVY	low
MHC_B_08_01	RAEDTAVYY	low
MHC_B_08_01	SSYGMHWVR	low
MHC_B_08_01	VRQAPGKGL	low
MHC_B_08_01	FRYNGLIHR	low
MHC_B_08_01	ISRDNSKNTL	low
MHC_B_08_01	TLYLQMNSLR	low
MHC_B_08_01	SVKGRFTISR	low
MHC_B_08_01	WVRQAPGKGL	low
MHC_B_08_01	LRLSCAASGF	low
MHC_B_08_01	EKRCRRMVVV	low
MHC_B_08_01	ELFARSSDPR	low
MHC_B_08_01	IVDCLTEMYY	low
MHC_B_08_01	SYMMDDLELI	low
MHC_B_08_01	AVIWYDGSNK	low
MHC_B_08_01	ETFGFEIQSY	low
MHC_B_08_01	FSSYGMHWVR	low
MHC_B_08_01	GLEWVAVIWY	low
MHC_B_08_01	IWYDGSNKYY	low
MHC_B_08_01	LRAEDTAVYY	low
MHC_B_08_01	LEYFQFVKKLL	low
MHC_B_08_01	FLLPILSQIYT	low
MHC_B_08_01	GSSDFQVHFLK	low
MHC_B_08_01	LPSCPTNFCIF	low
MHC_B_08_01	MIKYCLLKILK	low
MHC_B_08_01	MVRVLTVIKEY	low
MHC_B_08_01	RMILPMSRAFR	low
MHC_B_08_01	SLLERGQQLGV	low

MHC_B_08_01	TFVPIAWAAAY	low
MHC_B_08_01	VALFSSCPVAY	low
MHC_B_08_01	YFYYNAFHWAI	low
MHC_B_08_01	YSLEYFQFVKK	low
MHC_B_08_02	FRHSVVVPY	low
MHC_B_08_02	LQIRGRERF	low
MHC_B_08_02	RRCPHHERC	low
MHC_B_08_02	RVYNNTARY	low
MHC_B_08_02	VRVCACPGR	low
MHC_B_08_03	LQIRGRERF	low
MHC_B_08_03	RRCPHHERC	low
MHC_B_08_03	RVYNNTARY	low
MHC_B_08_03	VRVCACPGR	low
MHC_B_14_02	FQILHDRFF	low
MHC_B_14_02	FPHTELANL	low
MHC_B_14_02	SSKMFNYFK	low
MHC_B_14_02	TEAEKWPFF	low
MHC_B_14_02	FRYNGLIHR	low
MHC_B_14_02	YTAVVPLVY	low
MHC_B_14_02	ELFARSSDPR	low
MHC_B_14_02	IVDCLTEMYY	low
MHC_B_14_02	SYMMDDLELI	low
MHC_B_14_02	ETFGFEIQSY	low
MHC_B_14_02	YFYYNAFHWAI	low
MHC_B_14_02	FLLPILSQIYT	low
MHC_B_14_02	GSSDFQVHFLK	low
MHC_B_14_02	LEYFQFVKKLL	low
MHC_B_14_02	LPSCPTNFCIF	low
MHC_B_14_02	MIKYCLLKILK	low
MHC_B_14_02	MVRVLTVIKEY	low
MHC_B_14_02	RMILPMSRAFR	low
MHC_B_14_02	SLLERGQQLGV	low
MHC_B_14_02	TFVPIAWAAAY	low
MHC_B_14_02	VALFSSCPVAY	low
MHC_B_14_02	YSLEYFQFVKK	low
MHC_B_15_01	RGPDAFRF	low
MHC_B_15_01	SMHFYGWSL	low
MHC_B_15_01	SFAEIHQAF	low
MHC_B_15_01	YTAVVPLVY	low
MHC_B_15_01	ITHTGEKPY	low
MHC_B_15_01	YTYPCIPEY	low
MHC_B_15_01	NQRETTVVW	low
MHC_B_15_01	YTSDYFISY	low

MHC_B_15_01	MIEPRTLQY	low
MHC_B_15_01	RVYNNTARY	low
MHC_B_15_01	MLRVYNNTA	low
MHC_B_15_01	FRHSVVVPY	low
MHC_B_15_01	YLDDPDLKY	low
MHC_B_15_01	FHMDPSGTF	low
MHC_B_15_01	ETFNTPAMY	low
MHC_B_15_01	VGYVDDTQF	low
MHC_B_15_01	IVDCLTEMY	low
MHC_B_15_01	MCFHQHLMY	low
MHC_B_15_01	EVDPIGHLY	low
MHC_B_15_01	IADMGHLKY	low
MHC_B_15_01	ATDFKFAMY	low
MHC_B_15_01	NPNSPSITY	low
MHC_B_15_01	SGKLKVPEW	low
MHC_B_15_01	EADPTGHSY	low
MHC_B_15_01	RRFFPYYVY	low
MHC_B_15_01	CHKGWGVSV	low
MHC_B_15_01	CSDDGFWSK	low
MHC_B_15_01	CSEYVKDIY	low
MHC_B_15_01	DFDGTPRLY	low
MHC_B_15_01	DPSGAYFAW	low
MHC_B_15_01	DSDGSFFLY	low
MHC_B_15_01	EPEPHILLF	low
MHC_B_15_01	FAAPHRGVA	low
MHC_B_15_01	FLFDRLTNG	low
MHC_B_15_01	FPEHIFPAL	low
MHC_B_15_01	FWAWSVLRV	low
MHC_B_15_01	GHLENNPAL	low
MHC_B_15_01	GLEAYIQGI	low
MHC_B_15_01	GLENGLNYI	low
MHC_B_15_01	GRNSFEVRV	low
MHC_B_15_01	GRPNCFQIV	low
MHC_B_15_01	GSEDRDLLY	low
MHC_B_15_01	GTEYRLTLY	low
MHC_B_15_01	GVRQFSGWM	low
MHC_B_15_01	IIYVGCGER	low
MHC_B_15_01	IPYHIVNIV	low
MHC_B_15_01	IYDFYYLDY	low
MHC_B_15_01	KCDICTDEY	low
MHC_B_15_01	KSDLQPPNY	low
MHC_B_15_01	KVRDRNFQL	low
MHC_B_15_01	LMQCWQLLA	low

MHC_B_15_01	LPRWPPPQL	low
MHC_B_15_01	LQDDFDFNY	low
MHC_B_15_01	LVYNHCEHG	low
MHC_B_15_01	MQDVFTFYV	low
MHC_B_15_01	NRRFVNVVP	low
MHC_B_15_01	NTDDFPLTL	low
MHC_B_15_01	RPNRQLGSM	low
MHC_B_15_01	RPRHQGVMV	low
MHC_B_15_01	RRHWGGNVL	low
MHC_B_15_01	RTDNGGWAH	low
MHC_B_15_01	RYFSVTRPL	low
MHC_B_15_01	SAEVVTLWY	low
MHC_B_15_01	SLFNWLWYE	low
MHC_B_15_01	SPRYIFTML	low
MHC_B_15_01	SRDKTIIMW	low
MHC_B_15_01	STDHIPILY	low
MHC_B_15_01	VLIRRCHYL	low
MHC_B_15_01	VLWKSYPLV	low
MHC_B_15_01	VVPSYIPLV	low
MHC_B_15_01	WTDLFDNKV	low
MHC_B_15_01	WVGRASDPD	low
MHC_B_15_01	YFDPANGKF	low
MHC_B_15_01	YGDTEAICR	low
MHC_B_15_01	YHEDIHTYL	low
MHC_B_15_01	YITDYSNDI	low
MHC_B_15_01	YLDFGGPEG	low
MHC_B_15_01	YLGSWATGK	low
MHC_B_15_01	YPAEITLTW	low
MHC_B_15_01	YSDNEMLTH	low
MHC_B_15_01	YVDHYYRDY	low
MHC_B_15_01	YYFSYPLFV	low
MHC_B_15_01	AGVWSQDKW	low
MHC_B_15_01	FRYNGLIHR	low
MHC_B_15_01	RGVEKPPHL	low
MHC_B_15_01	ETFGFEIQSY	low
MHC_B_15_01	QITHTGEKPY	low
MHC_B_15_01	KGFSEEHNTW	low
MHC_B_15_01	KGFTDADNTW	low
MHC_B_15_01	SGGPKYEYRW	low
MHC_B_15_01	SRDSRGKPGY	low
MHC_B_15_03	YTAVVPLVY	low
MHC_B_15_03	FRYNGLIHR	low
MHC_B_15_03	ETFGFEIQSY	low

MHC_B_15_09	FRHSVVVPY	low
MHC_B_15_09	LQIRGRERF	low
MHC_B_15_09	RRCPHHERC	low
MHC_B_15_09	VRVCACPGR	low
MHC_B_15_17	YSPALNKMF	low
MHC_B_15_17	LSPDDIEQW	low
MHC_B_15_17	AMFIGHATA	low
MHC_B_15_17	FAAPHRGVA	low
MHC_B_15_17	FLFDRLTNG	low
MHC_B_15_17	FQWHEAMFL	low
MHC_B_15_17	FWAWSVLRV	low
MHC_B_15_17	GLEAYIQGI	low
MHC_B_15_17	GVRQFSGWM	low
MHC_B_15_17	IYDFYYLDY	low
MHC_B_15_17	LMQCWQLLA	low
MHC_B_15_17	LQDDFDFNY	low
MHC_B_15_17	LQIRGRERF	low
MHC_B_15_17	MQDVFTFYV	low
MHC_B_15_17	NTDDFPLTL	low
MHC_B_15_17	RPNRQLGSM	low
MHC_B_15_17	RTDNGGWAH	low
MHC_B_15_17	SLFNWLWYE	low
MHC_B_15_17	TLKDGDFIL	low
MHC_B_15_17	VLIRRCHYL	low
MHC_B_15_17	VLWKSYPLV	low
MHC_B_15_17	WTDLFDNKV	low
MHC_B_15_17	YITDYSNDI	low
MHC_B_15_17	YSDNEMLTH	low
MHC_B_15_42	FEYGGFPPA	low
MHC_B_15_42	FRMLAWHVL	low
MHC_B_15_42	MPMKGRFPI	low
MHC_B_15_42	RRHWGGNVL	low
MHC_B_15_42	SILPISWAY	low
MHC_B_15_42	SVDSDHLGY	low
MHC_B_15_42	TFMDHVLRY	low
MHC_B_15_42	YEHYFVFAA	low
MHC_B_15_42	YYWPRPRRY	low
MHC_B_15_42	YYRYPTGESY	low
MHC_B_18_01	FPHTELANL	low
MHC_B_18_01	FRHSVVVPY	low
MHC_B_18_01	YTAVVPLVY	low
MHC_B_18_01	FQILHDRFF	low
MHC_B_18_01	FRYNGLIHR	low

MHC_B_18_01	AMFIGHATA	low
MHC_B_18_01	FAAPHRGVA	low
MHC_B_18_01	FLFDRLTNG	low
MHC_B_18_01	FWAWSVLRV	low
MHC_B_18_01	GLEAYIQGI	low
MHC_B_18_01	GVRQFSGWM	low
MHC_B_18_01	IIYVGCGER	low
MHC_B_18_01	IYDFYYLDY	low
MHC_B_18_01	KVRDRNFQL	low
MHC_B_18_01	LMQCWQLLA	low
MHC_B_18_01	LQDDFDFNY	low
MHC_B_18_01	LVDENQSWY	low
MHC_B_18_01	MQDVFTFYV	low
MHC_B_18_01	NTDDFPLTL	low
MHC_B_18_01	RPNRQLGSM	low
MHC_B_18_01	RTDNGGWAH	low
MHC_B_18_01	RVYNNTARY	low
MHC_B_18_01	SAEVVTLWY	low
MHC_B_18_01	SLFNWLWYE	low
MHC_B_18_01	SSKMFNYFK	low
MHC_B_18_01	TLKDGDFIL	low
MHC_B_18_01	VLIRRCHYL	low
MHC_B_18_01	VVPSYIPLV	low
MHC_B_18_01	WTDLFDNKV	low
MHC_B_18_01	YITDYSNDI	low
MHC_B_18_01	YSDNEMLTH	low
MHC_B_18_01	YYFSYPLFV	low
MHC_B_18_01	ETFGFEIQSY	low
MHC_B_18_01	IVDCLTEMYY	low
MHC_B_18_01	EEGNLLDSYF	low
MHC_B_18_01	ELFARSSDPR	low
MHC_B_18_01	SYMMDDLELI	low
MHC_B_18_01	TFVPIAWAAAY	low
MHC_B_18_01	MVRVLTVIKEY	low
MHC_B_18_01	LEYFQFVKKLL	low
MHC_B_18_01	FLLPILSQIYT	low
MHC_B_18_01	GSSDFQVHFLK	low
MHC_B_18_01	LPSCPTNFCIF	low
MHC_B_18_01	MIKYCLLKILK	low
MHC_B_18_01	RMILPMSRAFR	low
MHC_B_18_01	SLLERGQQLGV	low
MHC_B_18_01	VALFSSCPVAY	low
MHC_B_18_01	YFYYNAFHWAI	low

MHC_B_18_01	YSLEYFQFVKK	low
MHC_B_27_01	RRFFPYYV	low
MHC_B_27_01	RRFFPYYVY	low
MHC_B_27_02	RRFFPYYV	low
MHC_B_27_02	RRFFPYYVY	low
MHC_B_27_02	RRKSSGGKGGSY	low
MHC_B_27_03	RRFFPYYV	low
MHC_B_27_03	RRFFPYYVY	low
MHC_B_27_03	AMFIGHATA	low
MHC_B_27_03	FAAPHRGVA	low
MHC_B_27_03	FLFDRLTNG	low
MHC_B_27_03	FQWHEAMFL	low
MHC_B_27_03	FWAWSVLRV	low
MHC_B_27_03	GLEAYIQGI	low
MHC_B_27_03	GTEYRLTLY	low
MHC_B_27_03	GVRQFSGWM	low
MHC_B_27_03	IIYVGCGER	low
MHC_B_27_03	IYDFYYLDY	low
MHC_B_27_03	KVRDRNFQL	low
MHC_B_27_03	LMQCWQLLA	low
MHC_B_27_03	LQDDFDFNY	low
MHC_B_27_03	LVDENQSWY	low
MHC_B_27_03	MQDVFTFYV	low
MHC_B_27_03	NTDDFPLTL	low
MHC_B_27_03	NTFKFGVIY	low
MHC_B_27_03	RPNRQLGSM	low
MHC_B_27_03	RTDNGGWAH	low
MHC_B_27_03	SAEVVTLWY	low
MHC_B_27_03	SILPISWAY	low
MHC_B_27_03	SLFNWLWYE	low
MHC_B_27_03	SVDSDHLGY	low
MHC_B_27_03	TLKDGDFIL	low
MHC_B_27_03	VLIRRCHYL	low
MHC_B_27_03	VLWKSYPLV	low
MHC_B_27_03	VVPSYIPLV	low
MHC_B_27_03	WSTIWRQLY	low
MHC_B_27_03	WTDLFDNKV	low
MHC_B_27_03	YITDYSNDI	low
MHC_B_27_03	YSDNEMLTH	low
MHC_B_27_03	YTFCGTIEY	low
MHC_B_27_03	YYFSYPLFV	low
MHC_B_27_04	RRFFPYYV	low
MHC_B_27_04	RRFFPYYVY	low

MHC_B_27_04	RRKSSGGKGGSY	low
MHC_B_27_05	RWRGWYTY	low
MHC_B_27_05	RRFFPYYV	low
MHC_B_27_05	SRRFFPYY	low
MHC_B_27_05	LRAEDTAVY	low
MHC_B_27_05	VRQAPGKGL	low
MHC_B_27_05	SSYGMHWVR	low
MHC_B_27_05	FIVNTNVPR	low
MHC_B_27_05	RRFFPYYVY	low
MHC_B_27_05	LQIRGRERF	low
MHC_B_27_05	GRNSFEVRV	low
MHC_B_27_05	SRRFFPYYV	low
MHC_B_27_05	RRHWGGNVL	low
MHC_B_27_05	AVFPSIVGR	low
MHC_B_27_05	AGLQFPVGR	low
MHC_B_27_05	SQFGGGSQY	low
MHC_B_27_05	RYFSVTRPL	low
MHC_B_27_05	FTFSSYGMH	low
MHC_B_27_05	ATDFKFAMY	low
MHC_B_27_05	CHKGWGVSV	low
MHC_B_27_05	CSDDGFWSK	low
MHC_B_27_05	CSEYVKDIY	low
MHC_B_27_05	DFDGTPRLY	low
MHC_B_27_05	DPSGAYFAW	low
MHC_B_27_05	DSDGSFFLY	low
MHC_B_27_05	EADPTGHSY	low
MHC_B_27_05	EPEPHILLF	low
MHC_B_27_05	ETFNTPAMY	low
MHC_B_27_05	EVDPIGHLY	low
MHC_B_27_05	FHMDPSGTF	low
MHC_B_27_05	FPEHIFPAL	low
MHC_B_27_05	GHLENNPAL	low
MHC_B_27_05	GLENGLNYI	low
MHC_B_27_05	GRPNCFQIV	low
MHC_B_27_05	GSEDRDLLY	low
MHC_B_27_05	IADMGHLKY	low
MHC_B_27_05	ILGPPGSVY	low
MHC_B_27_05	IPYHIVNIV	low
MHC_B_27_05	IVDCLTEMY	low
MHC_B_27_05	KCDICTDEY	low
MHC_B_27_05	KSDLQPPNY	low
MHC_B_27_05	LPRWPPPQL	low
MHC_B_27_05	LVYNHCEHG	low

MHC_B_27_05	MCFHQHLMY	low
MHC_B_27_05	MIEPRTLQY	low
MHC_B_27_05	NPNSPSITY	low
MHC_B_27_05	NQRETTVVW	low
MHC_B_27_05	NRRFVNVVP	low
MHC_B_27_05	RPRHQGVMV	low
MHC_B_27_05	RVYNNTARY	low
MHC_B_27_05	SPRYIFTML	low
MHC_B_27_05	SRDKTIIMW	low
MHC_B_27_05	STDHIPILY	low
MHC_B_27_05	VGYVDDTQF	low
MHC_B_27_05	WVGRASDPD	low
MHC_B_27_05	YFDPANGKF	low
MHC_B_27_05	YGDTEAICR	low
MHC_B_27_05	YHEDIHTYL	low
MHC_B_27_05	YLDDPDLKY	low
MHC_B_27_05	YLDFGGPEG	low
MHC_B_27_05	YLGSWATGK	low
MHC_B_27_05	YPAEITLTW	low
MHC_B_27_05	YTSDYFISY	low
MHC_B_27_05	YTYPCIPEY	low
MHC_B_27_05	YVDHYYRDY	low
MHC_B_27_05	RAEDTAVYY	low
MHC_B_27_05	FTISRDNSK	low
MHC_B_27_05	DTAVYYCAR	low
MHC_B_27_05	TLYLQMNSL	low
MHC_B_27_05	YTAVVPLVY	low
MHC_B_27_05	SRDSRGKPGY	low
MHC_B_27_05	WVRQAPGKGL	low
MHC_B_27_05	LRAEDTAVYY	low
MHC_B_27_05	RRFFPYYVYN	low
MHC_B_27_05	SLPANVPTPR	low
MHC_B_27_05	ISRDNSKNTL	low
MHC_B_27_05	FSSYGMHWVR	low
MHC_B_27_05	SRRFFPYYVY	low
MHC_B_27_05	SVKGRFTISR	low
MHC_B_27_05	AVIWYDGSNK	low
MHC_B_27_05	DIDSPPITAR	low
MHC_B_27_05	TLYLQMNSLR	low
MHC_B_27_05	ETFGFEIQSY	low
MHC_B_27_05	GLEWVAVIWY	low
MHC_B_27_05	IWYDGSNKYY	low
MHC_B_27_05	RRFFPYYVYNI	low

MHC_B_27_05	SRRFFPYYVYN	low
MHC_B_27_05	RRKSSGGKGGSY	low
MHC_B_27_06	RRFFPYYV	low
MHC_B_27_06	RRFFPYYVY	low
MHC_B_27_06	RRKSSGGKGGSY	low
MHC_B_27_10	RRFFPYYV	low
MHC_B_27_10	RRFFPYYVY	low
MHC_B_35_01	CPLERFAEL	low
MHC_B_35_01	YTAVVPLVY	low
MHC_B_35_01	RVYNNTARY	low
MHC_B_35_01	HPEDTGQVF	low
MHC_B_35_01	SPSCPLERF	low
MHC_B_35_01	LPFRNCPRF	low
MHC_B_35_01	MPREDAHFI	low
MHC_B_35_01	FQILHDRFF	low
MHC_B_35_01	HPQKVTKFM	low
MHC_B_35_01	LPSIPVHPI	low
MHC_B_35_01	FPPEGVSIW	low
MHC_B_35_01	SSKMFNYFK	low
MHC_B_35_01	TEAEKWPFF	low
MHC_B_35_01	DPIFLLHHA	low
MHC_B_35_01	GPQFPFTGV	low
MHC_B_35_01	IPLYRNGDF	low
MHC_B_35_01	LPKEACMEI	low
MHC_B_35_01	TPMFNDINI	low
MHC_B_35_01	APGKGLEWV	low
MHC_B_35_01	FRYNGLIHR	low
MHC_B_35_01	ETFGFEIQSY	low
MHC_B_35_01	LPGPSDTPIL	low
MHC_B_35_01	ELFARSSDPR	low
MHC_B_35_01	SYMMDDLELI	low
MHC_B_35_01	PLALEGSLQK	low
MHC_B_35_01	FLLPILSQIYT	low
MHC_B_35_01	TFVPIAWAAAY	low
MHC_B_35_01	RMILPMSRAFR	low
MHC_B_35_01	GSSDFQVHFLK	low
MHC_B_35_01	LEYFQFVKKLL	low
MHC_B_35_01	MIKYCLLKILK	low
MHC_B_35_01	SLLERGQQLGV	low
MHC_B_35_01	YFYYNAFHWAI	low
MHC_B_35_01	YSLEYFQFVKK	low
MHC_B_35_01	QPGGSLRLSCA	low
MHC_B_35_01	GPGAGSLQPLAL	low

MHC_B_35_03	YTAVVPLVY	low
MHC_B_35_03	FRYNGLIHR	low
MHC_B_35_03	ETFGFEIQSY	low
MHC_B_35_03	PLALEGSLQK	low
MHC_B_35_03	GPGAGSLQPLAL	low
MHC_B_37_01	FQILHDRFF	low
MHC_B_37_01	TEAEKWPFF	low
MHC_B_37_01	FPHTELANL	low
MHC_B_37_01	SSKMFNYFK	low
MHC_B_37_01	YTAVVPLVY	low
MHC_B_37_01	ELFARSSDPR	low
MHC_B_37_01	IVDCLTEMYY	low
MHC_B_37_01	SYMMDDLELI	low
MHC_B_37_01	FLLPILSQIYT	low
MHC_B_37_01	GSSDFQVHFLK	low
MHC_B_37_01	LEYFQFVKKLL	low
MHC_B_37_01	LPSCPTNFCIF	low
MHC_B_37_01	MIKYCLLKILK	low
MHC_B_37_01	MVRVLTVIKEY	low
MHC_B_37_01	RMILPMSRAFR	low
MHC_B_37_01	SLLERGQQLGV	low
MHC_B_37_01	TFVPIAWAAAY	low
MHC_B_37_01	VALFSSCPVAY	low
MHC_B_37_01	YFYYNAFHWAI	low
MHC_B_37_01	YSLEYFQFVKK	low
MHC_B_38_01	YTAVVPLVY	low
MHC_B_38_01	FRYNGLIHR	low
MHC_B_39_01	AMFIGHATA	low
MHC_B_39_01	FAAPHRGVA	low
MHC_B_39_01	FLFDRLTNG	low
MHC_B_39_01	FRHSVVVPY	low
MHC_B_39_01	FWAWSVLRV	low
MHC_B_39_01	GLEAYIQGI	low
MHC_B_39_01	GTEYRLTLY	low
MHC_B_39_01	GVRQFSGWM	low
MHC_B_39_01	IIYVGCGER	low
MHC_B_39_01	IYDFYYLDY	low
MHC_B_39_01	KVRDRNFQL	low
MHC_B_39_01	LMQCWQLLA	low
MHC_B_39_01	LQDDFDFNY	low
MHC_B_39_01	LQIRGRERF	low
MHC_B_39_01	LVDENQSWY	low
MHC_B_39_01	NTFKFGVIY	low

MHC_B_39_01	RRCPHHERC	low
MHC_B_39_01	RTDNGGWAH	low
MHC_B_39_01	SAEVVTLWY	low
MHC_B_39_01	SILPISWAY	low
MHC_B_39_01	SLFNWLWYE	low
MHC_B_39_01	SVDSDHLGY	low
MHC_B_39_01	TLKDGDFIL	low
MHC_B_39_01	VLIRRCHYL	low
MHC_B_39_01	VLWKSYPLV	low
MHC_B_39_01	VRVCACPGR	low
MHC_B_39_01	VVPSYIPLV	low
MHC_B_39_01	WSTIWRQLY	low
MHC_B_39_01	WTDLFDNKV	low
MHC_B_39_01	YITDYSNDI	low
MHC_B_39_01	YSDNEMLTH	low
MHC_B_39_01	YTFCGTIEY	low
MHC_B_40_01	FPEHIFPAL	low
MHC_B_40_01	YHEDIHTYL	low
MHC_B_40_01	YTAVVPLVY	low
MHC_B_40_01	FRYNGLIHR	low
MHC_B_40_01	ATDFKFAMY	low
MHC_B_40_01	CHKGWGVSV	low
MHC_B_40_01	CSDDGFWSK	low
MHC_B_40_01	CSEYVKDIY	low
MHC_B_40_01	DFDGTPRLY	low
MHC_B_40_01	DPSGAYFAW	low
MHC_B_40_01	DSDGSFFLY	low
MHC_B_40_01	EADPTGHSY	low
MHC_B_40_01	EPEPHILLF	low
MHC_B_40_01	ETFNTPAMY	low
MHC_B_40_01	EVDPIGHLY	low
MHC_B_40_01	FHMDPSGTF	low
MHC_B_40_01	GHLENNPAL	low
MHC_B_40_01	GLENGLNYI	low
MHC_B_40_01	GRNSFEVRV	low
MHC_B_40_01	GRPNCFQIV	low
MHC_B_40_01	GSEDRDLLY	low
MHC_B_40_01	IADMGHLKY	low
MHC_B_40_01	ILGPPGSVY	low
MHC_B_40_01	IPYHIVNIV	low
MHC_B_40_01	IVDCLTEMY	low
MHC_B_40_01	KCDICTDEY	low
MHC_B_40_01	KSDLQPPNY	low

MHC_B_40_01	LPRWPPPQL	low
MHC_B_40_01	LVYNHCEHG	low
MHC_B_40_01	MCFHQHLMY	low
MHC_B_40_01	MIEPRTLQY	low
MHC_B_40_01	NPNSPSITY	low
MHC_B_40_01	NQRETTVVW	low
MHC_B_40_01	NRRFVNVVP	low
MHC_B_40_01	RPRHQGVMV	low
MHC_B_40_01	RRFFPYYVY	low
MHC_B_40_01	RVYNNTARY	low
MHC_B_40_01	RYFSVTRPL	low
MHC_B_40_01	SPRYIFTML	low
MHC_B_40_01	SQFGGGSQY	low
MHC_B_40_01	SRDKTIIMW	low
MHC_B_40_01	STDHIPILY	low
MHC_B_40_01	VGYVDDTQF	low
MHC_B_40_01	WVGRASDPD	low
MHC_B_40_01	YGDTEAICR	low
MHC_B_40_01	YLDDPDLKY	low
MHC_B_40_01	YLDFGGPEG	low
MHC_B_40_01	YLGSWATGK	low
MHC_B_40_01	YPAEITLTW	low
MHC_B_40_01	YTSDYFISY	low
MHC_B_40_01	YTYPCIPEY	low
MHC_B_40_01	YVDHYYRDY	low
MHC_B_40_01	RRHWGGNVL	low
MHC_B_40_01	ETFGFEIQSY	low
MHC_B_40_01	EEGNLLDSYF	low
MHC_B_40_02	FPHTELANL	low
MHC_B_40_02	FQILHDRFF	low
MHC_B_40_02	SSKMFNYFK	low
MHC_B_40_02	FRYNGLIHR	low
MHC_B_40_02	YTAVVPLVY	low
MHC_B_40_02	ETFGFEIQSY	low
MHC_B_40_02	IVDCLTEMYY	low
MHC_B_40_02	ELFARSSDPR	low
MHC_B_40_02	FYYNAFHWAI	low
MHC_B_40_02	SYMMDDLELI	low
MHC_B_40_02	EEGNLLDSYF	low
MHC_B_40_02	LEYFQFVKKLL	low
MHC_B_40_02	GSSDFQVHFLK	low
MHC_B_40_02	FLLPILSQIYT	low
MHC_B_40_02	LPSCPTNFCIF	low

MHC_B_40_02	MIKYCLLKILK	low
MHC_B_40_02	MVRVLTVIKEY	low
MHC_B_40_02	RMILPMSRAFR	low
MHC_B_40_02	SLLERGQQLGV	low
MHC_B_40_02	TFVPIAWAAAY	low
MHC_B_40_02	VALFSSCPVAY	low
MHC_B_40_02	YFYYNAFHWAI	low
MHC_B_40_02	YSLEYFQFVKK	low
MHC_B_42_01	YTAVVPLVY	low
MHC_B_42_01	FRYNGLIHR	low
MHC_B_42_01	ETFGFEIQSY	low
MHC_B_42_01	PLALEGSLQK	low
MHC_B_42_01	GPGAGSLQPLAL	low
MHC_B_44_02	FYYNAFHW	low
MHC_B_44_02	YTSDYFISY	low
MHC_B_44_02	FRYNGLIHR	low
MHC_B_44_02	YTAVVPLVY	low
MHC_B_44_02	ETFGFEIQSY	low
MHC_B_44_03	FPHTELANL	low
MHC_B_44_03	FQILHDRFF	low
MHC_B_44_03	SSKMFNYFK	low
MHC_B_44_03	TEAEKWPFF	low
MHC_B_44_03	FRYNGLIHR	low
MHC_B_44_03	YTAVVPLVY	low
MHC_B_44_03	ETFGFEIQSY	low
MHC_B_44_03	ELFARSSDPR	low
MHC_B_44_03	IVDCLTEMYY	low
MHC_B_44_03	SYMMDDLELI	low
MHC_B_44_03	FLLPILSQIYT	low
MHC_B_44_03	GSSDFQVHFLK	low
MHC_B_44_03	LEYFQFVKKLL	low
MHC_B_44_03	LPSCPTNFCIF	low
MHC_B_44_03	MIKYCLLKILK	low
MHC_B_44_03	MVRVLTVIKEY	low
MHC_B_44_03	RMILPMSRAFR	low
MHC_B_44_03	SLLERGQQLGV	low
MHC_B_44_03	TFVPIAWAAAY	low
MHC_B_44_03	VALFSSCPVAY	low
MHC_B_44_03	YFYYNAFHWAI	low
MHC_B_44_03	YSLEYFQFVKK	low
MHC_B_45_01	YTAVVPLVY	low
MHC_B_45_01	FRYNGLIHR	low
MHC_B_45_01	ETFGFEIQSY	low

MHC_B_45_01	EEGNLLDSYF	low
MHC_B_45_06	YEHYFVFAA	low
MHC_B_45_06	FEYGGFPPA	low
MHC_B_45_06	FRMLAWHVL	low
MHC_B_45_06	MPMKGRFPI	low
MHC_B_45_06	RRHWGGNVL	low
MHC_B_45_06	SILPISWAY	low
MHC_B_45_06	SVDSDHLGY	low
MHC_B_45_06	TFMDHVLRY	low
MHC_B_45_06	YYWPRPRRY	low
MHC_B_45_06	YYRYPTGESY	low
MHC_B_46_01	FIKDGSSTY	low
MHC_B_46_01	SIRDGVRAY	low
MHC_B_46_01	KAGQVVTIW	low
MHC_B_46_01	AMFIGHATA	low
MHC_B_46_01	FAAPHRGVA	low
MHC_B_46_01	FLFDRLTNG	low
MHC_B_46_01	FQWHEAMFL	low
MHC_B_46_01	FTFWTFANY	low
MHC_B_46_01	FWAWSVLRV	low
MHC_B_46_01	GLEAYIQGI	low
MHC_B_46_01	GTEYRLTLY	low
MHC_B_46_01	GVRQFSGWM	low
MHC_B_46_01	IIYVGCGER	low
MHC_B_46_01	IYDFYYLDY	low
MHC_B_46_01	KVRDRNFQL	low
MHC_B_46_01	LMQCWQLLA	low
MHC_B_46_01	LQDDFDFNY	low
MHC_B_46_01	LVDENQSWY	low
MHC_B_46_01	MQDVFTFYV	low
MHC_B_46_01	NTDDFPLTL	low
MHC_B_46_01	NTFKFGVIY	low
MHC_B_46_01	RPNRQLGSM	low
MHC_B_46_01	RTDNGGWAH	low
MHC_B_46_01	SAEVVTLWY	low
MHC_B_46_01	SLFNWLWYE	low
MHC_B_46_01	SVDSDHLGY	low
MHC_B_46_01	TLKDGDFIL	low
MHC_B_46_01	VLIRRCHYL	low
MHC_B_46_01	VLWKSYPLV	low
MHC_B_46_01	VVPSYIPLV	low
MHC_B_46_01	WSTIWRQLY	low
MHC_B_46_01	WTDLFDNKV	low

MHC_B_46_01	YITDYSNDI	low
MHC_B_46_01	YSDNEMLTH	low
MHC_B_46_01	YYFSYPLFV	low
MHC_B_46_01	ITSQDVLHSW	low
MHC_B_46_01	ISDSNPFLTQW	low
MHC_B_48_01	RVYNNTARY	low
MHC_B_51_01	FPHTELANL	low
MHC_B_51_01	DPIFLLHHA	low
MHC_B_51_01	VALFSSCPV	low
MHC_B_51_01	YPGFGEHLI	low
MHC_B_51_01	HPQKVTKFM	low
MHC_B_51_01	SPSCPLERF	low
MHC_B_51_01	HPEDTGQVF	low
MHC_B_51_01	GPQFPFTGV	low
MHC_B_51_01	IPLYRNGDF	low
MHC_B_51_01	APGKGLEWV	low
MHC_B_51_01	AMFIGHATA	low
MHC_B_51_01	ETFNTPAMY	low
MHC_B_51_01	FAAPHRGVA	low
MHC_B_51_01	FLFDRLTNG	low
MHC_B_51_01	FQILHDRFF	low
MHC_B_51_01	FQWHEAMFL	low
MHC_B_51_01	GLEAYIQGI	low
MHC_B_51_01	GTEYRLTLY	low
MHC_B_51_01	GVRQFSGWM	low
MHC_B_51_01	IIYVGCGER	low
MHC_B_51_01	IYDFYYLDY	low
MHC_B_51_01	KVRDRNFQL	low
MHC_B_51_01	LMQCWQLLA	low
MHC_B_51_01	LQDDFDFNY	low
MHC_B_51_01	LVDENQSWY	low
MHC_B_51_01	MQDVFTFYV	low
MHC_B_51_01	NTDDFPLTL	low
MHC_B_51_01	NTFKFGVIY	low
MHC_B_51_01	RPNRQLGSM	low
MHC_B_51_01	RTDNGGWAH	low
MHC_B_51_01	SAEVVTLWY	low
MHC_B_51_01	SLFNWLWYE	low
MHC_B_51_01	SSKMFNYFK	low
MHC_B_51_01	SVDSDHLGY	low
MHC_B_51_01	TEAEKWPFF	low
MHC_B_51_01	TLKDGDFIL	low
MHC_B_51_01	VLIRRCHYL	low

MHC_B_51_01	VLWKSYPLV	low
MHC_B_51_01	WSTIWRQLY	low
MHC_B_51_01	WTDLFDNKV	low
MHC_B_51_01	YITDYSNDI	low
MHC_B_51_01	YSDNEMLTH	low
MHC_B_51_01	YTFCGTIEY	low
MHC_B_51_01	YTSDYFISY	low
MHC_B_51_01	YTYPCIPEY	low
MHC_B_51_01	FRYNGLIHR	low
MHC_B_51_01	YTAVVPLVY	low
MHC_B_51_01	LPGPSDTPIL	low
MHC_B_51_01	ELFARSSDPR	low
MHC_B_51_01	IVDCLTEMYY	low
MHC_B_51_01	SYMMDDLELI	low
MHC_B_51_01	ETFGFEIQSY	low
MHC_B_51_01	PLALEGSLQK	low
MHC_B_51_01	FLLPILSQIYT	low
MHC_B_51_01	GSSDFQVHFLK	low
MHC_B_51_01	LEYFQFVKKLL	low
MHC_B_51_01	LPSCPTNFCIF	low
MHC_B_51_01	MIKYCLLKILK	low
MHC_B_51_01	MVRVLTVIKEY	low
MHC_B_51_01	RMILPMSRAFR	low
MHC_B_51_01	SLLERGQQLGV	low
MHC_B_51_01	TFVPIAWAAAY	low
MHC_B_51_01	VALFSSCPVAY	low
MHC_B_51_01	YFYYNAFHWAI	low
MHC_B_51_01	YSLEYFQFVKK	low
MHC_B_51_01	QPGGSLRLSCA	low
MHC_B_51_01	GPGAGSLQPLAL	low
MHC_B_52_01	RGPDAFRF	low
MHC_B_52_01	RQFPTAFEF	low
MHC_B_52_01	SGKLKVPEW	low
MHC_B_52_01	RGVEKPPHL	low
MHC_B_52_01	AGVWSQDKW	low
MHC_B_52_01	SGGPKYEYRW	low
MHC_B_52_01	KGFTDADNTW	low
MHC_B_52_01	KGFSEEHNTW	low
MHC_B_53_01	CPLERFAEL	low
MHC_B_53_01	FPHTELANL	low
MHC_B_53_01	HPEDTGQVF	low
MHC_B_53_01	TPMFNDINI	low
MHC_B_53_01	LPSIPVHPI	low

MHC_B_53_01	VPIAWAAAY	low
MHC_B_53_01	LPFRNCPRF	low
MHC_B_53_01	SPSCPLERF	low
MHC_B_53_01	APGKGLEWV	low
MHC_B_53_01	HPQKVTKFM	low
MHC_B_53_01	YTAVVPLVY	low
MHC_B_53_01	LPKEACMEI	low
MHC_B_53_01	FQILHDRFF	low
MHC_B_53_01	SSKMFNYFK	low
MHC_B_53_01	TEAEKWPFF	low
MHC_B_53_01	DPIFLLHHA	low
MHC_B_53_01	GPQFPFTGV	low
MHC_B_53_01	IPLYRNGDF	low
MHC_B_53_01	FRYNGLIHR	low
MHC_B_53_01	IVDCLTEMYY	low
MHC_B_53_01	LPGPSDTPIL	low
MHC_B_53_01	ELFARSSDPR	low
MHC_B_53_01	SYMMDDLELI	low
MHC_B_53_01	ETFGFEIQSY	low
MHC_B_53_01	PLALEGSLQK	low
MHC_B_53_01	LPSCPTNFCIF	low
MHC_B_53_01	QPGGSLRLSCA	low
MHC_B_53_01	FLLPILSQIYT	low
MHC_B_53_01	GSSDFQVHFLK	low
MHC_B_53_01	LEYFQFVKKLL	low
MHC_B_53_01	MIKYCLLKILK	low
MHC_B_53_01	MVRVLTVIKEY	low
MHC_B_53_01	RMILPMSRAFR	low
MHC_B_53_01	SLLERGQQLGV	low
MHC_B_53_01	TFVPIAWAAAY	low
MHC_B_53_01	VALFSSCPVAY	low
MHC_B_53_01	YFYYNAFHWAI	low
MHC_B_53_01	YSLEYFQFVKK	low
MHC_B_53_01	GPGAGSLQPLAL	low
MHC_B_54_01	LPKEACMEI	low
MHC_B_54_01	LPFRNCPRF	low
MHC_B_54_01	MPREDAHFI	low
MHC_B_54_01	CPLERFAEL	low
MHC_B_54_01	FPPEGVSIW	low
MHC_B_54_01	TPMFNDINI	low
MHC_B_54_01	GPQFPFTGV	low
MHC_B_54_01	HPEDTGQVF	low
MHC_B_54_01	HPQKVTKFM	low

MHC_B_54_01	IPLYRNGDF	low
MHC_B_54_01	SPSCPLERF	low
MHC_B_54_01	YTAVVPLVY	low
MHC_B_54_01	APGKGLEWV	low
MHC_B_54_01	FRYNGLIHR	low
MHC_B_54_01	ETFGFEIQSY	low
MHC_B_54_01	LPGPSDTPIL	low
MHC_B_54_01	PLALEGSLQK	low
MHC_B_54_01	QPGGSLRLSCA	low
MHC_B_54_01	GPGAGSLQPLAL	low
MHC_B_57_01	FAFVTDNTY	low
MHC_B_57_01	YTAVVPLVY	low
MHC_B_57_01	RTFHHGVRV	low
MHC_B_57_01	RVYNNTARY	low
MHC_B_57_01	TVAPFNPTV	low
MHC_B_57_01	KSNGTIIHV	low
MHC_B_57_01	AMFIGHATA	low
MHC_B_57_01	FAAPHRGVA	low
MHC_B_57_01	FLFDRLTNG	low
MHC_B_57_01	FQWHEAMFL	low
MHC_B_57_01	FWAWSVLRV	low
MHC_B_57_01	GLEAYIQGI	low
MHC_B_57_01	GTEYRLTLY	low
MHC_B_57_01	GVRQFSGWM	low
MHC_B_57_01	IIYVGCGER	low
MHC_B_57_01	IYDFYYLDY	low
MHC_B_57_01	LMQCWQLLA	low
MHC_B_57_01	LQDDFDFNY	low
MHC_B_57_01	LVDENQSWY	low
MHC_B_57_01	MQDVFTFYV	low
MHC_B_57_01	NTDDFPLTL	low
MHC_B_57_01	RPNRQLGSM	low
MHC_B_57_01	RTDNGGWAH	low
MHC_B_57_01	SAEVVTLWY	low
MHC_B_57_01	SLFNWLWYE	low
MHC_B_57_01	SVDSDHLGY	low
MHC_B_57_01	TLKDGDFIL	low
MHC_B_57_01	VLIRRCHYL	low
MHC_B_57_01	VLWKSYPLV	low
MHC_B_57_01	VVPSYIPLV	low
MHC_B_57_01	WTDLFDNKV	low
MHC_B_57_01	YITDYSNDI	low
MHC_B_57_01	YSDNEMLTH	low

MHC_B_57_01	YYFSYPLFV	low
MHC_B_57_01	SIRDGVRAY	low
MHC_B_57_01	FIKDGSSTY	low
MHC_B_57_01	FRYNGLIHR	low
MHC_B_57_01	ETFGFEIQSY	low
MHC_B_57_01	YMIDPSGVSY	low
MHC_B_58_01	YPAEITLTW	low
MHC_B_58_01	FAFVTDNTY	low
MHC_B_58_01	YTYPCIPEY	low
MHC_B_58_01	VGYVDDTQF	low
MHC_B_58_01	KSDLQPPNY	low
MHC_B_58_01	ILGPPGSVY	low
MHC_B_58_01	MCFHQHLMY	low
MHC_B_58_01	YTSDYFISY	low
MHC_B_58_01	DPSGAYFAW	low
MHC_B_58_01	IADMGHLKY	low
MHC_B_58_01	STDHIPILY	low
MHC_B_58_01	ATDFKFAMY	low
MHC_B_58_01	CHKGWGVSV	low
MHC_B_58_01	CSDDGFWSK	low
MHC_B_58_01	CSEYVKDIY	low
MHC_B_58_01	DFDGTPRLY	low
MHC_B_58_01	DSDGSFFLY	low
MHC_B_58_01	EADPTGHSY	low
MHC_B_58_01	EPEPHILLF	low
MHC_B_58_01	ETFNTPAMY	low
MHC_B_58_01	EVDPIGHLY	low
MHC_B_58_01	FHMDPSGTF	low
MHC_B_58_01	FPEHIFPAL	low
MHC_B_58_01	FPHTELANL	low
MHC_B_58_01	FQILHDRFF	low
MHC_B_58_01	GHLENNPAL	low
MHC_B_58_01	GLENGLNYI	low
MHC_B_58_01	GRNSFEVRV	low
MHC_B_58_01	GRPNCFQIV	low
MHC_B_58_01	GSEDRDLLY	low
MHC_B_58_01	IPYHIVNIV	low
MHC_B_58_01	IVDCLTEMY	low
MHC_B_58_01	KCDICTDEY	low
MHC_B_58_01	LPRWPPPQL	low
MHC_B_58_01	LVYNHCEHG	low
MHC_B_58_01	MIEPRTLQY	low
MHC_B_58_01	NPNSPSITY	low

MHC_B_58_01	NQRETTVVW	low
MHC_B_58_01	NRRFVNVVP	low
MHC_B_58_01	RPRHQGVMV	low
MHC_B_58_01	RRFFPYYVY	low
MHC_B_58_01	RRHWGGNVL	low
MHC_B_58_01	RYFSVTRPL	low
MHC_B_58_01	SPRYIFTML	low
MHC_B_58_01	SQFGGGSQY	low
MHC_B_58_01	SRDKTIIMW	low
MHC_B_58_01	SSKMFNYFK	low
MHC_B_58_01	TEAEKWPFF	low
MHC_B_58_01	WVGRASDPD	low
MHC_B_58_01	YFDPANGKF	low
MHC_B_58_01	YGDTEAICR	low
MHC_B_58_01	YHEDIHTYL	low
MHC_B_58_01	YLDDPDLKY	low
MHC_B_58_01	YLDFGGPEG	low
MHC_B_58_01	YLGSWATGK	low
MHC_B_58_01	YVDHYYRDY	low
MHC_B_58_01	FIKDGSSTY	low
MHC_B_58_01	SIRDGVRAY	low
MHC_B_58_01	FRYNGLIHR	low
MHC_B_58_01	YMIDPSGVSY	low
MHC_B_58_01	ETFGFEIQSY	low
MHC_B_58_01	ELFARSSDPR	low
MHC_B_58_01	IVDCLTEMYY	low
MHC_B_58_01	SYMMDDLELI	low
MHC_B_58_01	FLLPILSQIYT	low
MHC_B_58_01	GSSDFQVHFLK	low
MHC_B_58_01	LEYFQFVKKLL	low
MHC_B_58_01	LPSCPTNFCIF	low
MHC_B_58_01	MIKYCLLKILK	low
MHC_B_58_01	MVRVLTVIKEY	low
MHC_B_58_01	RMILPMSRAFR	low
MHC_B_58_01	SLLERGQQLGV	low
MHC_B_58_01	TFVPIAWAAAY	low
MHC_B_58_01	VALFSSCPVAY	low
MHC_B_58_01	YFYYNAFHWAI	low
MHC_B_58_01	YSLEYFQFVKK	low
MHC_B_58_02	KAGQVVTIW	low
MHC_B_58_02	YTAVVPLVY	low
MHC_B_58_02	ITSQDVLHSW	low
MHC_B_58_02	ISDSNPFLTQW	low

MHC_B_73_01	FRMLAWHVL	low
MHC_B_73_01	MPMKGRFPI	low
MHC_B_73_01	FPLWWVSSI	low
MHC_B_83_01	MPMKGRFPI	low
MHC_B_83_01	FEYGGFPPA	low
MHC_B_83_01	RRHWGGNVL	low
MHC_B_83_01	SVDSDHLGY	low
MHC_B_83_01	TFMDHVLRY	low
MHC_B_83_01	YEHYFVFAA	low
MHC_B_83_01	YYWPRPRRY	low
MHC_B_83_01	YYRYPTGESY	low
MHC_C_03_03	YMNGTMSQV	low
MHC_C_03_03	SLDDYNHLV	low
MHC_C_04_01	FYYNAFHW	low
MHC_C_04_01	YYNAFHWA	low
MHC_C_04_01	TEAEKWPFF	low
MHC_C_04_01	FEYGGFPPA	low
MHC_C_04_01	FRMLAWHVL	low
MHC_C_04_01	MPMKGRFPI	low
MHC_C_04_01	RRHWGGNVL	low
MHC_C_04_01	SILPISWAY	low
MHC_C_04_01	SVDSDHLGY	low
MHC_C_04_01	TFMDHVLRY	low
MHC_C_04_01	YEHYFVFAA	low
MHC_C_04_01	FPHTELANL	low
MHC_C_04_01	FQILHDRFF	low
MHC_C_04_01	FYYNAFHWA	low
MHC_C_04_01	KYFIPVIEI	low
MHC_C_04_01	NTDDFPLTL	low
MHC_C_04_01	SSKMFNYFK	low
MHC_C_04_01	YFYYNAFHW	low
MHC_C_04_01	YSLEYFQFV	low
MHC_C_04_01	YYNAFHWAI	low
MHC_C_04_01	YYWPRPRRY	low
MHC_C_04_01	YYRYPTGESY	low
MHC_C_04_01	ELFARSSDPR	low
MHC_C_04_01	FYYNAFHWAI	low
MHC_C_04_01	IVDCLTEMYY	low
MHC_C_04_01	SYMMDDLELI	low
MHC_C_04_01	FLLPILSQIYT	low
MHC_C_04_01	GSSDFQVHFLK	low
MHC_C_04_01	LEYFQFVKKLL	low
MHC_C_04_01	LPSCPTNFCIF	low

MHC_C_04_01	MIKYCLLKILK	low
MHC_C_04_01	MVRVLTVIKEY	low
MHC_C_04_01	RMILPMSRAFR	low
MHC_C_04_01	SLLERGQQLGV	low
MHC_C_04_01	TFVPIAWAAAY	low
MHC_C_04_01	VALFSSCPVAY	low
MHC_C_04_01	YFYYNAFHWAI	low
MHC_C_04_01	YSLEYFQFVKK	low
MHC_C_05_01	YFDPANGKF	low
MHC_C_05_01	SLDDYNHLV	low
MHC_C_05_01	NTDDFPLTL	low
MHC_C_05_01	MPMKGRFPI	low
MHC_C_06_02	FQILHDRFF	low
MHC_C_06_02	TFMDHVLRY	low
MHC_C_06_02	FRYNGLIHR	low
MHC_C_06_02	YTAVVPLVY	low
MHC_C_06_02	FPHTELANL	low
MHC_C_06_02	MPMKGRFPI	low
MHC_C_06_02	SSKMFNYFK	low
MHC_C_06_02	TEAEKWPFF	low
MHC_C_06_02	ELFARSSDPR	low
MHC_C_06_02	IVDCLTEMYY	low
MHC_C_06_02	SYMMDDLELI	low
MHC_C_06_02	ETFGFEIQSY	low
MHC_C_06_02	FLLPILSQIYT	low
MHC_C_06_02	GSSDFQVHFLK	low
MHC_C_06_02	LEYFQFVKKLL	low
MHC_C_06_02	LPSCPTNFCIF	low
MHC_C_06_02	MIKYCLLKILK	low
MHC_C_06_02	MVRVLTVIKEY	low
MHC_C_06_02	RMILPMSRAFR	low
MHC_C_06_02	SLLERGQQLGV	low
MHC_C_06_02	TFVPIAWAAAY	low
MHC_C_06_02	VALFSSCPVAY	low
MHC_C_06_02	YFYYNAFHWAI	low
MHC_C_06_02	YSLEYFQFVKK	low
MHC_C_07_01	TFMDHVLRY	low
MHC_C_07_01	SSKMFNYFK	low
MHC_C_07_01	MPMKGRFPI	low
MHC_C_07_01	FPHTELANL	low
MHC_C_07_01	FQILHDRFF	low
MHC_C_07_01	TEAEKWPFF	low
MHC_C_07_01	ELFARSSDPR	low

MHC_C_07_01	IVDCLTEMYY	low
MHC_C_07_01	SYMMDDLELI	low
MHC_C_07_01	VALFSSCPVAY	low
MHC_C_07_01	MVRVLTVIKEY	low
MHC_C_07_01	TFVPIAWAAAY	low
MHC_C_07_01	YFYYNAFHWAI	low
MHC_C_07_01	FLLPILSQIYT	low
MHC_C_07_01	GSSDFQVHFLK	low
MHC_C_07_01	LEYFQFVKKLL	low
MHC_C_07_01	LPSCPTNFCIF	low
MHC_C_07_01	MIKYCLLKILK	low
MHC_C_07_01	SLLERGQQLGV	low
MHC_C_07_01	YSLEYFQFVKK	low
MHC_C_07_02	YYNAFHWA	low
MHC_C_07_02	FYYNAFHW	low
MHC_C_07_02	RRFFPYYVY	low
MHC_C_07_02	YYSNKAYQY	low
MHC_C_07_02	MLYPRVWPY	low
MHC_C_07_02	YYNAFHWAI	low
MHC_C_07_02	FYYNAFHWA	low
MHC_C_07_02	YFYYNAFHW	low
MHC_C_07_02	FPLWWVSSI	low
MHC_C_07_02	MPMKGRFPI	low
MHC_C_07_02	FYYNAFHWAI	low
MHC_C_08_02	WTDLFDNKV	low
MHC_C_08_02	LVDENQSWY	low
MHC_C_08_02	YLDDPDLKY	low
MHC_C_08_02	YSLEYFQFV	low
MHC_C_08_02	YTAVVPLVY	low
MHC_C_08_02	YTFCGTIEY	low
MHC_C_12_03	YMNGTMSQV	low
MHC_C_12_03	YTAVVPLVY	low
MHC_C_12_03	YYWPRPRRY	low
MHC_C_12_03	EVAPHPVYY	low
MHC_C_14_02	MLYPRVWPY	low
MHC_C_14_02	FPHTELANL	low
MHC_C_14_02	FQILHDRFF	low
MHC_C_14_02	SSKMFNYFK	low
MHC_C_14_02	TEAEKWPFF	low
MHC_C_14_02	SYMMDDLELI	low
MHC_C_14_02	ELFARSSDPR	low
MHC_C_14_02	IVDCLTEMYY	low
MHC_C_14_02	TFVPIAWAAAY	low

MHC_C_14_02	VALFSSCPVAY	low
MHC_C_14_02	YFYYNAFHWAI	low
MHC_C_14_02	LEYFQFVKKLL	low
MHC_C_14_02	MVRVLTVIKEY	low
MHC_C_14_02	RMILPMSRAFR	low
MHC_C_14_02	FLLPILSQIYT	low
MHC_C_14_02	GSSDFQVHFLK	low
MHC_C_14_02	LPSCPTNFCIF	low
MHC_C_14_02	MIKYCLLKILK	low
MHC_C_14_02	SLLERGQQLGV	low
MHC_C_14_02	YSLEYFQFVKK	low
MHC_C_15_02	YMNGTMSQV	low
MHC_C_15_02	NAFHHPSAV	low
MHC_C_15_02	YYFSYPLFV	low
MHC_C_15_02	MLYPRVWPY	low

Supplementary Material S2 The 340 ranked features in MaxRel and mRMR feature lists.

(1) MaxRel feature list

Order	Feature name	Feature type	Score	Feature component
1	AAC_7	AAC	0.027	Protein feature
2	AAC_13	AAC	0.021	Protein feature
3	CD_21	CD	0.021	Protein feature
4	Polarity_13	polarity	0.02	Protein feature
5	AAC_12	AAC	0.02	Protein feature
6	Polarity_6	polarity	0.019	Protein feature
7	CD_24	CD	0.019	Protein feature
8	CD_36	CD	0.019	Protein feature
9	CD_30	CD	0.018	Protein feature
10	AAC_5	AAC	0.018	Protein feature
11	EC_40	EC	0.017	Protein feature
12	MV_6	MV	0.017	Protein feature
13	EC_47	EC	0.017	Protein feature
14	CD_2	CD	0.017	Protein feature
15	AAC_9	AAC	0.015	Protein feature
16	CD_37	CD	0.015	Protein feature
17	Polarity_21	polarity	0.015	Protein feature
18	CD_34	CD	0.015	Protein feature
19	2nd_stru_38	2nd_stru	0.014	Protein feature
20	Polarity_2	polarity	0.014	Protein feature
21	2nd_stru_20	2nd_stru	0.014	Protein feature
22	2nd_stru_30	2nd_stru	0.013	Protein feature
23	AAC_8	AAC	0.013	Protein feature
24	CD_3	CD	0.013	Protein feature
25	EC_37	EC	0.013	Protein feature
26	EC_6	EC	0.013	Protein feature
27	2nd_stru_17	2nd_stru	0.013	Protein feature
28	MV_21	MV	0.013	Protein feature
29	2nd_stru_15	2nd_stru	0.013	Protein feature
30	EC_1	EC	0.013	Protein feature
31	MV_36	MV	0.012	Protein feature
32	Polarity_20	polarity	0.012	Protein feature
33	AAC_19	AAC	0.012	Protein feature
34	MV_47	MV	0.012	Protein feature
35	Polarity_25	polarity	0.012	Protein feature
36	EC_21	EC	0.012	Protein feature
37	EC_9	EC	0.012	Protein feature
38	Polarity_18	polarity	0.011	Protein feature

39	MV_25	MV	0.011	Protein feature
40	CD_44	CD	0.01	Protein feature
41	2nd_stru_22	2nd_stru	0.01	Protein feature
42	2nd_stru_25	2nd_stru	0.01	Protein feature
43	AAC_15	AAC	0.01	Protein feature
44	2nd_stru_28	2nd_stru	0.01	Protein feature
45	2nd_stru_33	2nd_stru	0.01	Protein feature
46	2nd_stru_6	2nd_stru	0.01	Protein feature
47	EC_36	EC	0.01	Protein feature
48	Polarity_16	polarity	0.01	Protein feature
49	EC_44	EC	0.009	Protein feature
50	Polarity_29	polarity	0.009	Protein feature
51	EC_43	EC	0.009	Protein feature
52	CD_8	CD	0.009	Protein feature
53	MV_37	MV	0.009	Protein feature
54	CD_26	CD	0.009	Protein feature
55	EC_3	EC	0.009	Protein feature
56	2nd_stru_23	2nd_stru	0.009	Protein feature
57	2nd_stru_4	2nd_stru	0.008	Protein feature
58	CD_40	CD	0.008	Protein feature
59	AAC_11	AAC	0.008	Protein feature
60	2nd_stru_14	2nd_stru	0.008	Protein feature
61	EC_20	EC	0.008	Protein feature
62	MV_38	MV	0.008	Protein feature
63	MV_20	MV	0.008	Protein feature
64	CD_28	CD	0.008	Protein feature
65	2nd_stru_37	2nd_stru	0.008	Protein feature
66	MV_32	MV	0.008	Protein feature
67	MV_42	MV	0.008	Protein feature
68	EC_14	EC	0.008	Protein feature
69	Polarity_22	polarity	0.007	Protein feature
70	Polarity_49	polarity	0.007	Protein feature
71	EC_49	EC	0.007	Protein feature
72	EC_32	EC	0.007	Protein feature
73	2nd_stru_31	2nd_stru	0.007	Protein feature
74	CD_9	CD	0.007	Protein feature
75	CD_6	CD	0.007	Protein feature
76	2nd_stru_48	2nd_stru	0.007	Protein feature
77	CD_49	CD	0.007	Protein feature
78	CD_47	CD	0.007	Protein feature
79	Polarity_27	polarity	0.007	Protein feature
80	2nd_stru_35	2nd_stru	0.007	Protein feature
81	Polarity_5	polarity	0.007	Protein feature

82	Polarity_24	polarity	0.007	Protein feature
83	EC_26	EC	0.006	Protein feature
84	MV_15	MV	0.006	Protein feature
85	Polarity_42	polarity	0.006	Protein feature
86	MV_40	MV	0.006	Protein feature
87	2nd_stru_41	2nd_stru	0.006	Protein feature
88	EC_34	EC	0.006	Protein feature
89	MV_10	MV	0.006	Protein feature
90	Polarity_46	polarity	0.006	Protein feature
91	Polarity_50	polarity	0.006	Protein feature
92	Polarity_36	polarity	0.006	Protein feature
93	CD_4	CD	0.006	Protein feature
94	peptide_AAC_3	AAC	0.006	Peptide feature
95	AAC_6	AAC	0.006	Protein feature
96	AAC_14	AAC	0.006	Protein feature
97	Polarity_38	polarity	0.006	Protein feature
98	EC_46	EC	0.006	Protein feature
99	MV_48	MV	0.006	Protein feature
100	EC_27	EC	0.006	Protein feature
101	CD_39	CD	0.006	Protein feature
102	EC_30	EC	0.006	Protein feature
103	EC_50	EC	0.005	Protein feature
104	2nd_stru_27	2nd_stru	0.005	Protein feature
105	MV_23	MV	0.005	Protein feature
106	Polarity_43	polarity	0.005	Protein feature
107	EC_38	EC	0.005	Protein feature
108	CD_27	CD	0.005	Protein feature
109	EC_18	EC	0.005	Protein feature
110	2nd_stru_13	2nd_stru	0.005	Protein feature
111	2nd_stru_10	2nd_stru	0.005	Protein feature
112	2nd_stru_12	2nd_stru	0.005	Protein feature
113	EC_16	EC	0.005	Protein feature
114	Polarity_35	polarity	0.005	Protein feature
115	MV_14	MV	0.005	Protein feature
116	2nd_stru_9	2nd_stru	0.005	Protein feature
117	MV_30	MV	0.005	Protein feature
118	2nd_stru_21	2nd_stru	0.005	Protein feature
119	Polarity_1	polarity	0.005	Protein feature
120	MV_24	MV	0.005	Protein feature
121	2nd_stru_36	2nd_stru	0.004	Protein feature
122	EC_48	EC	0.004	Protein feature
123	MV_49	MV	0.004	Protein feature
124	MV_43	MV	0.004	Protein feature

125	EC_25	EC	0.004	Protein feature
126	CD_25	CD	0.004	Protein feature
127	Polarity_10	polarity	0.004	Protein feature
128	Polarity_12	polarity	0.004	Protein feature
129	MV_18	MV	0.004	Protein feature
130	EC_45	EC	0.004	Protein feature
131	peptide_CD_5	CD	0.004	Peptide feature
132	peptide_2nd_stru_10	2nd_stru	0.004	Peptide feature
133	MV_41	MV	0.004	Protein feature
134	CD_23	CD	0.004	Protein feature
135	2nd_stru_39	2nd_stru	0.004	Protein feature
136	Polarity_32	polarity	0.004	Protein feature
137	Polarity_40	polarity	0.004	Protein feature
138	MV_5	MV	0.004	Protein feature
139	2nd_stru_5	2nd_stru	0.004	Protein feature
140	CD_15	CD	0.004	Protein feature
141	Polarity_23	polarity	0.004	Protein feature
142	CD_50	CD	0.004	Protein feature
143	CD_32	CD	0.004	Protein feature
144	CD_14	CD	0.004	Protein feature
145	EC_29	EC	0.004	Protein feature
146	2nd_stru_26	2nd_stru	0.004	Protein feature
147	Polarity_4	polarity	0.004	Protein feature
148	MV_19	MV	0.004	Protein feature
149	EC_17	EC	0.004	Protein feature
150	2nd_stru_49	2nd_stru	0.004	Protein feature
151	EC_10	EC	0.004	Protein feature
152	Polarity_7	polarity	0.004	Protein feature
153	Polarity_26	polarity	0.004	Protein feature
154	2nd_stru_2	2nd_stru	0.004	Protein feature
155	Polarity_31	polarity	0.004	Protein feature
156	CD_38	CD	0.004	Protein feature
157	2nd_stru_11	2nd_stru	0.004	Protein feature
158	peptide_EC_10	EC	0.004	Peptide feature
159	peptide_CD_10	CD	0.003	Peptide feature
160	2nd_stru_43	2nd_stru	0.003	Protein feature
161	CD_48	CD	0.003	Protein feature
162	2nd_stru_32	2nd_stru	0.003	Protein feature
163	EC_28	EC	0.003	Protein feature
164	peptide_Polarity_10	polarity	0.003	Peptide feature
165	CD_45	CD	0.003	Protein feature
166	Polarity_37	polarity	0.003	Protein feature
167	EC_23	EC	0.003	Protein feature

168	2nd_stru_16	2nd_stru	0.003	Protein feature
169	MV_2	MV	0.003	Protein feature
170	AAC_16	AAC	0.003	Protein feature
171	EC_7	EC	0.003	Protein feature
172	2nd_stru_40	2nd_stru	0.003	Protein feature
173	2nd_stru_44	2nd_stru	0.003	Protein feature
174	Polarity_44	polarity	0.003	Protein feature
175	Polarity_8	polarity	0.003	Protein feature
176	MV_27	MV	0.003	Protein feature
177	EC_15	EC	0.003	Protein feature
178	MV_11	MV	0.003	Protein feature
179	MV_9	MV	0.003	Protein feature
180	2nd_stru_45	2nd_stru	0.003	Protein feature
181	2nd_stru_34	2nd_stru	0.003	Protein feature
182	MV_46	MV	0.003	Protein feature
183	MV_35	MV	0.003	Protein feature
184	2nd_stru_46	2nd_stru	0.003	Protein feature
185	2nd_stru_1	2nd_stru	0.003	Protein feature
186	Polarity_45	polarity	0.003	Protein feature
187	peptide_Polarity_1	polarity	0.003	Peptide feature
188	CD_35	CD	0.003	Protein feature
189	Polarity_47	polarity	0.003	Protein feature
190	EC_19	EC	0.003	Protein feature
191	2nd_stru_24	2nd_stru	0.003	Protein feature
192	CD_18	CD	0.003	Protein feature
193	peptide_2nd_stru_7	2nd_stru	0.003	Peptide feature
194	CD_7	CD	0.003	Protein feature
195	CD_22	CD	0.003	Protein feature
196	CD_1	CD	0.003	Protein feature
197	2nd_stru_8	2nd_stru	0.003	Protein feature
198	peptide_2nd_stru_2	2nd_stru	0.003	Peptide feature
199	MV_44	MV	0.002	Protein feature
200	EC_42	EC	0.002	Protein feature
201	2nd_stru_18	2nd_stru	0.002	Protein feature
202	AAC_4	AAC	0.002	Protein feature
203	CD_13	CD	0.002	Protein feature
204	CD_16	CD	0.002	Protein feature
205	EC_12	EC	0.002	Protein feature
206	Polarity_3	polarity	0.002	Protein feature
207	MV_22	MV	0.002	Protein feature
208	2nd_stru_19	2nd_stru	0.002	Protein feature
209	MV_28	MV	0.002	Protein feature
210	Polarity_28	polarity	0.002	Protein feature

211	Polarity_41	polarity	0.002	Protein feature
212	peptide_CD_8	CD	0.002	Peptide feature
213	peptide_MV_10	MV	0.002	Peptide feature
214	CD_17	CD	0.002	Protein feature
215	MV_3	MV	0.002	Protein feature
216	AAC_1	AAC	0.002	Protein feature
217	MV_7	MV	0.002	Protein feature
218	Polarity_19	polarity	0.002	Protein feature
219	EC_39	EC	0.002	Protein feature
220	Polarity_15	polarity	0.002	Protein feature
221	CD_33	CD	0.002	Protein feature
222	peptide_AAC_11	AAC	0.002	Peptide feature
223	MV_33	MV	0.002	Protein feature
224	Polarity_11	polarity	0.002	Protein feature
225	2nd_stru_42	2nd_stru	0.002	Protein feature
226	EC_11	EC	0.002	Protein feature
227	2nd_stru_50	2nd_stru	0.002	Protein feature
228	MV_26	MV	0.002	Protein feature
229	MV_13	MV	0.002	Protein feature
230	EC_41	EC	0.002	Protein feature
231	MV_1	MV	0.002	Protein feature
232	EC_2	EC	0.002	Protein feature
233	peptide_2nd_stru_1	2nd_stru	0.002	Peptide feature
234	MV_45	MV	0.002	Protein feature
235	Polarity_33	polarity	0.002	Protein feature
236	AAC_3	AAC	0.002	Protein feature
237	EC_13	EC	0.002	Protein feature
238	CD_31	CD	0.002	Protein feature
239	2nd_stru_3	2nd_stru	0.002	Protein feature
240	peptide_AAC_4	AAC	0.002	Peptide feature
241	EC_4	EC	0.002	Protein feature
242	peptide_2nd_stru_6	2nd_stru	0.002	Peptide feature
243	Polarity_39	polarity	0.002	Protein feature
244	AAC_20	AAC	0.002	Protein feature
245	EC_31	EC	0.002	Protein feature
246	MV_8	MV	0.002	Protein feature
247	MV_39	MV	0.002	Protein feature
248	peptide_CD_7	CD	0.002	Peptide feature
249	CD_11	CD	0.001	Protein feature
250	peptide_EC_3	EC	0.001	Peptide feature
251	peptide_CD_2	CD	0.001	Peptide feature
252	CD_20	CD	0.001	Protein feature
253	peptide_MV_3	MV	0.001	Peptide feature

254	CD_46	CD	0.001	Protein feature
255	peptide_EC_1	EC	0.001	Peptide feature
256	Polarity_14	polarity	0.001	Protein feature
257	Polarity_48	polarity	0.001	Protein feature
258	MV_34	MV	0.001	Protein feature
259	CD_42	CD	0.001	Protein feature
260	CD_19	CD	0.001	Protein feature
261	AAC_17	AAC	0.001	Protein feature
262	peptide_AAC_19	AAC	0.001	Peptide feature
263	peptide_Polarity_9	polarity	0.001	Peptide feature
264	AAC_2	AAC	0.001	Protein feature
265	2nd_stru_29	2nd_stru	0.001	Protein feature
266	CD_12	CD	0.001	Protein feature
267	Polarity_17	polarity	0.001	Protein feature
268	Polarity_9	polarity	0.001	Protein feature
269	AAC_20	AAC	0.001	Protein feature
270	peptide_Polarity_2	polarity	0.001	Peptide feature
271	peptide_MV_4	MV	0.001	Peptide feature
272	peptide_EC_5	EC	0.001	Peptide feature
273	EC_33	EC	0.001	Protein feature
274	AAC_10	AAC	0.001	Protein feature
275	CD_29	CD	0.001	Protein feature
276	2nd_stru_7	2nd_stru	0.001	Protein feature
277	peptide_EC_9	EC	0.001	Peptide feature
278	peptide_Polarity_7	polarity	0.001	Peptide feature
279	EC_22	EC	0.001	Protein feature
280	peptide_2nd_stru_5	2nd_stru	0.001	Peptide feature
281	MV_4	MV	0.001	Protein feature
282	MV_16	MV	0.001	Protein feature
283	CD_10	CD	0.001	Protein feature
284	CD_41	CD	0.001	Protein feature
285	CD_43	CD	0.001	Protein feature
286	peptide_CD_3	CD	0.001	Peptide feature
287	EC_24	EC	0.001	Protein feature
288	MV_50	MV	0.001	Protein feature
289	peptide_EC_7	EC	0.001	Peptide feature
290	peptide_MV_7	MV	0.001	Peptide feature
291	peptide_EC_6	EC	0.001	Peptide feature
292	MV_29	MV	0.001	Protein feature
293	peptide_Polarity_4	polarity	0.001	Peptide feature
294	peptide_MV_9	MV	0.001	Peptide feature
295	peptide_MV_6	MV	0.001	Peptide feature
296	peptide_MV_2	MV	0.001	Peptide feature

297	EC_35	EC	0.001	Protein feature
298	peptide_Polarity_5	polarity	0.001	Peptide feature
299	peptide_Polarity_8	polarity	0.001	Peptide feature
300	2nd_stru_47	2nd_stru	0.001	Protein feature
301	MV_17	MV	0.001	Protein feature
302	peptide_2nd_stru_4	2nd_stru	0	Peptide feature
303	MV_12	MV	0	Protein feature
304	CD_5	CD	0	Protein feature
305	peptide_MV_5	MV	0	Peptide feature
306	peptide_AAC_6	AAC	0	Peptide feature
307	peptide_EC_8	EC	0	Peptide feature
308	peptide_AAC_1	AAC	0	Peptide feature
309	peptide_EC_4	EC	0	Peptide feature
310	peptide_Polarity_6	polarity	0	Peptide feature
311	peptide_AAC_17	AAC	0	Peptide feature
312	peptide_CD_1	CD	0	Peptide feature
313	peptide_2nd_stru_3	2nd_stru	0	Peptide feature
314	EC_8	EC	0	Protein feature
315	Polarity_30	polarity	0	Protein feature
316	peptide_AAC_15	AAC	0	Peptide feature
317	AAC_18	AAC	0	Protein feature
318	peptide_2nd_stru_8	2nd_stru	0	Peptide feature
319	peptide_AAC_5	AAC	0	Peptide feature
320	peptide_AAC_9	AAC	0	Peptide feature
321	peptide_AAC_10	AAC	0	Peptide feature
322	peptide_AAC_8	AAC	0	Peptide feature
323	peptide_MV_1	MV	0	Peptide feature
324	peptide_AAC_18	AAC	0	Peptide feature
325	Polarity_34	polarity	0	Protein feature
326	peptide_MV_8	MV	0	Peptide feature
327	peptide_AAC_12	AAC	0	Peptide feature
328	peptide_CD_9	CD	0	Peptide feature
329	peptide_CD_4	CD	0	Peptide feature
330	peptide_AAC_14	AAC	0	Peptide feature
331	MV_31	MV	0	Protein feature
332	EC_5	EC	0	Protein feature
333	peptide_2nd_stru_9	2nd_stru	0	Peptide feature
334	peptide_AAC_7	AAC	0	Peptide feature
335	peptide_EC_2	EC	0	Peptide feature
336	peptide_CD_6	CD	0	Peptide feature
337	peptide_Polarity_3	polarity	0	Peptide feature
338	peptide_AAC_13	AAC	0	Peptide feature
339	peptide_AAC_16	AAC	0	Peptide feature

(2) mRMR feature list

Order	Feature name	Feature type	Score	Feature component
1	AAC_7	AAC	0.027	Protein feature
2	peptide_AAC_3	AAC	0.003	Peptide feature
3	peptide_AAC_19	AAC	0	Peptide feature
4	peptide_EC_1	EC	0	Peptide feature
5	peptide_AAC_12	AAC	-0.001	Peptide feature
6	peptide_AAC_11	AAC	-0.001	Peptide feature
7	peptide_AAC_8	AAC	-0.001	Peptide feature
8	MV_48	MV	-0.001	Protein feature
9	peptide_AAC_4	AAC	-0.001	Peptide feature
10	peptide_CD_5	CD	-0.002	Peptide feature
11	Polarity_50	polarity	-0.002	Protein feature
12	peptide_AAC_9	AAC	-0.003	Peptide feature
13	peptide_2nd_stru_10	2nd_stru	-0.002	Peptide feature
14	peptide_2nd_stru_6	2nd_stru	-0.003	Peptide feature
15	peptide_AAC_14	AAC	-0.003	Peptide feature
16	peptide_EC_8	EC	-0.004	Peptide feature
17	peptide_AAC_18	AAC	-0.004	Peptide feature
18	peptide_Polarity_8	polarity	-0.004	Peptide feature
19	peptide_CD_9	CD	-0.004	Peptide feature
20	peptide_AAC_16	AAC	-0.004	Peptide feature
21	2nd_stru_33	2nd_stru	-0.003	Protein feature
22	peptide_AAC_7	AAC	-0.004	Peptide feature
23	AAC_20	AAC	-0.004	Protein feature
24	peptide_AAC_13	AAC	-0.004	Peptide feature
25	peptide_Polarity_10	polarity	-0.004	Peptide feature
26	peptide_AAC_17	AAC	-0.004	Peptide feature
27	peptide_MV_2	MV	-0.004	Peptide feature
28	peptide_AAC_2	AAC	-0.004	Peptide feature
29	2nd_stru_10	2nd_stru	-0.004	Protein feature
30	peptide_AAC_6	AAC	-0.004	Peptide feature
31	peptide_2nd_stru_8	2nd_stru	-0.005	Peptide feature
32	Polarity_43	polarity	-0.005	Protein feature
33	peptide_AAC_1	AAC	-0.005	Peptide feature
34	peptide_CD_3	CD	-0.005	Peptide feature
35	peptide_Polarity_7	polarity	-0.005	Peptide feature
36	AAC_5	AAC	-0.005	Protein feature
37	peptide_MV_9	MV	-0.006	Peptide feature
38	peptide_2nd_stru_7	2nd_stru	-0.006	Peptide feature
39	peptide_MV_6	MV	-0.006	Peptide feature
40	peptide_AAC_5	AAC	-0.006	Peptide feature

41	peptide_EC_5	EC	-0.006	Peptide feature
42	CD_50	CD	-0.006	Protein feature
43	peptide_Polarity_4	polarity	-0.007	Peptide feature
44	peptide_AAC_15	AAC	-0.006	Peptide feature
45	2nd_stru_17	2nd_stru	-0.006	Protein feature
46	peptide_CD_2	CD	-0.006	Peptide feature
47	peptide_2nd_stru_2	2nd_stru	-0.007	Peptide feature
48	peptide_Polarity_1	polarity	-0.007	Peptide feature
49	peptide_Polarity_6	polarity	-0.007	Peptide feature
50	2nd_stru_24	2nd_stru	-0.007	Protein feature
51	peptide_MV_10	MV	-0.007	Peptide feature
52	peptide_CD_1	CD	-0.007	Peptide feature
53	peptide_EC_3	EC	-0.008	Peptide feature
54	peptide_CD_10	CD	-0.008	Peptide feature
55	2nd_stru_14	2nd_stru	-0.008	Protein feature
56	peptide_CD_7	CD	-0.008	Peptide feature
57	peptide_MV_4	MV	-0.008	Peptide feature
58	Polarity_42	polarity	-0.008	Protein feature
59	peptide_2nd_stru_9	2nd_stru	-0.008	Peptide feature
60	peptide_Polarity_2	polarity	-0.008	Peptide feature
61	MV_38	MV	-0.008	Protein feature
62	peptide_CD_8	CD	-0.008	Peptide feature
63	peptide_CD_6	CD	-0.009	Peptide feature
64	peptide_2nd_stru_4	2nd_stru	-0.009	Peptide feature
65	2nd_stru_25	2nd_stru	-0.009	Protein feature
66	peptide_Polarity_3	polarity	-0.009	Peptide feature
67	peptide_2nd_stru_5	2nd_stru	-0.009	Peptide feature
68	peptide_2nd_stru_1	2nd_stru	-0.009	Peptide feature
69	peptide_Polarity_9	polarity	-0.009	Peptide feature
70	AAC_20	AAC	-0.009	Protein feature
71	peptide_MV_8	MV	-0.01	Peptide feature
72	peptide_AAC_10	AAC	-0.01	Peptide feature
73	MV_25	MV	-0.01	Protein feature
74	peptide_MV_5	MV	-0.01	Peptide feature
75	peptide_EC_6	EC	-0.01	Peptide feature
76	peptide_Polarity_5	polarity	-0.01	Peptide feature
77	peptide_EC_7	EC	-0.01	Peptide feature
78	CD_21	CD	-0.01	Protein feature
79	peptide_MV_3	MV	-0.011	Peptide feature
80	MV_14	MV	-0.011	Protein feature
81	peptide_CD_4	CD	-0.011	Peptide feature
82	peptide_EC_9	EC	-0.011	Peptide feature
83	CD_35	CD	-0.012	Protein feature

84	peptide_EC_10	EC	-0.012	Peptide feature
85	peptide_EC_4	EC	-0.012	Peptide feature
86	EC_36	EC	-0.012	Protein feature
87	peptide_MV_1	MV	-0.012	Peptide feature
88	peptide_2nd_stru_3	2nd_stru	-0.013	Peptide feature
89	AAC_6	AAC	-0.012	Protein feature
90	peptide_EC_2	EC	-0.013	Peptide feature
91	2nd_stru_30	2nd_stru	-0.013	Protein feature
92	peptide_MV_7	MV	-0.014	Peptide feature
93	EC_41	EC	-0.014	Protein feature
94	2nd_stru_11	2nd_stru	-0.014	Protein feature
95	CD_18	CD	-0.015	Protein feature
96	EC_27	EC	-0.015	Protein feature
97	EC_16	EC	-0.016	Protein feature
98	Polarity_11	polarity	-0.016	Protein feature
99	CD_25	CD	-0.017	Protein feature
100	2nd_stru_4	2nd_stru	-0.017	Protein feature
101	EC_28	EC	-0.018	Protein feature
102	MV_20	MV	-0.019	Protein feature
103	AAC_1	AAC	-0.019	Protein feature
104	2nd_stru_36	2nd_stru	-0.02	Protein feature
105	CD_46	CD	-0.021	Protein feature
106	MV_12	MV	-0.021	Protein feature
107	Polarity_8	polarity	-0.022	Protein feature
108	2nd_stru_16	2nd_stru	-0.022	Protein feature
109	MV_22	MV	-0.023	Protein feature
110	CD_26	CD	-0.023	Protein feature
111	AAC_18	AAC	-0.024	Protein feature
112	MV_43	MV	-0.025	Protein feature
113	Polarity_30	polarity	-0.025	Protein feature
114	MV_28	MV	-0.025	Protein feature
115	2nd_stru_5	2nd_stru	-0.026	Protein feature
116	EC_44	EC	-0.026	Protein feature
117	CD_10	CD	-0.027	Protein feature
118	MV_40	MV	-0.027	Protein feature
119	CD_15	CD	-0.028	Protein feature
120	Polarity_20	polarity	-0.028	Protein feature
121	EC_19	EC	-0.029	Protein feature
122	Polarity_27	polarity	-0.029	Protein feature
123	MV_3	MV	-0.03	Protein feature
124	2nd_stru_44	2nd_stru	-0.03	Protein feature
125	AAC_2	AAC	-0.03	Protein feature
126	EC_11	EC	-0.031	Protein feature

127	EC_38	EC	-0.031	Protein feature
128	MV_32	MV	-0.031	Protein feature
129	CD_17	CD	-0.031	Protein feature
130	CD_1	CD	-0.033	Protein feature
131	Polarity_41	polarity	-0.033	Protein feature
132	2nd_stru_2	2nd_stru	-0.033	Protein feature
133	EC_29	EC	-0.033	Protein feature
134	EC_12	EC	-0.034	Protein feature
135	EC_46	EC	-0.034	Protein feature
136	MV_16	MV	-0.035	Protein feature
137	CD_32	CD	-0.035	Protein feature
138	CD_16	CD	-0.036	Protein feature
139	Polarity_4	polarity	-0.036	Protein feature
140	Polarity_12	polarity	-0.037	Protein feature
141	MV_13	MV	-0.037	Protein feature
142	Polarity_39	polarity	-0.037	Protein feature
143	2nd_stru_46	2nd_stru	-0.038	Protein feature
144	CD_8	CD	-0.038	Protein feature
145	AAC_16	AAC	-0.038	Protein feature
146	2nd_stru_47	2nd_stru	-0.039	Protein feature
147	MV_11	MV	-0.039	Protein feature
148	2nd_stru_13	2nd_stru	-0.04	Protein feature
149	Polarity_28	polarity	-0.04	Protein feature
150	Polarity_34	polarity	-0.041	Protein feature
151	2nd_stru_9	2nd_stru	-0.041	Protein feature
152	Polarity_36	polarity	-0.041	Protein feature
153	Polarity_23	polarity	-0.042	Protein feature
154	MV_33	MV	-0.042	Protein feature
155	2nd_stru_43	2nd_stru	-0.042	Protein feature
156	Polarity_26	polarity	-0.042	Protein feature
157	MV_30	MV	-0.043	Protein feature
158	MV_21	MV	-0.044	Protein feature
159	2nd_stru_1	2nd_stru	-0.044	Protein feature
160	Polarity_48	polarity	-0.044	Protein feature
161	Polarity_3	polarity	-0.044	Protein feature
162	CD_43	CD	-0.045	Protein feature
163	EC_14	EC	-0.046	Protein feature
164	CD_23	CD	-0.046	Protein feature
165	MV_46	MV	-0.046	Protein feature
166	EC_43	EC	-0.046	Protein feature
167	CD_5	CD	-0.047	Protein feature
168	CD_14	CD	-0.047	Protein feature
169	CD_29	CD	-0.047	Protein feature

170	AAC_19	AAC	-0.048	Protein feature
171	2nd_stru_34	2nd_stru	-0.049	Protein feature
172	EC_48	EC	-0.049	Protein feature
173	MV_9	MV	-0.049	Protein feature
174	CD_38	CD	-0.049	Protein feature
175	EC_3	EC	-0.049	Protein feature
176	MV_23	MV	-0.049	Protein feature
177	Polarity_7	polarity	-0.05	Protein feature
178	Polarity_33	polarity	-0.051	Protein feature
179	EC_8	EC	-0.051	Protein feature
180	Polarity_38	polarity	-0.052	Protein feature
181	MV_35	MV	-0.052	Protein feature
182	2nd_stru_41	2nd_stru	-0.053	Protein feature
183	MV_49	MV	-0.053	Protein feature
184	EC_26	EC	-0.052	Protein feature
185	CD_33	CD	-0.053	Protein feature
186	2nd_stru_39	2nd_stru	-0.053	Protein feature
187	MV_31	MV	-0.054	Protein feature
188	Polarity_15	polarity	-0.055	Protein feature
189	2nd_stru_40	2nd_stru	-0.055	Protein feature
190	MV_39	MV	-0.055	Protein feature
191	AAC_17	AAC	-0.055	Protein feature
192	EC_50	EC	-0.056	Protein feature
193	MV_24	MV	-0.056	Protein feature
194	Polarity_19	polarity	-0.057	Protein feature
195	EC_24	EC	-0.057	Protein feature
196	CD_42	CD	-0.057	Protein feature
197	2nd_stru_3	2nd_stru	-0.058	Protein feature
198	2nd_stru_28	2nd_stru	-0.058	Protein feature
199	MV_34	MV	-0.059	Protein feature
200	Polarity_24	polarity	-0.06	Protein feature
201	EC_30	EC	-0.06	Protein feature
202	MV_29	MV	-0.06	Protein feature
203	Polarity_45	polarity	-0.06	Protein feature
204	CD_31	CD	-0.061	Protein feature
205	CD_40	CD	-0.061	Protein feature
206	AAC_3	AAC	-0.061	Protein feature
207	CD_45	CD	-0.062	Protein feature
208	CD_20	CD	-0.062	Protein feature
209	EC_1	EC	-0.063	Protein feature
210	CD_41	CD	-0.063	Protein feature
211	CD_11	CD	-0.063	Protein feature
212	EC_20	EC	-0.064	Protein feature

213	2nd_stru_48	2nd_stru	-0.064	Protein feature
214	MV_27	MV	-0.065	Protein feature
215	EC_39	EC	-0.065	Protein feature
216	CD_7	CD	-0.065	Protein feature
217	2nd_stru_6	2nd_stru	-0.065	Protein feature
218	CD_48	CD	-0.065	Protein feature
219	AAC_14	AAC	-0.066	Protein feature
220	CD_39	CD	-0.066	Protein feature
221	MV_42	MV	-0.067	Protein feature
222	EC_15	EC	-0.067	Protein feature
223	2nd_stru_19	2nd_stru	-0.067	Protein feature
224	2nd_stru_31	2nd_stru	-0.068	Protein feature
225	2nd_stru_26	2nd_stru	-0.068	Protein feature
226	Polarity_14	polarity	-0.069	Protein feature
227	Polarity_40	polarity	-0.069	Protein feature
228	EC_33	EC	-0.069	Protein feature
229	EC_49	EC	-0.07	Protein feature
230	CD_34	CD	-0.071	Protein feature
231	EC_22	EC	-0.071	Protein feature
232	EC_35	EC	-0.072	Protein feature
233	MV_2	MV	-0.072	Protein feature
234	MV_7	MV	-0.072	Protein feature
235	CD_22	CD	-0.072	Protein feature
236	2nd_stru_21	2nd_stru	-0.073	Protein feature
237	MV_26	MV	-0.073	Protein feature
238	Polarity_10	polarity	-0.074	Protein feature
239	MV_1	MV	-0.074	Protein feature
240	EC_45	EC	-0.075	Protein feature
241	MV_18	MV	-0.075	Protein feature
242	MV_8	MV	-0.075	Protein feature
243	EC_42	EC	-0.075	Protein feature
244	EC_17	EC	-0.076	Protein feature
245	MV_41	MV	-0.076	Protein feature
246	CD_49	CD	-0.076	Protein feature
247	EC_31	EC	-0.077	Protein feature
248	EC_4	EC	-0.077	Protein feature
249	Polarity_17	polarity	-0.078	Protein feature
250	CD_9	CD	-0.078	Protein feature
251	Polarity_37	polarity	-0.078	Protein feature
252	MV_45	MV	-0.078	Protein feature
253	2nd_stru_8	2nd_stru	-0.079	Protein feature
254	Polarity_1	polarity	-0.08	Protein feature
255	2nd_stru_37	2nd_stru	-0.08	Protein feature

256	2nd_stru_7	2nd_stru	-0.08	Protein feature
257	AAC_10	AAC	-0.081	Protein feature
258	2nd_stru_29	2nd_stru	-0.081	Protein feature
259	EC_2	EC	-0.081	Protein feature
260	2nd_stru_32	2nd_stru	-0.083	Protein feature
261	Polarity_32	polarity	-0.083	Protein feature
262	AAC_15	AAC	-0.083	Protein feature
263	Polarity_47	polarity	-0.084	Protein feature
264	EC_18	EC	-0.085	Protein feature
265	2nd_stru_35	2nd_stru	-0.085	Protein feature
266	MV_44	MV	-0.086	Protein feature
267	MV_37	MV	-0.086	Protein feature
268	CD_27	CD	-0.088	Protein feature
269	MV_50	MV	-0.088	Protein feature
270	MV_19	MV	-0.089	Protein feature
271	EC_25	EC	-0.089	Protein feature
272	EC_7	EC	-0.089	Protein feature
273	MV_10	MV	-0.09	Protein feature
274	Polarity_9	polarity	-0.09	Protein feature
275	2nd_stru_15	2nd_stru	-0.09	Protein feature
276	MV_5	MV	-0.091	Protein feature
277	Polarity_44	polarity	-0.092	Protein feature
278	2nd_stru_18	2nd_stru	-0.092	Protein feature
279	Polarity_25	polarity	-0.093	Protein feature
280	EC_23	EC	-0.093	Protein feature
281	EC_6	EC	-0.094	Protein feature
282	Polarity_29	polarity	-0.094	Protein feature
283	2nd_stru_49	2nd_stru	-0.095	Protein feature
284	2nd_stru_42	2nd_stru	-0.096	Protein feature
285	EC_40	EC	-0.097	Protein feature
286	CD_12	CD	-0.097	Protein feature
287	EC_32	EC	-0.098	Protein feature
288	MV_17	MV	-0.099	Protein feature
289	2nd_stru_22	2nd_stru	-0.1	Protein feature
290	2nd_stru_45	2nd_stru	-0.102	Protein feature
291	2nd_stru_20	2nd_stru	-0.102	Protein feature
292	Polarity_5	polarity	-0.103	Protein feature
293	EC_13	EC	-0.104	Protein feature
294	2nd_stru_12	2nd_stru	-0.105	Protein feature
295	Polarity_18	polarity	-0.106	Protein feature
296	AAC_4	AAC	-0.107	Protein feature
297	CD_13	CD	-0.107	Protein feature
298	CD_19	CD	-0.108	Protein feature

299	MV_6	MV	-0.108	Protein feature
300	2nd_stru_50	2nd_stru	-0.109	Protein feature
301	EC_5	EC	-0.111	Protein feature
302	Polarity_22	polarity	-0.111	Protein feature
303	Polarity_2	polarity	-0.112	Protein feature
304	Polarity_31	polarity	-0.114	Protein feature
305	AAC_11	AAC	-0.115	Protein feature
306	Polarity_46	polarity	-0.116	Protein feature
307	CD_6	CD	-0.117	Protein feature
308	Polarity_6	polarity	-0.118	Protein feature
309	Polarity_49	polarity	-0.12	Protein feature
310	MV_4	MV	-0.121	Protein feature
311	AAC_8	AAC	-0.121	Protein feature
312	EC_9	EC	-0.123	Protein feature
313	Polarity_35	polarity	-0.123	Protein feature
314	2nd_stru_38	2nd_stru	-0.125	Protein feature
315	CD_4	CD	-0.126	Protein feature
316	CD_28	CD	-0.127	Protein feature
317	CD_37	CD	-0.131	Protein feature
318	EC_34	EC	-0.133	Protein feature
319	EC_37	EC	-0.134	Protein feature
320	CD_47	CD	-0.136	Protein feature
321	2nd_stru_23	2nd_stru	-0.139	Protein feature
322	Polarity_21	polarity	-0.144	Protein feature
323	EC_21	EC	-0.146	Protein feature
324	EC_47	EC	-0.15	Protein feature
325	MV_15	MV	-0.151	Protein feature
326	EC_10	EC	-0.153	Protein feature
327	AAC_12	AAC	-0.153	Protein feature
328	CD_2	CD	-0.156	Protein feature
329	AAC_9	AAC	-0.159	Protein feature
330	CD_44	CD	-0.161	Protein feature
331	MV_36	MV	-0.165	Protein feature
332	2nd_stru_27	2nd_stru	-0.166	Protein feature
333	CD_36	CD	-0.168	Protein feature
334	MV_47	MV	-0.171	Protein feature
335	Polarity_16	polarity	-0.172	Protein feature
336	CD_24	CD	-0.185	Protein feature
337	Polarity_13	polarity	-0.187	Protein feature
338	CD_30	CD	-0.198	Protein feature
339	AAC_13	AAC	-0.201	Protein feature
340	CD_3	CD	-0.211	Protein feature

Supplementary Material S3 The predicted results from the four types of algorithms along with the different number of features in the IFS feature sets.

(1) Dagging

Number of features	SN	SP	ACC	MCC
4	0	1	0.874107	---
5	0	1	0.874107	---
6	0	1	0.874107	---
7	0	1	0.874107	---
8	0	1	0.874107	---
9	0	1	0.874107	---
10	0	1	0.874107	---
11	0	1	0.874107	---
12	0	1	0.874107	---
13	0	1	0.874107	---
14	0	1	0.874107	---
15	0	1	0.874107	---
16	0	1	0.874107	---
17	0	1	0.874107	---
18	0	1	0.874107	---
19	0	1	0.874107	---
20	0	1	0.874107	---
21	0	1	0.874107	---
22	0	1	0.874107	---
23	0	1	0.874107	---
24	0	1	0.874107	---
25	0	1	0.874107	---
26	0	1	0.874107	---
27	0	1	0.874107	---
28	0	1	0.874107	---
29	0	1	0.874107	---
30	0	1	0.874107	---
31	0	1	0.874107	---
32	0	1	0.874107	---
33	0	1	0.874107	---
34	0	1	0.874107	---
35	0	1	0.874107	---
36	0	1	0.874107	---
37	0	1	0.874107	---
38	0	1	0.874107	---
39	0	1	0.874107	---
40	0	1	0.874107	---
41	0	1	0.874107	---

42	0	1	0.874107	---
43	0	1	0.874107	---
44	0	1	0.874107	---
45	0	1	0.874107	---
46	0	1	0.874107	---
47	0	1	0.874107	---
48	0	1	0.874107	---
49	0	1	0.874107	---
50	0	1	0.874107	---
51	0	1	0.874107	---
52	0	1	0.874107	---
53	0	1	0.874107	---
54	0	1	0.874107	---
55	0	1	0.874107	---
56	0	1	0.874107	---
57	0	1	0.874107	---
58	0	1	0.874107	---
59	0	1	0.874107	---
60	0	1	0.874107	---
61	0	1	0.874107	---
62	0	1	0.874107	---
63	0	1	0.874107	---
64	0	1	0.874107	---
65	0	1	0.874107	---
66	0	1	0.874107	---
67	0	1	0.874107	---
68	0	1	0.874107	---
69	0	1	0.874107	---
70	0	1	0.874107	---
71	0	1	0.874107	---
72	0	1	0.874107	---
73	0	1	0.874107	---
74	0	1	0.874107	---
75	0	1	0.874107	---
76	0	1	0.874107	---
77	0	1	0.874107	---
78	0.002364	1	0.874405	0.045465
79	0	1	0.874107	---
80	0	1	0.874107	---
81	0	0.99966	0.87381	-0.006548
82	0	1	0.874107	---
83	0	1	0.874107	---
84	0	1	0.874107	---

85	0	0.99966	0.87381	-0.006548
86	0.002364	1	0.874405	0.045465
87	0	1	0.874107	---
88	0	0.99966	0.87381	-0.006548
89	0.002364	1	0.874405	0.045465
90	0	1	0.874107	---
91	0	1	0.874107	---
92	0	1	0.874107	---
93	0	1	0.874107	---
94	0.002364	1	0.874405	0.045465
95	0.002364	1	0.874405	0.045465
96	0	0.999319	0.873512	-0.009262
97	0.002364	1	0.874405	0.045465
98	0.004728	1	0.874702	0.064307
99	0	0.999319	0.873512	-0.009262
100	0	1	0.874107	---
101	0	1	0.874107	---
102	0	1	0.874107	---
103	0	0.99966	0.87381	-0.006548
104	0.002364	0.999319	0.87381	0.018694
105	0	1	0.874107	---
106	0.004728	0.998979	0.87381	0.031899
107	0	1	0.874107	---
108	0	0.99966	0.87381	-0.006548
109	0.002364	1	0.874405	0.045465
110	0.004728	0.99966	0.874405	0.048732
111	0.002364	1	0.874405	0.045465
112	0	0.99966	0.87381	-0.006548
113	0	0.998979	0.873214	-0.011345
114	0.002364	1	0.874405	0.045465
115	0.002364	0.998979	0.873512	0.012916
116	0.002364	0.999319	0.87381	0.018694
117	0	1	0.874107	---
118	0.002364	0.99966	0.874107	0.027522
119	0.009456	0.997276	0.872917	0.037438
120	0.007092	0.998979	0.874107	0.047699
121	0	0.99966	0.87381	-0.006548
122	0.002364	0.999319	0.87381	0.018694
123	0	0.999319	0.873512	-0.009262
124	0.009456	0.999319	0.874702	0.068949
125	0.004728	0.999319	0.874107	0.038934
126	0	0.998979	0.873214	-0.011345
127	0.002364	0.999319	0.87381	0.018694

128	0.007092	0.998298	0.873512	0.036686
129	0.002364	1	0.874405	0.045465
130	0.004728	0.998979	0.87381	0.031899
131	0.002364	0.998979	0.873512	0.012916
132	0	0.998298	0.872619	-0.014651
133	0	0.99966	0.87381	-0.006548
134	0.002364	0.998979	0.873512	0.012916
135	0.007092	0.999319	0.874405	0.055174
136	0.004728	0.998979	0.87381	0.031899
137	0.007092	0.997276	0.872619	0.025368
138	0.002364	0.99966	0.874107	0.027522
139	0.002364	0.998979	0.873512	0.012916
140	0	0.998979	0.873214	-0.011345
141	0.01182	0.99966	0.875298	0.090199
142	0.002364	0.997617	0.872321	-0.000131
143	0.009456	0.998298	0.87381	0.049766
144	0	0.998298	0.872619	-0.014651
145	0.002364	0.997957	0.872619	0.002337
146	0.007092	0.997957	0.873214	0.032407
147	0.014184	0.998638	0.874702	0.078086
148	0.002364	0.998979	0.873512	0.012916
149	0.009456	0.997617	0.873214	0.041074
150	0.007092	0.998298	0.873512	0.036686
151	0.002364	0.998638	0.873214	0.008624
152	0.01182	0.999319	0.875	0.081043
153	0.007092	0.998979	0.874107	0.047699
154	0.004728	0.998298	0.873214	0.022013
155	0.004728	0.999319	0.874107	0.038934
156	0.007092	0.997276	0.872619	0.025368
157	0.007092	0.998298	0.873512	0.036686
158	0.002364	0.997957	0.872619	0.002337
159	0.01182	0.998638	0.874405	0.067124
160	0.007092	0.996595	0.872024	0.019703
161	0.009456	0.998298	0.87381	0.049766
162	0.002364	0.997617	0.872321	-0.000131
163	0.01182	0.995914	0.872024	0.036163
164	0.007092	0.997957	0.873214	0.032407
165	0.007092	0.998979	0.874107	0.047699
166	0.002364	0.998638	0.873214	0.008624
167	0.004728	0.998298	0.873214	0.022013
168	0.009456	0.997617	0.873214	0.041074
169	0.007092	0.998298	0.873512	0.036686
170	0.007092	0.998638	0.87381	0.04169

171	0.021277	0.996595	0.87381	0.079063
172	0.007092	0.998298	0.873512	0.036686
173	0.002364	0.997617	0.872321	-0.000131
174	0.009456	0.997617	0.873214	0.041074
175	0.009456	0.996595	0.872321	0.031164
176	0.007092	0.997617	0.872917	0.028676
177	0.009456	0.999319	0.874702	0.068949
178	0.01182	0.998979	0.874702	0.073503
179	0.007092	0.997276	0.872619	0.025368
180	0.009456	0.996595	0.872321	0.031164
181	0.014184	0.997276	0.873512	0.05902
182	0.004728	0.997957	0.872917	0.018277
183	0.014184	0.998298	0.874405	0.072486
184	0.018913	0.996595	0.873512	0.070474
185	0.007092	0.996255	0.871726	0.017236
186	0.009456	0.998979	0.874405	0.061367
187	0.01182	0.996255	0.872321	0.038911
188	0.01182	0.995914	0.872024	0.036163
189	0.009456	0.997276	0.872917	0.037438
190	0.01182	0.997957	0.87381	0.05678
191	0.002364	0.996936	0.871726	-0.004265
192	0.009456	0.997617	0.873214	0.041074
193	0.014184	0.996595	0.872917	0.051943
194	0.016548	0.998638	0.875	0.088192
195	0.01182	0.997617	0.873512	0.052477
196	0.018913	0.996255	0.873214	0.067099
197	0.007092	0.997957	0.873214	0.032407
198	0.009456	0.997617	0.873214	0.041074
199	0.007092	0.998298	0.873512	0.036686
200	0.021277	0.995914	0.873214	0.07236
201	0.014184	0.997617	0.87381	0.063058
202	0.018913	0.996936	0.87381	0.074098
203	0.014184	0.996255	0.872619	0.048808
204	0.007092	0.996255	0.871726	0.017236
205	0.009456	0.995914	0.871726	0.025879
206	0.016548	0.996595	0.873214	0.061453
207	0.016548	0.995914	0.872619	0.055134
208	0.007092	0.995574	0.871131	0.012846
209	0.01182	0.998298	0.874107	0.061616
210	0.016548	0.996595	0.873214	0.061453
211	0.018913	0.996936	0.87381	0.074098
212	0.014184	0.997276	0.873512	0.05902
213	0.023641	0.997957	0.875298	0.104073

214	0.01182	0.996595	0.872619	0.041875
215	0.016548	0.997276	0.87381	0.068791
216	0.021277	0.996936	0.874107	0.082765
217	0.016548	0.996936	0.873512	0.064976
218	0.014184	0.997617	0.87381	0.063058
219	0.018913	0.996255	0.873214	0.067099
220	0.021277	0.996595	0.87381	0.079063
221	0.009456	0.998638	0.874107	0.055094
222	0.01182	0.996255	0.872321	0.038911
223	0.014184	0.997617	0.87381	0.063058
224	0.009456	0.997957	0.873512	0.045146
225	0.009456	0.995914	0.871726	0.025879
226	0.021277	0.996595	0.87381	0.079063
227	0.016548	0.995914	0.872619	0.055134
228	0.01182	0.995914	0.872024	0.036163
229	0.026005	0.996936	0.874702	0.098931
230	0.014184	0.997276	0.873512	0.05902
231	0.009456	0.994552	0.870536	0.017287
232	0.023641	0.995914	0.873512	0.080431
233	0.016548	0.996936	0.873512	0.064976
234	0.016548	0.996936	0.873512	0.064976
235	0.01182	0.996936	0.872917	0.045092
236	0.01182	0.996255	0.872321	0.038911
237	0.016548	0.997276	0.87381	0.068791
238	0.014184	0.995574	0.872024	0.043169
239	0.021277	0.997957	0.875	0.095706
240	0.021277	0.997617	0.874702	0.091041
241	0.026005	0.994552	0.872619	0.07638
242	0.014184	0.998298	0.874405	0.072486
243	0.023641	0.996255	0.87381	0.083744
244	0.018913	0.995574	0.872619	0.060976
245	0.026005	0.995914	0.87381	0.088186
246	0.026005	0.994893	0.872917	0.079113
247	0.014184	0.995914	0.872321	0.045893
248	0.016548	0.996595	0.873214	0.061453
249	0.026005	0.995233	0.873214	0.081982
250	0.018913	0.996595	0.873512	0.070474
251	0.033097	0.995233	0.874107	0.103381
252	0.014184	0.996595	0.872917	0.051943
253	0.021277	0.997276	0.874405	0.086744
254	0.030733	0.996595	0.875	0.109948
255	0.014184	0.996936	0.873214	0.055333
256	0.021277	0.995914	0.873214	0.07236

257	0.023641	0.994893	0.872619	0.071542
258	0.028369	0.997617	0.875595	0.114957
259	0.018913	0.995914	0.872917	0.063941
260	0.016548	0.998298	0.874702	0.082556
261	0.018913	0.994893	0.872024	0.055542
262	0.023641	0.995914	0.873512	0.080431
263	0.023641	0.996255	0.87381	0.083744
264	0.016548	0.995233	0.872024	0.049592
265	0.014184	0.995233	0.871726	0.040614
266	0.030733	0.996595	0.875	0.109948
267	0.004728	0.994893	0.870238	-0.001773
268	0.021277	0.995914	0.873214	0.07236
269	0.026005	0.996936	0.874702	0.098931
270	0.016548	0.995574	0.872321	0.052277
271	0.016548	0.996255	0.872917	0.058183
272	0.023641	0.995914	0.873512	0.080431
273	0.028369	0.996936	0.875	0.106513
274	0.026005	0.995233	0.873214	0.081982
275	0.026005	0.996255	0.874107	0.091555
276	0.018913	0.995574	0.872619	0.060976
277	0.014184	0.995574	0.872024	0.043169
278	0.018913	0.996936	0.87381	0.074098
279	0.021277	0.996595	0.87381	0.079063
280	0.021277	0.997276	0.874405	0.086744
281	0.030733	0.996595	0.875	0.109948
282	0.021277	0.995233	0.872619	0.066424
283	0.007092	0.993871	0.869643	0.004056
284	0.014184	0.995914	0.872321	0.045893
285	0.018913	0.995574	0.872619	0.060976
286	0.018913	0.996595	0.873512	0.070474
287	0.018913	0.996595	0.873512	0.070474
288	0.028369	0.995574	0.87381	0.092422
289	0.023641	0.996255	0.87381	0.083744
290	0.021277	0.996595	0.87381	0.079063
291	0.016548	0.995574	0.872321	0.052277
292	0.030733	0.994893	0.873512	0.093511
293	0.021277	0.997617	0.874702	0.091041
294	0.023641	0.996936	0.874405	0.091028
295	0.021277	0.996255	0.873512	0.075604
296	0.023641	0.995233	0.872917	0.074347
297	0.018913	0.994893	0.872024	0.055542
298	0.026005	0.994212	0.872321	0.073773
299	0.016548	0.996595	0.873214	0.061453

300	0.021277	0.993871	0.871429	0.056283
301	0.023641	0.994893	0.872619	0.071542
302	0.026005	0.991488	0.86994	0.056363
303	0.018913	0.995914	0.872917	0.063941
304	0.026005	0.995574	0.873512	0.085001
305	0.021277	0.997276	0.874405	0.086744
306	0.035461	0.994893	0.874107	0.107041
307	0.018913	0.995914	0.872917	0.063941
308	0.014184	0.996595	0.872917	0.051943
309	0.023641	0.995574	0.873214	0.077305
310	0.021277	0.994212	0.871726	0.058635
311	0.033097	0.996255	0.875	0.113302
312	0.033097	0.996595	0.875298	0.116962
313	0.026005	0.994893	0.872917	0.079113
314	0.026005	0.995574	0.873512	0.085001
315	0.023641	0.993531	0.871429	0.061581
316	0.014184	0.994893	0.871429	0.038208
317	0.023641	0.995574	0.873214	0.077305
318	0.026005	0.995914	0.87381	0.088186
319	0.030733	0.997276	0.875595	0.117897
320	0.030733	0.994893	0.873512	0.093511
321	0.026005	0.996595	0.874405	0.095129
322	0.007092	0.997276	0.872619	0.025368
323	0.033097	0.995233	0.874107	0.103381
324	0.030733	0.995914	0.874405	0.102862
325	0.028369	0.997617	0.875595	0.114957
326	0.026005	0.996255	0.874107	0.091555
327	0.028369	0.996936	0.875	0.106513
328	0.021277	0.993871	0.871429	0.056283
329	0.026005	0.994212	0.872321	0.073773
330	0.023641	0.995574	0.873214	0.077305
331	0.030733	0.996595	0.875	0.109948
332	0.026005	0.994552	0.872619	0.07638
333	0.033097	0.996255	0.875	0.113302
334	0.040189	0.995233	0.875	0.122903
335	0.023641	0.994893	0.872619	0.071542
336	0.030733	0.993871	0.872619	0.085368
337	0.037825	0.994552	0.874107	0.110585
338	0.030733	0.994552	0.873214	0.090678
339	0.023641	0.995914	0.873512	0.080431
340	0.016548	0.995233	0.872024	0.049592

(2) NNA

Number of features	SN	SP	ACC	MCC
4	0.340426	0.91522	0.842857	0.263879
5	0.364066	0.912496	0.843452	0.279999
6	0.378251	0.911815	0.844643	0.291248
7	0.35461	0.912155	0.841964	0.270934
8	0.319149	0.902622	0.829167	0.222221
9	0.338061	0.906708	0.874107	0.246272
10	0.378251	0.913858	0.846429	0.295122
11	0.394799	0.922029	0.855655	0.325956
12	0.390071	0.919646	0.852976	0.316915
13	0.401891	0.919646	0.854464	0.327254
14	0.401891	0.92237	0.856845	0.332876
15	0.401891	0.922029	0.856548	0.332166
16	0.401891	0.921008	0.855655	0.330048
17	0.406619	0.918965	0.854464	0.329976
18	0.416076	0.918284	0.85506	0.33676
19	0.413712	0.918965	0.855357	0.336108
20	0.416076	0.917263	0.854167	0.334698
21	0.420804	0.912155	0.850298	0.328686
22	0.427896	0.911815	0.850893	0.334052
23	0.43026	0.911815	0.85119	0.336052
24	0.437352	0.912155	0.852381	0.342696
25	0.439716	0.911474	0.852083	0.343362
26	0.43026	0.912496	0.851786	0.337372
27	0.434988	0.912496	0.852381	0.341366
28	0.437352	0.913177	0.853274	0.34469
29	0.439716	0.910793	0.851488	0.342048
30	0.437352	0.910793	0.85119	0.340063
31	0.427896	0.910112	0.849405	0.330788
32	0.41844	0.911474	0.849405	0.325363
33	0.420804	0.910453	0.84881	0.325425
34	0.41844	0.910453	0.848512	0.323414
35	0.41844	0.911815	0.849702	0.326016
36	0.427896	0.913517	0.852381	0.337361
37	0.43026	0.912155	0.851488	0.336711
38	0.427896	0.912496	0.851488	0.33537
39	0.434988	0.910793	0.850893	0.338074
40	0.434988	0.911815	0.851786	0.340044
41	0.434988	0.912155	0.852083	0.340704
42	0.434988	0.910453	0.850595	0.337421
43	0.44208	0.909091	0.850298	0.340773

44	0.439716	0.910112	0.850893	0.340741
45	0.449173	0.916241	0.85744	0.360724
46	0.444444	0.915901	0.856548	0.356068
47	0.446809	0.915901	0.856845	0.358054
48	0.437352	0.917943	0.85744	0.354223
49	0.432624	0.916582	0.855655	0.347456
50	0.416076	0.912155	0.849702	0.324653
51	0.413712	0.911474	0.84881	0.321326
52	0.416076	0.912496	0.85	0.325309
53	0.41844	0.912155	0.85	0.326671
54	0.416076	0.912496	0.85	0.325309
55	0.432624	0.907388	0.847619	0.329639
56	0.43026	0.906708	0.846726	0.326385
57	0.43026	0.907729	0.847619	0.328287
58	0.420804	0.906027	0.84494	0.317153
59	0.423168	0.905005	0.844345	0.317281
60	0.427896	0.905005	0.84494	0.321262
61	0.434988	0.906708	0.847321	0.330355
62	0.43026	0.906708	0.846726	0.326385
63	0.432624	0.906708	0.847024	0.328371
64	0.432624	0.907048	0.847321	0.329004
65	0.411348	0.908069	0.845536	0.312892
66	0.406619	0.909431	0.846131	0.311388
67	0.411348	0.909772	0.847024	0.316075
68	0.406619	0.909431	0.846131	0.311388
69	0.406619	0.909431	0.846131	0.311388
70	0.41844	0.911815	0.849702	0.326016
71	0.420804	0.912496	0.850595	0.329344
72	0.41844	0.911474	0.849405	0.325363
73	0.41844	0.911815	0.849702	0.326016
74	0.41844	0.911474	0.849405	0.325363
75	0.423168	0.912496	0.850893	0.331356
76	0.427896	0.912836	0.851786	0.336032
77	0.437352	0.911474	0.851786	0.341376
78	0.432624	0.912155	0.851786	0.338709
79	0.43026	0.911134	0.850595	0.33474
80	0.43026	0.915901	0.854762	0.344083
81	0.425532	0.914198	0.852679	0.336689
82	0.425532	0.914198	0.852679	0.336689
83	0.425532	0.91556	0.853869	0.339383
84	0.427896	0.91556	0.854167	0.341395
85	0.432624	0.91556	0.854762	0.345408

86	0.43026	0.922029	0.860119	0.356667
87	0.43026	0.92271	0.860714	0.358108
88	0.432624	0.923391	0.861607	0.361578
89	0.44208	0.92237	0.861905	0.367446
90	0.444444	0.92271	0.8625	0.370173
91	0.446809	0.924413	0.864286	0.375834
92	0.446809	0.923391	0.863393	0.37363
93	0.446809	0.92237	0.8625	0.371446
94	0.446809	0.921689	0.861905	0.37
95	0.434988	0.924072	0.8625	0.365057
96	0.432624	0.924753	0.862798	0.364507
97	0.437352	0.923051	0.861905	0.364882
98	0.432624	0.924753	0.862798	0.364507
99	0.432624	0.925094	0.863095	0.365245
100	0.423168	0.928498	0.864881	0.364609
101	0.425532	0.927477	0.864286	0.364379
102	0.427896	0.926456	0.86369	0.364163
103	0.427896	0.925775	0.863095	0.362673
104	0.43026	0.926115	0.86369	0.365447
105	0.427896	0.927137	0.864286	0.365662
106	0.427896	0.925434	0.862798	0.361932
107	0.425532	0.927477	0.864286	0.364379
108	0.423168	0.927818	0.864286	0.363093
109	0.43026	0.926115	0.86369	0.365447
110	0.43026	0.927137	0.864583	0.367693
111	0.43026	0.927477	0.864881	0.368447
112	0.43026	0.925434	0.863095	0.36396
113	0.43026	0.92271	0.860714	0.358108
114	0.437352	0.92271	0.861607	0.364156
115	0.439716	0.919646	0.859226	0.359727
116	0.437352	0.920327	0.859524	0.359138
117	0.437352	0.925434	0.863988	0.370024
118	0.437352	0.925775	0.864286	0.370768
119	0.434988	0.926115	0.864286	0.369495
120	0.434988	0.927137	0.865179	0.371746
121	0.43026	0.930541	0.86756	0.375341
122	0.43026	0.934287	0.870833	0.38405
123	0.43026	0.933946	0.870536	0.383245
124	0.427896	0.932584	0.869048	0.37801
125	0.427896	0.931903	0.868452	0.37643
126	0.43026	0.931222	0.868155	0.376901
127	0.432624	0.92986	0.867262	0.375825

128	0.434988	0.927818	0.865774	0.373258
129	0.434988	0.929179	0.866964	0.376312
130	0.43026	0.928498	0.865774	0.370723
131	0.432624	0.92986	0.867262	0.375825
132	0.432624	0.928498	0.866071	0.372754
133	0.434988	0.92952	0.867262	0.377082
134	0.437352	0.931563	0.869345	0.383782
135	0.437352	0.92986	0.867857	0.37988
136	0.437352	0.92952	0.86756	0.379107
137	0.44208	0.930541	0.869048	0.385476
138	0.449173	0.932925	0.872024	0.397048
139	0.446809	0.932584	0.871429	0.394238
140	0.451537	0.933606	0.872917	0.400663
141	0.444444	0.933265	0.871726	0.39382
142	0.444444	0.933265	0.871726	0.39382
143	0.453901	0.932925	0.872619	0.401062
144	0.456265	0.933946	0.87381	0.405479
145	0.456265	0.933946	0.87381	0.405479
146	0.456265	0.933606	0.873512	0.404671
147	0.458629	0.935308	0.875298	0.41074
148	0.458629	0.934627	0.874702	0.409104
149	0.458629	0.932925	0.873214	0.405061
150	0.458629	0.932925	0.873214	0.405061
151	0.458629	0.932925	0.873214	0.405061
152	0.458629	0.933606	0.87381	0.40667
153	0.460993	0.935649	0.875893	0.41356
154	0.458629	0.933606	0.87381	0.40667
155	0.456265	0.933265	0.873214	0.403866
156	0.444444	0.931903	0.870536	0.390635
157	0.444444	0.931563	0.870238	0.389845
158	0.446809	0.930201	0.869345	0.388723
159	0.446809	0.930882	0.86994	0.390286
160	0.449173	0.92986	0.869345	0.389953
161	0.449173	0.929179	0.86875	0.388404
162	0.449173	0.928839	0.868452	0.387634
163	0.449173	0.928158	0.867857	0.3861
164	0.449173	0.927818	0.86756	0.385336
165	0.449173	0.927818	0.86756	0.385336
166	0.446809	0.928158	0.86756	0.384095
167	0.444444	0.928158	0.867262	0.382087
168	0.444444	0.928498	0.86756	0.382852
169	0.446809	0.928498	0.867857	0.38486

170	0.453901	0.925775	0.866369	0.384797
171	0.453901	0.925434	0.866071	0.384049
172	0.456265	0.925775	0.866667	0.386787
173	0.456265	0.925775	0.866667	0.386787
174	0.453901	0.926115	0.866667	0.385547
175	0.456265	0.925775	0.866667	0.386787
176	0.456265	0.925775	0.866667	0.386787
177	0.456265	0.925094	0.866071	0.385292
178	0.451537	0.924753	0.865179	0.380568
179	0.456265	0.924753	0.865774	0.384548
180	0.456265	0.924753	0.865774	0.384548
181	0.456265	0.924753	0.865774	0.384548
182	0.456265	0.925094	0.866071	0.385292
183	0.456265	0.925094	0.866071	0.385292
184	0.456265	0.925434	0.866369	0.386039
185	0.456265	0.925094	0.866071	0.385292
186	0.451537	0.924753	0.865179	0.380568
187	0.456265	0.924753	0.865774	0.384548
188	0.456265	0.925094	0.866071	0.385292
189	0.458629	0.924753	0.866071	0.386534
190	0.460993	0.924413	0.866071	0.387772
191	0.460993	0.924413	0.866071	0.387772
192	0.460993	0.924413	0.866071	0.387772
193	0.460993	0.924753	0.866369	0.388515
194	0.463357	0.925775	0.86756	0.392737
195	0.465721	0.925775	0.867857	0.394714
196	0.465721	0.925775	0.867857	0.394714
197	0.460993	0.925434	0.866964	0.390008
198	0.465721	0.925775	0.867857	0.394714
199	0.465721	0.925094	0.867262	0.393215
200	0.460993	0.924413	0.866071	0.387772
201	0.460993	0.923391	0.865179	0.385558
202	0.465721	0.924753	0.866964	0.392469
203	0.465721	0.924753	0.866964	0.392469
204	0.465721	0.924753	0.866964	0.392469
205	0.465721	0.924413	0.866667	0.391725
206	0.465721	0.924413	0.866667	0.391725
207	0.465721	0.924072	0.866369	0.390983
208	0.468085	0.924413	0.866964	0.393696
209	0.468085	0.924413	0.866964	0.393696
210	0.468085	0.924413	0.866964	0.393696
211	0.463357	0.924413	0.866369	0.38975

212	0.463357	0.924753	0.866667	0.390494
213	0.465721	0.924413	0.866667	0.391725
214	0.465721	0.924413	0.866667	0.391725
215	0.468085	0.924753	0.867262	0.39444
216	0.470449	0.924413	0.867262	0.395664
217	0.470449	0.924413	0.867262	0.395664
218	0.470449	0.924413	0.867262	0.395664
219	0.470449	0.924753	0.86756	0.396409
220	0.470449	0.924072	0.866964	0.394921
221	0.470449	0.924413	0.867262	0.395664
222	0.472813	0.924413	0.86756	0.397629
223	0.470449	0.924413	0.867262	0.395664
224	0.468085	0.923732	0.866369	0.392214
225	0.465721	0.923051	0.865476	0.388772
226	0.465721	0.923051	0.865476	0.388772
227	0.465721	0.923051	0.865476	0.388772
228	0.465721	0.923391	0.865774	0.389507
229	0.465721	0.923391	0.865774	0.389507
230	0.465721	0.923391	0.865774	0.389507
231	0.468085	0.923391	0.866071	0.391476
232	0.468085	0.923391	0.866071	0.391476
233	0.468085	0.923391	0.866071	0.391476
234	0.470449	0.923732	0.866667	0.394181
235	0.470449	0.923732	0.866667	0.394181
236	0.475177	0.924072	0.86756	0.398846
237	0.475177	0.924072	0.86756	0.398846
238	0.475177	0.924072	0.86756	0.398846
239	0.475177	0.924072	0.86756	0.398846
240	0.475177	0.924072	0.86756	0.398846
241	0.475177	0.923732	0.867262	0.398105
242	0.475177	0.923732	0.867262	0.398105
243	0.475177	0.923391	0.866964	0.397366
244	0.475177	0.923391	0.866964	0.397366
245	0.475177	0.923051	0.866667	0.396629
246	0.475177	0.92271	0.866369	0.395894
247	0.475177	0.923051	0.866667	0.396629
248	0.475177	0.923391	0.866964	0.397366
249	0.475177	0.923391	0.866964	0.397366
250	0.477541	0.924753	0.868452	0.402295
251	0.477541	0.924753	0.868452	0.402295
252	0.475177	0.924753	0.868155	0.400336
253	0.475177	0.926115	0.869345	0.403342

254	0.475177	0.926115	0.869345	0.403342
255	0.475177	0.926115	0.869345	0.403342
256	0.475177	0.925775	0.869048	0.402587
257	0.475177	0.925775	0.869048	0.402587
258	0.475177	0.925094	0.868452	0.401084
259	0.475177	0.925094	0.868452	0.401084
260	0.475177	0.925094	0.868452	0.401084
261	0.475177	0.924413	0.867857	0.39959
262	0.475177	0.924413	0.867857	0.39959
263	0.475177	0.924753	0.868155	0.400336
264	0.475177	0.924413	0.867857	0.39959
265	0.472813	0.924413	0.86756	0.397629
266	0.472813	0.924413	0.86756	0.397629
267	0.472813	0.925094	0.868155	0.399122
268	0.472813	0.925094	0.868155	0.399122
269	0.470449	0.925094	0.867857	0.397156
270	0.470449	0.925094	0.867857	0.397156
271	0.470449	0.925094	0.867857	0.397156
272	0.470449	0.925094	0.867857	0.397156
273	0.470449	0.925094	0.867857	0.397156
274	0.470449	0.925434	0.868155	0.397905
275	0.470449	0.925434	0.868155	0.397905
276	0.468085	0.925094	0.86756	0.395187
277	0.470449	0.925434	0.868155	0.397905
278	0.463357	0.925094	0.866964	0.391239
279	0.463357	0.924753	0.866667	0.390494
280	0.463357	0.925094	0.866964	0.391239
281	0.458629	0.924753	0.866071	0.386534
282	0.456265	0.924753	0.865774	0.384548
283	0.456265	0.924413	0.865476	0.383807
284	0.456265	0.924072	0.865179	0.383067
285	0.453901	0.923732	0.864583	0.380343
286	0.453901	0.923732	0.864583	0.380343
287	0.453901	0.923732	0.864583	0.380343
288	0.453901	0.923732	0.864583	0.380343
289	0.456265	0.923732	0.864881	0.38233
290	0.456265	0.923391	0.864583	0.381595
291	0.458629	0.923391	0.864881	0.383578
292	0.458629	0.923391	0.864881	0.383578
293	0.458629	0.923391	0.864881	0.383578
294	0.460993	0.923391	0.865179	0.385558
295	0.460993	0.923732	0.865476	0.386294

296	0.460993	0.923732	0.865476	0.386294
297	0.460993	0.923732	0.865476	0.386294
298	0.460993	0.923732	0.865476	0.386294
299	0.460993	0.923732	0.865476	0.386294
300	0.460993	0.923732	0.865476	0.386294
301	0.460993	0.923391	0.865179	0.385558
302	0.458629	0.923391	0.864881	0.383578
303	0.458629	0.923051	0.864583	0.382845
304	0.458629	0.923051	0.864583	0.382845
305	0.458629	0.923051	0.864583	0.382845
306	0.458629	0.92237	0.863988	0.381385
307	0.458629	0.92237	0.863988	0.381385
308	0.458629	0.92237	0.863988	0.381385
309	0.456265	0.92237	0.86369	0.379404
310	0.456265	0.92237	0.86369	0.379404
311	0.458629	0.921689	0.863393	0.379934
312	0.458629	0.921689	0.863393	0.379934
313	0.456265	0.921348	0.862798	0.377232
314	0.456265	0.921348	0.862798	0.377232
315	0.456265	0.921008	0.8625	0.376512
316	0.456265	0.921008	0.8625	0.376512
317	0.456265	0.920667	0.862202	0.375795
318	0.456265	0.920667	0.862202	0.375795
319	0.453901	0.920667	0.861905	0.373813
320	0.453901	0.920667	0.861905	0.373813
321	0.453901	0.920667	0.861905	0.373813
322	0.451537	0.920327	0.86131	0.371114
323	0.451537	0.920327	0.86131	0.371114
324	0.451537	0.920327	0.86131	0.371114
325	0.451537	0.920327	0.86131	0.371114
326	0.449173	0.920327	0.861012	0.369126
327	0.449173	0.920327	0.861012	0.369126
328	0.446809	0.920327	0.860714	0.367135
329	0.446809	0.920327	0.860714	0.367135
330	0.446809	0.920327	0.860714	0.367135
331	0.446809	0.920327	0.860714	0.367135
332	0.446809	0.920327	0.860714	0.367135
333	0.446809	0.920327	0.860714	0.367135
334	0.446809	0.920327	0.860714	0.367135
335	0.446809	0.920327	0.860714	0.367135
336	0.444444	0.919986	0.860119	0.364431
337	0.444444	0.919986	0.860119	0.364431

338	0.444444	0.919986	0.860119	0.364431
339	0.444444	0.919986	0.860119	0.364431
340	0.444444	0.919986	0.860119	0.364431

(3) RF

Number of features	SN	SP	ACC	MCC
4	0.345154	0.934287	0.860119	0.307777
5	0.349882	0.930201	0.857143	0.303008
6	0.359338	0.934968	0.8625	0.322468
7	0.338061	0.937692	0.862202	0.309048
8	0.312057	0.937011	0.858333	0.282656
9	0.300236	0.943139	0.874107	0.285719
10	0.312057	0.942458	0.863095	0.29564
11	0.300236	0.948927	0.867262	0.300525
12	0.314421	0.950289	0.870238	0.318233
13	0.288416	0.946544	0.86369	0.282467
14	0.340426	0.952673	0.875595	0.350089
15	0.321513	0.953013	0.873512	0.332761
16	0.34279	0.953354	0.876488	0.354295
17	0.356974	0.948927	0.874405	0.355329
18	0.333333	0.953013	0.875	0.344244
19	0.340426	0.952673	0.875595	0.350089
20	0.326241	0.954716	0.875595	0.342269
21	0.316785	0.95097	0.871131	0.32242
22	0.349882	0.958461	0.881845	0.376266
23	0.349882	0.958801	0.882143	0.37732
24	0.349882	0.959823	0.883036	0.380513
25	0.316785	0.95778	0.877083	0.342087
26	0.35461	0.95744	0.881548	0.377617
27	0.323877	0.953694	0.874405	0.337013
28	0.361702	0.956418	0.881548	0.381202
29	0.328605	0.963909	0.883929	0.373213
30	0.338061	0.960844	0.88244	0.372414
31	0.326241	0.958801	0.879167	0.354512
32	0.335697	0.956078	0.877976	0.355448
33	0.349882	0.960163	0.883333	0.381587
34	0.335697	0.959823	0.88125	0.366895
35	0.359338	0.959823	0.884226	0.389467
36	0.34279	0.95744	0.88006	0.366365
37	0.34279	0.962206	0.884226	0.38136
38	0.338061	0.961866	0.883333	0.375697
39	0.328605	0.962206	0.88244	0.367607

40	0.352246	0.960163	0.883631	0.383835
41	0.323877	0.959142	0.879167	0.353249
42	0.352246	0.962887	0.886012	0.392632
43	0.335697	0.959142	0.880655	0.364768
44	0.356974	0.959823	0.883929	0.387238
45	0.335697	0.961525	0.882738	0.372309
46	0.359338	0.958121	0.882738	0.384163
47	0.359338	0.961525	0.885714	0.394902
48	0.333333	0.965611	0.886012	0.38359
49	0.345154	0.960163	0.882738	0.377072
50	0.330969	0.962887	0.883333	0.372141
51	0.330969	0.965271	0.885417	0.380119
52	0.319149	0.968335	0.886607	0.379191
53	0.359338	0.958121	0.882738	0.384163
54	0.304965	0.960163	0.877679	0.337638
55	0.319149	0.963909	0.882738	0.363899
56	0.375887	0.961525	0.887798	0.410355
57	0.312057	0.962887	0.880952	0.353468
58	0.307329	0.960504	0.878274	0.341082
59	0.321513	0.968335	0.886905	0.381534
60	0.328605	0.965271	0.885119	0.377807
61	0.34279	0.964249	0.886012	0.388119
62	0.319149	0.960844	0.88006	0.353932
63	0.307329	0.969016	0.885714	0.369812
64	0.321513	0.964249	0.883333	0.367376
65	0.312057	0.96459	0.88244	0.359117
66	0.338061	0.965952	0.886905	0.389352
67	0.326241	0.965611	0.885119	0.376651
68	0.319149	0.962887	0.881845	0.360523
69	0.321513	0.96459	0.883631	0.36852
70	0.319149	0.965271	0.883929	0.368486
71	0.328605	0.964249	0.884226	0.374352
72	0.304965	0.96493	0.881845	0.353134
73	0.314421	0.967654	0.885417	0.372056
74	0.338061	0.965271	0.88631	0.387016
75	0.326241	0.963228	0.883036	0.368636
76	0.316785	0.962547	0.88125	0.357066
77	0.340426	0.966973	0.888095	0.395192
78	0.321513	0.962547	0.881845	0.361747
79	0.330969	0.963568	0.883929	0.37439
80	0.309693	0.963568	0.88125	0.353342
81	0.333333	0.96459	0.885119	0.380114

82	0.312057	0.967995	0.885417	0.370899
83	0.323877	0.965271	0.884524	0.373161
84	0.314421	0.965271	0.883333	0.363783
85	0.3026	0.963568	0.880357	0.346195
86	0.3026	0.967654	0.883929	0.360152
87	0.34279	0.963568	0.885417	0.385843
88	0.321513	0.961185	0.880655	0.357351
89	0.312057	0.967314	0.884821	0.368489
90	0.307329	0.962547	0.88006	0.347618
91	0.333333	0.962887	0.883631	0.374444
92	0.347518	0.962547	0.885119	0.387005
93	0.304965	0.96493	0.881845	0.353134
94	0.330969	0.966973	0.886905	0.386006
95	0.309693	0.963909	0.881548	0.354471
96	0.307329	0.966973	0.883929	0.362541
97	0.3026	0.969697	0.885714	0.367505
98	0.340426	0.966633	0.887798	0.394001
99	0.288416	0.970378	0.884524	0.35545
100	0.295508	0.961525	0.877679	0.332359
101	0.309693	0.967314	0.884524	0.366116
102	0.312057	0.971399	0.888393	0.383389
103	0.319149	0.963909	0.882738	0.363899
104	0.309693	0.967314	0.884524	0.366116
105	0.309693	0.963568	0.88125	0.353342
106	0.333333	0.968335	0.888393	0.393142
107	0.307329	0.967995	0.884821	0.366145
108	0.27896	0.964249	0.877976	0.32411
109	0.314421	0.966633	0.884524	0.36847
110	0.316785	0.967654	0.885714	0.374414
111	0.300236	0.962206	0.878869	0.339353
112	0.297872	0.966633	0.88244	0.351758
113	0.300236	0.96459	0.880952	0.347196
114	0.316785	0.961866	0.880655	0.354859
115	0.300236	0.968335	0.884226	0.360169
116	0.300236	0.969016	0.884821	0.362619
117	0.307329	0.965611	0.882738	0.357833
118	0.307329	0.963909	0.88125	0.352095
119	0.307329	0.970718	0.887202	0.376073
120	0.312057	0.96459	0.88244	0.359117
121	0.319149	0.966292	0.884821	0.371994
122	0.295508	0.96459	0.880357	0.342373
123	0.316785	0.963568	0.882143	0.360422

124	0.307329	0.963909	0.88125	0.352095
125	0.312057	0.963909	0.881845	0.356839
126	0.297872	0.963909	0.88006	0.342518
127	0.321513	0.96459	0.883631	0.36852
128	0.312057	0.967654	0.885119	0.36969
129	0.314421	0.972421	0.889583	0.389653
130	0.307329	0.96493	0.882143	0.355518
131	0.3026	0.968676	0.884821	0.363796
132	0.297872	0.970037	0.885417	0.363936
133	0.328605	0.969356	0.88869	0.392214
134	0.300236	0.966973	0.883036	0.355355
135	0.295508	0.966633	0.882143	0.349339
136	0.288416	0.965952	0.880655	0.339689
137	0.300236	0.963568	0.88006	0.343797
138	0.319149	0.962547	0.881548	0.35941
139	0.314421	0.966973	0.884821	0.369659
140	0.307329	0.965952	0.883036	0.359
141	0.312057	0.968676	0.886012	0.373338
142	0.314421	0.967995	0.885714	0.373265
143	0.316785	0.965611	0.883929	0.367301
144	0.3026	0.966973	0.883333	0.357758
145	0.288416	0.969356	0.883631	0.351701
146	0.316785	0.966973	0.885119	0.372016
147	0.27896	0.964249	0.877976	0.32411
148	0.307329	0.966633	0.883631	0.361354
149	0.321513	0.967314	0.886012	0.377904
150	0.319149	0.970378	0.888393	0.386641
151	0.286052	0.964249	0.878869	0.331498
152	0.300236	0.963909	0.880357	0.344924
153	0.286052	0.965271	0.879762	0.334922
154	0.300236	0.969356	0.885119	0.363855
155	0.286052	0.962887	0.877679	0.327024
156	0.300236	0.966292	0.88244	0.35299
157	0.276596	0.968335	0.88125	0.335661
158	0.293144	0.967314	0.88244	0.349288
159	0.293144	0.969016	0.883929	0.355353
160	0.295508	0.971399	0.88631	0.366594
161	0.288416	0.971399	0.885417	0.359271
162	0.300236	0.97208	0.8875	0.374024
163	0.271868	0.966973	0.879464	0.325871
164	0.276596	0.967654	0.880655	0.333251
165	0.293144	0.970718	0.885417	0.361606

166	0.295508	0.969356	0.884524	0.359018
167	0.3026	0.96493	0.881548	0.350741
168	0.281324	0.966292	0.88006	0.33348
169	0.27896	0.968676	0.881845	0.339367
170	0.281324	0.965952	0.879762	0.332313
171	0.276596	0.967314	0.880357	0.332057
172	0.271868	0.96493	0.877679	0.31891
173	0.255319	0.967995	0.878274	0.311642
174	0.286052	0.965611	0.88006	0.336077
175	0.267139	0.967314	0.879167	0.322018
176	0.295508	0.967654	0.883036	0.352915
177	0.274232	0.970037	0.88244	0.339318
178	0.274232	0.967654	0.880357	0.330754
179	0.3026	0.962547	0.879464	0.34285
180	0.264775	0.967995	0.879464	0.321873
181	0.300236	0.964249	0.880655	0.346056
182	0.274232	0.97208	0.884226	0.346972
183	0.304965	0.97208	0.888095	0.37883
184	0.276596	0.96493	0.878274	0.323899
185	0.29078	0.965271	0.880357	0.339812
186	0.271868	0.967995	0.880357	0.329448
187	0.27896	0.969016	0.882143	0.340592
188	0.262411	0.971399	0.882143	0.331728
189	0.300236	0.963568	0.88006	0.343797
190	0.297872	0.973442	0.888393	0.376888
191	0.274232	0.968335	0.880952	0.333162
192	0.267139	0.967314	0.879167	0.322018
193	0.248227	0.968335	0.877679	0.305064
194	0.271868	0.970037	0.882143	0.336808
195	0.281324	0.975826	0.888393	0.369319
196	0.262411	0.970378	0.88125	0.327923
197	0.295508	0.968676	0.883929	0.356555
198	0.288416	0.970718	0.884821	0.356716
199	0.252955	0.965611	0.875893	0.300871
200	0.276596	0.97208	0.884524	0.349476
201	0.271868	0.967654	0.88006	0.328248
202	0.257683	0.967995	0.878571	0.314214
203	0.283688	0.966633	0.880655	0.337122
204	0.281324	0.968676	0.882143	0.341848
205	0.264775	0.968676	0.88006	0.32429
206	0.297872	0.970378	0.885714	0.365194
207	0.262411	0.97208	0.882738	0.334308

208	0.255319	0.967654	0.877976	0.310448
209	0.264775	0.968676	0.88006	0.32429
210	0.281324	0.967654	0.88125	0.338218
211	0.281324	0.96493	0.878869	0.328854
212	0.264775	0.968676	0.88006	0.32429
213	0.269504	0.969697	0.881548	0.333042
214	0.286052	0.965611	0.88006	0.336077
215	0.276596	0.969016	0.881845	0.338101
216	0.295508	0.962887	0.878869	0.336748
217	0.309693	0.969016	0.886012	0.372194
218	0.307329	0.97174	0.888095	0.379922
219	0.260047	0.966292	0.877381	0.310874
220	0.260047	0.965271	0.876488	0.30742
221	0.255319	0.964249	0.875	0.298919
222	0.257683	0.969356	0.879762	0.319069
223	0.288416	0.970718	0.884821	0.356716
224	0.3026	0.969697	0.885714	0.367505
225	0.241135	0.97208	0.88006	0.310879
226	0.262411	0.971059	0.881845	0.330451
227	0.245863	0.969356	0.878274	0.306089
228	0.27896	0.970378	0.883333	0.345568
229	0.264775	0.97208	0.883036	0.33686
230	0.269504	0.970037	0.881845	0.334288
231	0.267139	0.970037	0.881548	0.331758
232	0.252955	0.966973	0.877083	0.305506
233	0.262411	0.967654	0.878869	0.318133
234	0.243499	0.970718	0.879167	0.308424
235	0.27896	0.965611	0.879167	0.328678
236	0.281324	0.970037	0.883333	0.346796
237	0.255319	0.971399	0.88125	0.324016
238	0.250591	0.968676	0.878274	0.308874
239	0.264775	0.970718	0.881845	0.331733
240	0.262411	0.966292	0.877679	0.313422
241	0.245863	0.968335	0.877381	0.302451
242	0.248227	0.970718	0.879762	0.313675
243	0.286052	0.973442	0.886905	0.364679
244	0.248227	0.969356	0.878571	0.308705
245	0.248227	0.973102	0.881845	0.322709
246	0.262411	0.971399	0.882143	0.331728
247	0.255319	0.975485	0.884821	0.340029
248	0.260047	0.972761	0.883036	0.33436
249	0.243499	0.969016	0.877679	0.302242

250	0.22695	0.971059	0.877381	0.290953
251	0.252955	0.968335	0.878274	0.31026
252	0.250591	0.971399	0.880655	0.318823
253	0.250591	0.97208	0.88125	0.321396
254	0.255319	0.967995	0.878274	0.311642
255	0.257683	0.966292	0.877083	0.308317
256	0.271868	0.969356	0.881548	0.334323
257	0.255319	0.969697	0.879762	0.317726
258	0.274232	0.966292	0.879167	0.326025
259	0.269504	0.971399	0.883036	0.339352
260	0.255319	0.968335	0.878571	0.312843
261	0.274232	0.969697	0.882143	0.338071
262	0.238771	0.970378	0.878274	0.301878
263	0.255319	0.970718	0.880655	0.321474
264	0.241135	0.967995	0.876488	0.295999
265	0.276596	0.970718	0.883333	0.344339
266	0.262411	0.969016	0.88006	0.322965
267	0.264775	0.966633	0.878274	0.317129
268	0.264775	0.966292	0.877976	0.315961
269	0.243499	0.969356	0.877976	0.303462
270	0.255319	0.971399	0.88125	0.324016
271	0.250591	0.970037	0.879464	0.313782
272	0.243499	0.972761	0.880952	0.316129
273	0.234043	0.969356	0.876786	0.292848
274	0.255319	0.969697	0.879762	0.317726
275	0.262411	0.970378	0.88125	0.327923
276	0.257683	0.967654	0.878274	0.31302
277	0.274232	0.970378	0.882738	0.340573
278	0.229314	0.969016	0.875893	0.286264
279	0.262411	0.966973	0.878274	0.315763
280	0.248227	0.970037	0.879167	0.311173
281	0.267139	0.972761	0.883929	0.34202
282	0.255319	0.970378	0.880357	0.320217
283	0.238771	0.970037	0.877976	0.300636
284	0.27896	0.968676	0.881845	0.339367
285	0.229314	0.972421	0.878869	0.298775
286	0.22695	0.970378	0.876786	0.288455
287	0.234043	0.970718	0.877976	0.297791
288	0.245863	0.972421	0.880952	0.317458
289	0.236407	0.971399	0.878869	0.302992
290	0.252955	0.96493	0.875298	0.298597
291	0.241135	0.97174	0.879762	0.309591

292	0.245863	0.971399	0.88006	0.313589
293	0.264775	0.970037	0.88125	0.329219
294	0.260047	0.969016	0.879762	0.320409
295	0.236407	0.970378	0.877976	0.299216
296	0.234043	0.967654	0.875298	0.286856
297	0.248227	0.966633	0.87619	0.299152
298	0.236407	0.967995	0.875893	0.290703
299	0.229314	0.975145	0.88125	0.309463
300	0.243499	0.968335	0.877083	0.299827
301	0.243499	0.969016	0.877679	0.302242
302	0.283688	0.969697	0.883333	0.348021
303	0.248227	0.969356	0.878571	0.308705
304	0.238771	0.971059	0.878869	0.304389
305	0.267139	0.974123	0.885119	0.347363
306	0.269504	0.97208	0.883631	0.341935
307	0.238771	0.968676	0.876786	0.29575
308	0.224586	0.967995	0.874405	0.277272
309	0.248227	0.969356	0.878571	0.308705
310	0.274232	0.970037	0.88244	0.339318
311	0.271868	0.978209	0.889286	0.369392
312	0.231678	0.966292	0.87381	0.279538
313	0.238771	0.972761	0.880357	0.310823
314	0.245863	0.973102	0.881548	0.320083
315	0.236407	0.973442	0.880655	0.310791
316	0.22695	0.971059	0.877381	0.290953
317	0.236407	0.971399	0.878869	0.302992
318	0.255319	0.970378	0.880357	0.320217
319	0.222222	0.973783	0.879167	0.295845
320	0.269504	0.965952	0.878274	0.319848
321	0.238771	0.972421	0.88006	0.309518
322	0.208038	0.969356	0.873512	0.262725
323	0.245863	0.965611	0.875	0.293091
324	0.217494	0.968676	0.874107	0.271445
325	0.245863	0.97208	0.880655	0.316159
326	0.250591	0.967995	0.877679	0.306468
327	0.252955	0.970037	0.879762	0.316379
328	0.234043	0.967654	0.875298	0.286856
329	0.250591	0.969697	0.879167	0.312542
330	0.229314	0.97208	0.878571	0.297483
331	0.250591	0.971059	0.880357	0.31755
332	0.210402	0.967995	0.872619	0.26078
333	0.222222	0.97208	0.877679	0.289306

334	0.236407	0.973102	0.880357	0.309468
335	0.219858	0.97174	0.877083	0.285279
336	0.208038	0.969697	0.87381	0.263931
337	0.222222	0.971399	0.877083	0.286758
338	0.234043	0.973783	0.880655	0.309441
339	0.245863	0.971059	0.879762	0.312317
340	0.252955	0.967654	0.877679	0.307868

(4) SVM

Number of features	SN	SP	ACC	MCC
4	0	1	0.874107	---
5	0	1	0.874107	---
6	0	1	0.874107	---
7	0	1	0.874107	---
8	0	1	0.874107	---
9	0	1	0.874107	---
10	0	1	0.874107	---
11	0	1	0.874107	---
12	0	1	0.874107	---
13	0	1	0.874107	---
14	0	1	0.874107	---
15	0	1	0.874107	---
16	0	1	0.874107	---
17	0	1	0.874107	---
18	0	1	0.874107	---
19	0	1	0.874107	---
20	0	1	0.874107	---
21	0	1	0.874107	---
22	0	1	0.874107	---
23	0	1	0.874107	---
24	0	1	0.874107	---
25	0	1	0.874107	---
26	0	1	0.874107	---
27	0	1	0.874107	---
28	0	1	0.874107	---
29	0	1	0.874107	---
30	0	1	0.874107	---
31	0	1	0.874107	---
32	0	1	0.874107	---
33	0	1	0.874107	---
34	0	1	0.874107	---
35	0	1	0.874107	---

36	0	1	0.874107	---
37	0	1	0.874107	---
38	0	1	0.874107	---
39	0	1	0.874107	---
40	0	1	0.874107	---
41	0	1	0.874107	---
42	0	1	0.874107	---
43	0	1	0.874107	---
44	0	1	0.874107	---
45	0	1	0.874107	---
46	0	1	0.874107	---
47	0	1	0.874107	---
48	0	1	0.874107	---
49	0	1	0.874107	---
50	0	1	0.874107	---
51	0	1	0.874107	---
52	0	1	0.874107	---
53	0	1	0.874107	---
54	0	1	0.874107	---
55	0	1	0.874107	---
56	0	1	0.874107	---
57	0	1	0.874107	---
58	0	1	0.874107	---
59	0	1	0.874107	---
60	0	1	0.874107	---
61	0	1	0.874107	---
62	0	1	0.874107	---
63	0	1	0.874107	---
64	0	1	0.874107	---
65	0	1	0.874107	---
66	0	1	0.874107	---
67	0	1	0.874107	---
68	0	1	0.874107	---
69	0	1	0.874107	---
70	0	1	0.874107	---
71	0	1	0.874107	---
72	0	1	0.874107	---
73	0	1	0.874107	---
74	0	1	0.874107	---
75	0	1	0.874107	---
76	0	1	0.874107	---
77	0	1	0.874107	---

78	0	1	0.874107	---
79	0	1	0.874107	---
80	0	1	0.874107	---
81	0	1	0.874107	---
82	0	1	0.874107	---
83	0	1	0.874107	---
84	0	1	0.874107	---
85	0	1	0.874107	---
86	0	1	0.874107	---
87	0	1	0.874107	---
88	0	1	0.874107	---
89	0	1	0.874107	---
90	0	1	0.874107	---
91	0	1	0.874107	---
92	0	1	0.874107	---
93	0	1	0.874107	---
94	0	1	0.874107	---
95	0	1	0.874107	---
96	0	1	0.874107	---
97	0	1	0.874107	---
98	0	1	0.874107	---
99	0	1	0.874107	---
100	0	1	0.874107	---
101	0	1	0.874107	---
102	0	1	0.874107	---
103	0	1	0.874107	---
104	0	1	0.874107	---
105	0	1	0.874107	---
106	0	1	0.874107	---
107	0	1	0.874107	---
108	0	1	0.874107	---
109	0	1	0.874107	---
110	0	1	0.874107	---
111	0	1	0.874107	---
112	0	1	0.874107	---
113	0	1	0.874107	---
114	0	1	0.874107	---
115	0	1	0.874107	---
116	0	1	0.874107	---
117	0	1	0.874107	---
118	0	1	0.874107	---
119	0	1	0.874107	---

120	0	1	0.874107	---
121	0	1	0.874107	---
122	0	1	0.874107	---
123	0	1	0.874107	---
124	0	1	0.874107	---
125	0	1	0.874107	---
126	0	1	0.874107	---
127	0	1	0.874107	---
128	0	1	0.874107	---
129	0	1	0.874107	---
130	0	1	0.874107	---
131	0	1	0.874107	---
132	0	1	0.874107	---
133	0	1	0.874107	---
134	0	1	0.874107	---
135	0	1	0.874107	---
136	0	1	0.874107	---
137	0.007092	1	0.875	0.078771
138	0.007092	1	0.875	0.078771
139	0.007092	1	0.875	0.078771
140	0.007092	1	0.875	0.078771
141	0.007092	1	0.875	0.078771
142	0.007092	1	0.875	0.078771
143	0.007092	1	0.875	0.078771
144	0.007092	1	0.875	0.078771
145	0.007092	1	0.875	0.078771
146	0.007092	1	0.875	0.078771
147	0.007092	1	0.875	0.078771
148	0.007092	1	0.875	0.078771
149	0.007092	1	0.875	0.078771
150	0.007092	1	0.875	0.078771
151	0.007092	1	0.875	0.078771
152	0.007092	1	0.875	0.078771
153	0.007092	0.99966	0.874702	0.064952
154	0.007092	0.99966	0.874702	0.064952
155	0.007092	1	0.875	0.078771
156	0.007092	0.99966	0.874702	0.064952
157	0.007092	0.99966	0.874702	0.064952
158	0.007092	0.99966	0.874702	0.064952
159	0.007092	0.99966	0.874702	0.064952
160	0.007092	0.99966	0.874702	0.064952
161	0.007092	0.99966	0.874702	0.064952

162	0.007092	0.99966	0.874702	0.064952
163	0.007092	0.99966	0.874702	0.064952
164	0.007092	0.99966	0.874702	0.064952
165	0.007092	0.99966	0.874702	0.064952
166	0.007092	0.99966	0.874702	0.064952
167	0.007092	0.99966	0.874702	0.064952
168	0.007092	0.99966	0.874702	0.064952
169	0.007092	0.99966	0.874702	0.064952
170	0.007092	0.99966	0.874702	0.064952
171	0.007092	0.99966	0.874702	0.064952
172	0.007092	0.99966	0.874702	0.064952
173	0.007092	0.99966	0.874702	0.064952
174	0.007092	0.99966	0.874702	0.064952
175	0.007092	0.99966	0.874702	0.064952
176	0.007092	0.99966	0.874702	0.064952
177	0.007092	0.99966	0.874702	0.064952
178	0.007092	0.99966	0.874702	0.064952
179	0.007092	0.99966	0.874702	0.064952
180	0.007092	0.99966	0.874702	0.064952
181	0.007092	0.99966	0.874702	0.064952
182	0.007092	0.99966	0.874702	0.064952
183	0.007092	0.99966	0.874702	0.064952
184	0.007092	0.99966	0.874702	0.064952
185	0.007092	0.99966	0.874702	0.064952
186	0.007092	0.99966	0.874702	0.064952
187	0.007092	0.99966	0.874702	0.064952
188	0.007092	0.99966	0.874702	0.064952
189	0.007092	0.99966	0.874702	0.064952
190	0.007092	0.99966	0.874702	0.064952
191	0.007092	0.99966	0.874702	0.064952
192	0.007092	0.99966	0.874702	0.064952
193	0.007092	0.99966	0.874702	0.064952
194	0.007092	0.99966	0.874702	0.064952
195	0.007092	0.99966	0.874702	0.064952
196	0.007092	0.99966	0.874702	0.064952
197	0.007092	0.99966	0.874702	0.064952
198	0.007092	0.99966	0.874702	0.064952
199	0.007092	0.99966	0.874702	0.064952
200	0.007092	0.99966	0.874702	0.064952
201	0.007092	0.99966	0.874702	0.064952
202	0.007092	0.99966	0.874702	0.064952
203	0.007092	0.99966	0.874702	0.064952

204	0.007092	0.99966	0.874702	0.064952
205	0.007092	0.99966	0.874702	0.064952
206	0.007092	0.99966	0.874702	0.064952
207	0.007092	0.99966	0.874702	0.064952
208	0.007092	0.99966	0.874702	0.064952
209	0.007092	0.99966	0.874702	0.064952
210	0.007092	0.99966	0.874702	0.064952
211	0.007092	0.998979	0.874107	0.047699
212	0.007092	0.998979	0.874107	0.047699
213	0.007092	0.998979	0.874107	0.047699
214	0.007092	0.998979	0.874107	0.047699
215	0.007092	0.998979	0.874107	0.047699
216	0.007092	0.998979	0.874107	0.047699
217	0.007092	0.998979	0.874107	0.047699
218	0.007092	0.998979	0.874107	0.047699
219	0.007092	0.998979	0.874107	0.047699
220	0.007092	0.998979	0.874107	0.047699
221	0.007092	0.998979	0.874107	0.047699
222	0.007092	0.998979	0.874107	0.047699
223	0.007092	0.998979	0.874107	0.047699
224	0.007092	0.998979	0.874107	0.047699
225	0.007092	0.998979	0.874107	0.047699
226	0.007092	0.998979	0.874107	0.047699
227	0.007092	0.998979	0.874107	0.047699
228	0.007092	0.998979	0.874107	0.047699
229	0.007092	0.998979	0.874107	0.047699
230	0.007092	0.998979	0.874107	0.047699
231	0.007092	0.998979	0.874107	0.047699
232	0.007092	0.998979	0.874107	0.047699
233	0.007092	0.998979	0.874107	0.047699
234	0.007092	0.998638	0.87381	0.04169
235	0.007092	0.998638	0.87381	0.04169
236	0.007092	0.998638	0.87381	0.04169
237	0.007092	0.998638	0.87381	0.04169
238	0.007092	0.998638	0.87381	0.04169
239	0.007092	0.998638	0.87381	0.04169
240	0.007092	0.998638	0.87381	0.04169
241	0.007092	0.998638	0.87381	0.04169
242	0.007092	0.998638	0.87381	0.04169
243	0.007092	0.998638	0.87381	0.04169
244	0.007092	0.998638	0.87381	0.04169
245	0.007092	0.998638	0.87381	0.04169

246	0.007092	0.998638	0.87381	0.04169
247	0.007092	0.998638	0.87381	0.04169
248	0.007092	0.998638	0.87381	0.04169
249	0.007092	0.998638	0.87381	0.04169
250	0.007092	0.998638	0.87381	0.04169
251	0.007092	0.998638	0.87381	0.04169
252	0.007092	0.998638	0.87381	0.04169
253	0.007092	0.998638	0.87381	0.04169
254	0.007092	0.998638	0.87381	0.04169
255	0.007092	0.998638	0.87381	0.04169
256	0.007092	0.998638	0.87381	0.04169
257	0.007092	0.998638	0.87381	0.04169
258	0.007092	0.998638	0.87381	0.04169
259	0.007092	0.998638	0.87381	0.04169
260	0.007092	0.998638	0.87381	0.04169
261	0.007092	0.998638	0.87381	0.04169
262	0.007092	0.998638	0.87381	0.04169
263	0.007092	0.998638	0.87381	0.04169
264	0.007092	0.998638	0.87381	0.04169
265	0.007092	0.998638	0.87381	0.04169
266	0.007092	0.998638	0.87381	0.04169
267	0.007092	0.998638	0.87381	0.04169
268	0.007092	0.998638	0.87381	0.04169
269	0.007092	0.998638	0.87381	0.04169
270	0.007092	0.998638	0.87381	0.04169
271	0.007092	0.998638	0.87381	0.04169
272	0.007092	0.998638	0.87381	0.04169
273	0.007092	0.998638	0.87381	0.04169
274	0.007092	0.998638	0.87381	0.04169
275	0.007092	0.998638	0.87381	0.04169
276	0.007092	0.998638	0.87381	0.04169
277	0.007092	0.998638	0.87381	0.04169
278	0.007092	0.998638	0.87381	0.04169
279	0.007092	0.998638	0.87381	0.04169
280	0.007092	0.998638	0.87381	0.04169
281	0.007092	0.998638	0.87381	0.04169
282	0.007092	0.998638	0.87381	0.04169
283	0.007092	0.998638	0.87381	0.04169
284	0.007092	0.998638	0.87381	0.04169
285	0.007092	0.998638	0.87381	0.04169
286	0.007092	0.998638	0.87381	0.04169
287	0.007092	0.998638	0.87381	0.04169

288	0.009456	0.998298	0.87381	0.049766
289	0.009456	0.998298	0.87381	0.049766
290	0.009456	0.998298	0.87381	0.049766
291	0.009456	0.998298	0.87381	0.049766
292	0.009456	0.998298	0.87381	0.049766
293	0.009456	0.998298	0.87381	0.049766
294	0.009456	0.998298	0.87381	0.049766
295	0.009456	0.998298	0.87381	0.049766
296	0.009456	0.998298	0.87381	0.049766
297	0.009456	0.998298	0.87381	0.049766
298	0.009456	0.998298	0.87381	0.049766
299	0.009456	0.998298	0.87381	0.049766
300	0.009456	0.998298	0.87381	0.049766
301	0.009456	0.998298	0.87381	0.049766
302	0.009456	0.998298	0.87381	0.049766
303	0.009456	0.998298	0.87381	0.049766
304	0.009456	0.998298	0.87381	0.049766
305	0.009456	0.998298	0.87381	0.049766
306	0.009456	0.998298	0.87381	0.049766
307	0.009456	0.998298	0.87381	0.049766
308	0.009456	0.998298	0.87381	0.049766
309	0.009456	0.998298	0.87381	0.049766
310	0.009456	0.998298	0.87381	0.049766
311	0.009456	0.998298	0.87381	0.049766
312	0.009456	0.998298	0.87381	0.049766
313	0.009456	0.998298	0.87381	0.049766
314	0.009456	0.998298	0.87381	0.049766
315	0.009456	0.998298	0.87381	0.049766
316	0.009456	0.998298	0.87381	0.049766
317	0.009456	0.998298	0.87381	0.049766
318	0.009456	0.998298	0.87381	0.049766
319	0.009456	0.998298	0.87381	0.049766
320	0.009456	0.998298	0.87381	0.049766
321	0.009456	0.998298	0.87381	0.049766
322	0.009456	0.998298	0.87381	0.049766
323	0.009456	0.998298	0.87381	0.049766
324	0.009456	0.998298	0.87381	0.049766
325	0.009456	0.998298	0.87381	0.049766
326	0.009456	0.998298	0.87381	0.049766
327	0.009456	0.998298	0.87381	0.049766
328	0.009456	0.998298	0.87381	0.049766
329	0.009456	0.998298	0.87381	0.049766

330	0.009456	0.998298	0.87381	0.049766
331	0.009456	0.998298	0.87381	0.049766
332	0.009456	0.998298	0.87381	0.049766
333	0.009456	0.998298	0.87381	0.049766
334	0.009456	0.997617	0.873214	0.041074
335	0.009456	0.997617	0.873214	0.041074
336	0.009456	0.997617	0.873214	0.041074
337	0.009456	0.997617	0.873214	0.041074
338	0.009456	0.997617	0.873214	0.041074
339	0.009456	0.997617	0.873214	0.041074
340	0.009456	0.997617	0.873214	0.041074
